# Biliary drainage in malignant biliary obstruction: an umbrella review of randomized controlled trials

**DOI:** 10.3389/fonc.2023.1235490

**Published:** 2023-09-05

**Authors:** Yaoqun Wang, Ningyuan Wen, Xianze Xiong, Bei Li, Jiong Lu

**Affiliations:** ^1^ Division of Biliary Tract Surgery, Department of General Surgery, West China Hospital, Sichuan University, Chengdu, Sichuan, China; ^2^ Research Center for Biliary Diseases, West China Hospital, Sichuan University, Chengdu, Sichuan, China

**Keywords:** biliary drainage, malignant biliary obstruction, RCTs, GRADE, umbrella review

## Abstract

**Background:**

There are still many controversies about biliary drainage in MBO, and we aimed to summarize and evaluate the evidence associated with biliary drainage.

**Methods:**

We conducted an umbrella review of SRoMAs based on RCTs. Through July 28, 2022, Embase, PubMed, WOS, and Cochrane Database were searched. Two reviewers independently screened the studies, extracted the data, and appraised the methodological quality of the included studies. GRADE was used to evaluate the quality of the evidence.

**Results:**

36 SRoMAs were identified. After excluding 24 overlapping studies, 12 SRoMAs, including 76 RCTs, and 124 clinical outcomes for biliary drainage in MBO were included. Of the 124 pieces of evidence evaluated, 13 were rated “High” quality, 38 were rated “Moderate”, and the rest were rated “Low” or “Very low”. For patients with MBO, ^125^I seeds+stent can reduce the risk of stent occlusion, RFA+stent can improve the prognosis; compared with PC, SEMS can increase the risk of tumor ingrowth and reduce the occurrence of sludge formation, and the incidence of tumor ingrowth in C-SEMS/PC-SEMS was significantly lower than that in U-SEMS. There was no difference in the success rate of drainage between EUS-BD and ERCP-BD, but the use of EUS-BD can reduce the incidence of stent dysfunction. For patients with obstructive jaundice, PBD does not affect postoperative mortality compared to direct surgery. The use of MS in patients with periampullary cancer during PBD can reduce the risk of re-intervention and stent occlusion compared to PC. In addition, we included four RCTs that showed that when performing EUS-BD on MBO, hepaticogastrostomy has higher technical success rates than choledochoduodenostomy. Patients who received Bilateral-ENBD had a lower additional drainage rate than those who received Unilateral-ENBD.

**Conclusions:**

Our study summarizes a large amount of evidence related to biliary drainage, which helps to reduce the uncertainty in the selection of biliary drainage strategies for MBO patients under different circumstances.

## Introduction

Biliary drainage is an important measure for the diagnosis and treatment of biliary diseases, and its main role is to ensure smooth drainage and reduce jaundice in patients with biliary obstruction (1). At the same time, patients with biliary tract infection are conducive to infection control and symptom alleviation (2). The most common cause of biliary obstruction is obstruction caused by malignancies, as well as benign biliary strictures caused by trauma, surgery, autoimmunity, and inflammation (3). In the cases of malignant biliary obstruction, the diagnosis is always made when painless obstructive jaundice develops in the late stage of the disease because of its insidious clinical manifestations. Therefore, only 10%-20% of patients can undergo surgical resection (4, 5).

For inoperable patients, unobstructed drainage, an important measure of palliative treatment, is beneficial for alleviating symptoms and improving prognosis (6, 7). Although preoperative biliary drainage (PBD) is generally not recommended for surgical patients, there are still many situations in which it may be necessary (8–10). Benign biliary strictures may affect liver function and even secondary biliary cirrhosis for a long time. At present, endoscopic treatment is mainly used to relieve the symptoms of obstruction, maintain biliary tract patency for a long time, and maintain liver function (11, 12).

Biliary drainage methods mainly include internal drainage and external drainage. Internal drainage mainly refers to bile duct stent or endoscopic ultrasound-guided bile duct drainage (EUS-BD). External drainage refers to percutaneous transhepatic cholangial drainage(PTCD), endoscopic nasobiliary drainage (ENBD) or postoperative T-tube drainage.

At present, there are still the following disputes about biliary drainage for MBO:

- Do patients with MBO need biliary drainage before operation (13, 14)? What is the best clinical indication for pre-operative biliary drainage (15, 16)?
- What is the best drainage method for MBO? For example, it is not clear which method, of PTBD or ENBD, has the best effect on reducing jaundice before surgery (17). Whether there is a difference in the efficacy and overall incidence of complications between PTBD and EUS-BD remains controversial (18).
- What type of biliary drainage stent should be selected? How best should a stent be placed? And Is it necessary to combine biliary stents with other treatments?

In recent years, many SRoMAs have been published to compare the biliary drainage schemes for MBO. However, the quality of evidence in evidence-based medicine is uneven, which undoubtedly causes difficulties in clinical decision-making (19). Therefore, an umbrella review is needed to summarize and evaluate all types of evidence-based medicine in this field (20). To date, there has never been an umbrella review of evidence-based medical evidence related to biliary drainage schemes. Since the SRoMAs of randomized controlled trials yielded the highest level of evidence quality, we included all SRoMAs of randomized controlled trials related to this topic in order to summarize high-quality evidence to guide clinical treatment.

## Method

### Study design

The umbrella review is a comprehensive review of existing SRoMAs in a certain field, which aims to evaluate and grade the evidence of evidence-based medicine in this field, to provide more advanced evidence support for clinical decision-making (10, 11). To evaluate the efficacy and safety of different biliary drainage schemes in the treatment of MBO, we performed this umbrella review. The protocol of this study has been registered on the PROSPERO website with the registration number: CRD42022349657.

### Selection and exclusion criteria

In a series of clinical studies, the demonstration intensity of randomized controlled trials was the highest. Therefore, our study include SRoMAs based on clinical randomized controlled trials. If there were no SRoMAs in some aspects, we selected specific RCTs as a supplement.

The inclusion criteria were as follows: (1) if there were no SRoMAs in some aspects, we select specific RCTs as supplements based on randomized controlled trials following the PRISMA guidelines. SRoMAs that include both randomized controlled trials and observational studies. We only included subgroups of randomized controlled trials. If there were no SRoMAs in some aspects, we selected specific RCTs as supplements. (2) the subject of this study was malignant biliary obstruction requiring biliary drainage. (3) the methods of bile drainage include, but are not limited to, endoscopic bile drainage and external bile drainage, but do not include surgical resection and surgical bile duct reconstruction. (4) summarizing and reporting the odds ratio (OR), Relative Ratio (RR), Risk Ratio (HR), or Standardized Mean Difference (SMD) and their corresponding 95% confidence interval (CI). (5) if the results of SRoMAs on the same topic were consistent, the citation matrix and corrected coverage area (CCA) were used to screen the best studies (studies with larger data volume and newer publication year were preferred).If the results were inconsistent, they were all included in the subsequent study.(6) There were no restrictions on language types.

Exclusion criteria: (1) not SRoMAs or RCTs; (2) the research topic was not MBO; (3) the research object was not human; (4) Topics related to surgical resection or surgical biliary reconstruction; (5) OR/HR/RR values were not calculated; (6) unable to obtain full text or meeting abstract; (7) Low-quality studies with overlapping content and conclusions.

The literature was screened according to the three steps of title, abstract and full text. Two authors (Yaoqun Wang and Shaofeng Wang) independently screened the studies.

### Literature search strategy

The two authors of this study (Yaoqun Wang and Ningyuan Wen) independently conducted a systematic and comprehensive literature search using Embase, PubMed, Web of Science and Cochrane Database of Systematic Reviews. We searched for SRoMAs related to our topic from the start of the database to July 28, 2022. The detailed retrieval strategies can be found in **Tables S1-1**, **S1-4**. In addition, we also searched the references included in the study, the relevant literature of clinical trials or research registration platform, and grey literature. All differences were settled through negotiation.

### Data extraction

The data included in the literature were independently extracted by Yaoqun Wang and Ningyuan Wen. Any differences between the two datasets were reassessed by Bei Li. For the SRoMAs included in this study, we extracted the basic information data from the literature, the results of the study and bias assessment data. For subsequent data analysis, we also extracted raw data from randomized controlled studies included in these SRoMAs (**Table S4**).

Basic informations:(1) First author; (2)Country; (3)Publication year; (4) Journal name; (5) Original article retrieval time; (6) Total number of included studies; (7) Individual studies design; (8) Diseases type; (9)Total No. of patients; (10)Intervention(No.of cases); (11)

Control(No.of cases); (12)Information of funding.

Results and data:(1) Clinical outcomes; (2) Effect models; (3) Estimated effect values (HR, OR, RR, SMD) and 95% confidence intervals (95% CI); (4) P-value of effect value.

Bias assessment in SRoMAs:(1)Heterogeneity (*I^2^*) and P-value; (2)Small study effect and P-value; (3) Literature quality assessment method of SRoMAs.

Raw data from randomized controlled studies: (1)First author; (2) Publication year; (3)Intervention (No. of Event/Total); (4)Control (No. of Event/Total);(5)HR/RR/OR value and 95%CI(if available).

### Methodological quality evaluation

The methodological quality of each SRoMA included in the umbrella review were evaluated. The authors Yaoqun Wang and Ningyun Wen evaluated the included studies according to the AMSTAR2 scale. AMSTAR2 is a quality evaluation tool used for the systematic evaluation of randomized and non-randomized preventive and curative studies (21, 22). It includes 16 items in total, involving the entire process of system evaluation, such as topic selection, design, registration, data extraction, data statistical analysis, and discussion. AMSTAR2 specifies items 2, 4, 7, 9, 11, 13, and 15 as the critical domains. According to the provisions of AMSTAR2 scale, the quality of literature can be divided into “High”, “Moderate”, “Low” and “Critically low”. The Rating criteria are as follows (22):

- High No or one non-critical weakness.
- Moderate More than one non-critical weakness.
- Low: One critical flaw with or without non-critical weaknesses.
- Critically low: More than one critical flaw with or without non-critical weaknesses.

### Overlapping publications screening

Many SRoMAs have been published in recent years. There may be multiple SRoMAs with the same medical problems. It is inevitable that the original data included in these SRoMAs overlap (23, 24). Including overlapping data in our study will inevitably bias the research results and reduce the credibility of the conclusions (25). For SRoMAs of the same topic, we used a citation matrix and corrected coverage area (CCA) to quantify the degree of data overlap in these SRoMAs (24, 26, 27) (**Tables S3-1**, 3–9). CCA is defined as mild overlap at 0-5%, moderate at 6-10%, height at 11-15%, and very high at > 15% (28). SRoMAs of overlapping data are screened using the following criteria:

- Studies with mild or moderate overlap are retained.
- Those with more than moderate overlap are retained into the studies with the most original studies, the latest publication dates and higher methodological quality.

### Statistical analysis

An umbrella review is not a meta-analysis of existing data, but rather an objective evaluation of existing evidence (29). We extracted data only from the included meta-analyses and did not perform repeat meta-analyses. For each outcome included in these meta-analyses, we used the DerSimonianand-Laird random effect model to recalculate the OR,HR,RR, or

SMD values and theirs corresponding 95%CI, and calculated the p-value. This model consider the heterogeneity within and between studies (30). We used this method to ensure that all aggregate risk ratios were calculated using random effects models and to obtain further information for the subsequent assessment of evidence quality (31). When 95%CI did not contain an invalid value and P < 0.05, the conclusion was considered statistically significant. All statistical analyses were performed using the R software. **Table S10** listed the R codes used for statistical analysis.

Statistical heterogeneity is defined as the variability in effect estimates across the primary studies of a meta-analysis, and may be a consequence of clinical and/or methodological diversity between studies (32). Heterogeneity emerges when the risk estimates differ more than expected between different studies. We recalculated the heterogeneity of each system review and meta-analysis results using Cochran’s Q test and the p-value of the Higgins consistency (*I^2^*) statistic to evaluate the heterogeneity (33). To identify significant heterogeneity, we consider the threshold of Cochran’s Q test (P > 0.10).

The small-study effects is the tendency of the effect estimates

found in smaller studies to be less conservative than those obtained in larger studies (34). Egger’s test was used to detect small study effects. P-value < 0.10 was deemed to be indicative of small study effects.

### Grade of evidence

GRADE was used to evaluate the quality of the evidence. GRADE is a grading method for evidence quality and recommendation strength that was proposed by the GRADE Working Group in 2004 and can be used as GRADE evidence for intervention SRoMAs (35).

- Type of literature research: RCTs were identified as high-quality evidence to support the estimation of intervention effects, whereas observational studies were defined as low-quality evidence (36).
- Upgrade factors:
- Limitations: The limitations of randomized trials include no covert grouping, no blindness, incomplete reporting of patients and outcome events, selective outcome reporting bias, and other limitations (37). If there are no serious limitations, there will be no demotion; if there are serious limitations, there will be a reduction of one level; if there are very serious limitations, there will be a reduction of two levels.
- Inconsistency: The degree of heterogeneity should be judged on the basis of the similarity of point estimates, the degree of overlap of confidence intervals, and statistical criteria, including heterogeneity test and *I^2^*. After discussing the priori hypotheses that may explain the sources of heterogeneity, if there is still great inconsistency in the research results, the level of evidence quality is reduced (38).
- Indirectness: The quality of evidence may decrease when there are significant differences in the populations, interventions, or measurement outcomes considered in the systematic review. If there is no direct comparison, the quality of evidence should be reduced. If there is more than one category of indirect problems, the quality of evidence should be reduced by two levels (39).
- Naccuracy: Checking the 95% confidence interval (CI) is the best way to determine inaccuracies. If the confidence interval is wide, the quality of evidence is reduced by one level (40).
- Publication bias: If the evidence itself has a high risk of publication bias,the quality of the evidence should be lowered by 1 level (41).

ΛDowngrading factor (42):

1.The effect size was large: with estimated effect values 2-5 or 0. 5-0. 2, and there was no reasonable confounding bias, increased by one level; the effect was very large, with estimated effect values > 5 or < 0. 2, and there were no serious problems related to the risk of bias or accuracy, increasing by two levels.
2.Dose-response relationship: increase by two levels.
3.Reasonable residual mixing further supports the inference of a curative effect, increase by one level.

## Results

### Characteristics of this umbrella review

**Figure 1:**
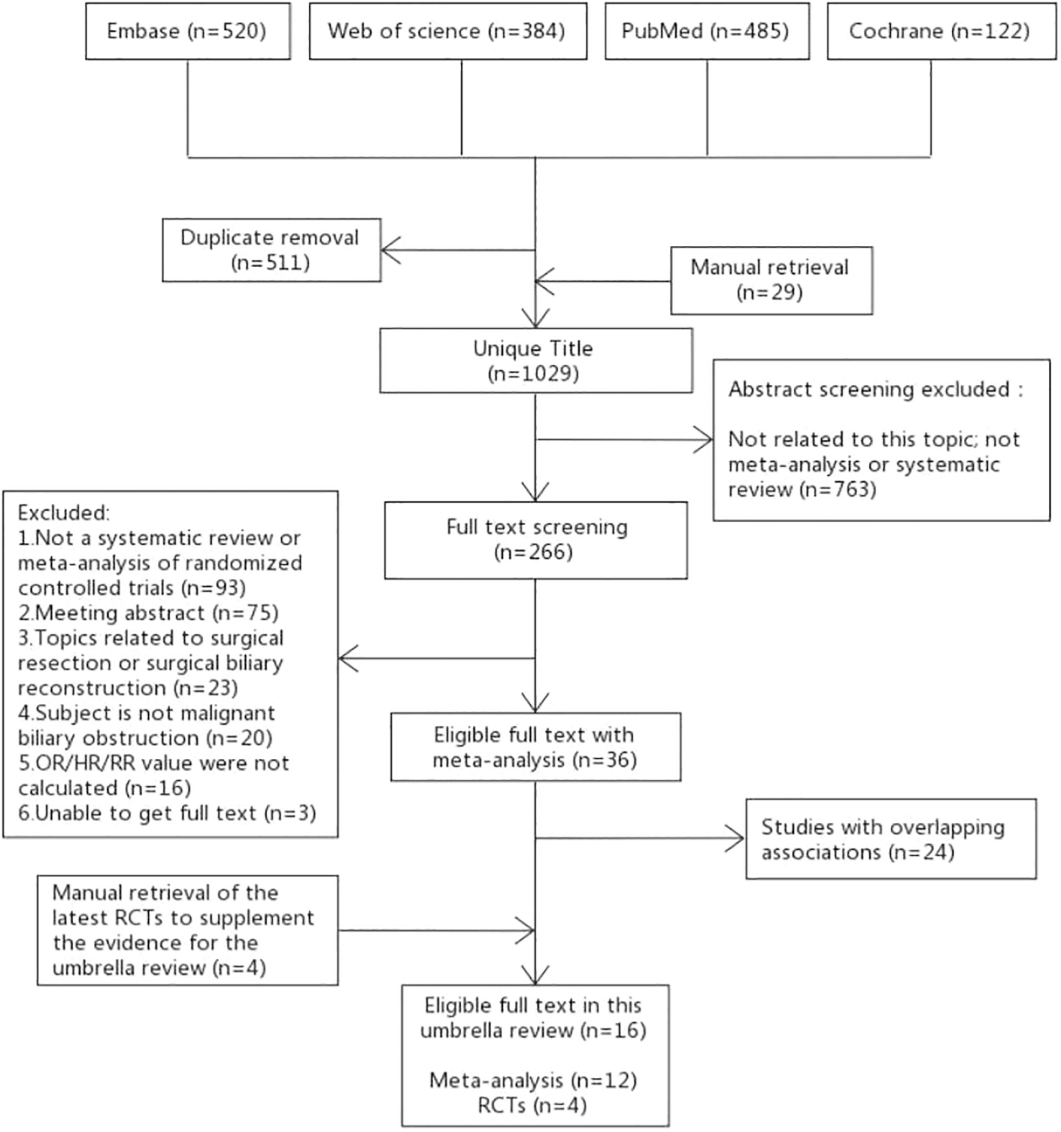
Flow diagram of the selection process.

The specific process of literature selection in this study is shown in **Figure 1**. A total of 1511 articles were retrieved from the four databases in this study, and 29 articles were manually retrieved. A total of 1029 articles were included in title screening after removing duplicate articles. After screening the titles and abstracts, 266 articles were included in full-text screening. The reasons for exclusion of the full-text selected literature and the list of excluded literature are listed in **Table S2**. After the full-text screening, 36 articles met the inclusion criteria. After methodological quality assessment (**Table S5**) and data overlap literature screening (**Table S6**) of the 36 articles, 12 RCT-based SRoMAs were finally included. The inclusion and exclusion lists for 56 references are shown in **Table S7**. Inaddition, there were no SRoMAs in some aspects, therefore, we select the latest four RCTs as supplements.

Our umbrella review contains 124 pieces of evidence, which can be divided into four categories: (1) Can patients with unresectable malignant biliary obstruction benefit from biliary stent combined with other measures (43–45)? (2) Which stent is the best choice for patients with unresectable malignant biliary obstruction (46–48)? (3) Which drainage mode should be chosen for patients with malignant biliary obstruction? Should sphincterotomy be performed (49–57)? (4) Do patients with biliary malignancy and obstructive jaundice need biliary drainage before surgery (58, 59)? Of the 124 pieces of evidence evaluated, 13 were rated “High” quality, 38 were rated “Moderate”, and the rest were rated “Low” or “Very low”.

The other 4 RCTs included a total of 20 pieces of evidence, 2 of which were statistically significant.

### Characteristics of the SRoMAs included

Twelve SRoMAs based on randomized controlled trials and four RCTs were included in this study (43–59). **Tables 1**, **2** shows the basic characteristics of the 16 studies. All the SRoMAs were published between 2006 and 2022. Among them, seven studies were from China, three from Japan, two from the United States, two from Brazil, and the rest from Canada, Italy, and Korea. Eighty RCTs were included in these studies. The number of RCT included in each SRoMAs ranged from 2 to 20, with a minimum of 132 participants and a maximum of 1638 participants. The evidence in each meta-analysis mainly includes two categories: one is the evidence related to clinical success rate, remission rate, and survival; the other is the evidence related to the incidence of various complications. In addition, it also includes some evidence related to clinical economics, such as length of stay and, hospitalization costs. With regard to the quality of the included RCTs studies, except for one meta-analysis (44) that did not evaluate the quality of the literature, the other meta-analyses evaluated the included RCT, and no low-quality studies were found. In addition, we summarized the supporting funds for each SRoMA, and none of the included studies had conflicts of interest among the authors.

**Table 1:** The general characteristics of the 18 systematic reviews and meta-analyses.

First author, year	Country	Journal	Original article retrieval time	Total No. of included studies	Individual studies design	Diseases type	Total No. of patients	Intervention (No.of cases)	Control (No.of cases)	Clinical Outcome	Quality appraisal tool	Information of funding	Conflict of interests
Xiang, 2021 (43)	China	*Journal of Cancer*	2020/5/1	11	RCT	MBO	767	I125seeds+stent(377)	Stent(390)	1.Stent Occlusion 2.Survival	ROB-2	1.the National Natural Science Foundation of China (No. 81572307 and 81773096) 2.the Major Project of Medical and Health Technology Development Program in Zhejiang Province (No. 7211902).	None
Yuan, 2019 (44)	China	*European Journal of Surgical Oncology*	2017/8	3	RCT	MBO	221	PECMS/MSCPM(122)	CMS(99)	1.Stent patency duration 2.Survival time 3.Stent malfunction 4.Stent occlusion caused by tumor in growth 5.Stent occlusion caused by distal stent 6.All complications 7.Pancreatitis 8.Cholangitis-like symptoms	None	1.Shanghai Health and Family Planning Commission Research Foundation (No.201540158). 2.Shanghai Xuhui District Medical Science and Technology Project (No.SHSXH005).	None
Song, 2022 (45)	China	*Surgical Endoscopy*	2021/6/20	3	RCT	MBO	287	RFA+Stent (143)	Stent alone (144)	1.Overall survival 2.Mean survival time 3.Mean stent patency time 4.Stent patency rate at 3 months 5.Stent patency rate at 6 months 6.Alleviation of total bilirubin 7.Alleviation of direct bilirubin 8.Abdominal pain 9.Mild bleeding 10.Cholangitis 11.Pancreatitis	ROB-2; GRADE	1.Fundamental Research Funds for the Central Universities (Grant Nos.2020jb kyzx001, lzu-jbky-2020-kb20). 2.Key Laboratory of Evidence-Based Medicine and Knowledge Translation Foundation of Gansu Province (Grant No. GSEBMKT-2021KFKT03). 3.Key Laboratory of Molecular Diagnosis and Precision Therapy of Surgical Tumors of Gansu Province, Grant Number 2019GSZDSYS06.	None
Almadi, 2017 (46)	Canada	*The American Journal of Gastroenterology*	2015/9	20	RCT	MBO	1638	Self-expandable metal stents(848)	Plastic stents(790)	1.Duration of stent patency 2.Duration of patient survival 3.30day mortality 4.Successful stent insertion 5.Successful biliary drainage 6.Total complication 7.Early complications 8.Late complications 9.Pancreatitis 10.Bleeding 11.Sepsis or cholangitis 12.Stent migration 13.Blockage from sludge 14.Tumor ingrowth 15.Tumor overgrowth 16.Hospital re-admission 17.Rate of re-interventions 18.Mean number of re-interventions 19.1-month symptom free 20.3-month symptom free 21.6-month symptom free 22.12-month symptom free	ROB-2; Jadad; GRADE	Deanship of Scientific Research at King Saud University for funding of this research through the Research Group Project number RGP-279.	None
Renno, 2019 (47)	USA	*Annals of Gastroenterology*	2019/4	3	RCT	MBO	293	ARVMS(147)	SEMS(146)	1.Technical success 2.Clinical success 3.All adverse events 4.Early adverse events 5.Late adverse events 6.Overall stent dysfunction 7.Stent migration 8.Stent occlusion	ROB-2	No funding	None
Tringali, 2018 (48)	Italy	*Endoscopy*	2016/11	11	RCT	MBO	1272	Covered-SEMS(643)	Uncovered-SEMS(629)	1.Stent failure 2.Patient mortality 3.Stent migration 4.Tumor ingrowth 5.Tumor overgrowth 6.Sludge formation 7.Cholecystitis 8.Cholangitis 9.Pancreatitis 10.Perforation 11.Bleeding	ROB-2	No funding	None
Duan, 2017 (49)	China	*Cancer Imaging*	2017/2/28	3	RCT	MBO	183	PTBD(91)	EUS-BD(92)	1.Therapeutic success rate 2.Overall complication 3.30-day mortality rate 4.Cholangitis	Jadad	No funding	None
Sharaiha, 2017 (50)	USA	*Gastrointestinal Endoscopy*	2016/9/4	3	RCT	MBO	132	PTBD(65)	EUS-BD(67)	1.Technical success 2.Clinical success 3.Postprocedure adverse events 4.Rate of re-intervention 5.Length of stay in hospital	ROB-2	Dr. Michel Kahaleh has received grant support from Boston Scientific, Olympus and Gore.	None
Logiudice, 2019 (51)	Brazil	*World Journal of Gastrointestinal Endoscopy*	2018/11	3	RCT	MBO	222	EUS-BD(112)	ERCP-BD(110)	1.Technical success 2.Clinical success 3.Duration of the procedure 4.Adverse events 5.Stent patency 6.Stent dysfunction	Jadad; GRADE	No funding	None
Cui, 2014 (52)	China	*World Journal of Gastroenterology*	2014/3	3	RCT	MBO	338	EST(170)	non-EST(168)	1.Successful stent insertion 2.PEP 3.Post-ERCP bleeding 4.Stent migration 5.Stent occlusion	Jadad	No funding	None
Fang, 2013 (58)	China	*Cochrane Database of Systematic Review*	2012/2	6	RCT	Obstructive Jaundice-PBD	520	PBD(265)	non-PBD(255)	1.Mortality 2.Long-term mortality 3.Serious morbidity 4.Hospital stay	ROB-2; GRADE	No funding	None
Watanabe, 2022 (59)	Japan	*Journal of Hepato-Biliary-Pancreatic Sciences*	2022/1	7	RCT	Periampullary Cancer-PBD	440	PBD-Metal stent (230)	PBD-Plastic stent(210)	1.Re-intervention 2.PBD-related complications 3.Postoperative complications 4.Direct costs 5.Stent occlusion 6.Preoperative cholangitis 7.Preoperative pancreatitis 8.Operative times 9.Blood loss volumes 10.Postoperative pancreatic fistulas 11.Delayed gastric emptying 12.Wound infection 13.Postoperative bleeding	ROB-2; GRADE	No funding	None

RCT, randomized controlled trials; MBO, malignant biliary obstruction; PTBD, percutaneous transhepatic biliary drainage; EUS-BD, endoscopic ultrasound-guided biliary drainage; PECMSs, paclitaxel-eluting covered metal stents; MSCPMs, metallic stents covered with a paclitaxel incorporated membrane; CMSs, conventional covered metal stents; SEMS, self-expandable metal stent; ARVMS, antireflux valve metal stent; TRBO, time to recurrent biliary obstruction; ROB, recurrent biliary obstruction; EST, endoscopic sphincterotomy; ERCP, endoscopic retrograde cholangiopancreatography; PEP, post-endoscopic retrograde cholangiopancreatography pancreatitis; ROB-2, The revised Cochrane Risk of Bias Tool; GRADE, Grading of Recommendations Assessment, Development, and Evaluation; PBD, preoperative biliary drainage; cSEMS, covered self-expandable metal stents; MPS, multiple plastic stents.

**Table 2:** The general characteristics of the 4 included RCTs.

First author, year	Country	Journal	studies design	Diseases type	Total No. of patients	Intervention (No.of cases)	Control (No.of cases)	Clinical Outcome	Information of funding	Conflict of interests
Minaga, 2019 (54)	Japan	*Digestive Endoscopy*	RCTs	MBO	47	Hepaticogastrostomy (24)	Choledochoduodenostomy (23)	1.Technical success rates; 2.Clinical success rates; 3.Mean procedure time; 4.Mean time to oral intake; 5.Overall adverse events rate; 6.Reintervention rate; 7.Median stent patency time; 8.Median survival.	Japan Society for the Promotion of Science (grant no. 16K09410)	None
Ryunosuke,2021 (55)	Japan	*Journal of Clinical Medicine*	RCTs	MBO	77	Unilateral-ENBD(38)	Bilateral-ENBD(39)	1.Technical success; 2.Functional success; 3.Additional drainage; 4.Early adverse events.	None	None
Choi, 2022 (56)	Korea	*Hepatobiliary & Pancreatic Diseases International*	RCTs	MBO	52	Acetylsalicylic acid+ STENT(24)	Placebo+STENT(28)	1.Stent dysfunction; 2.Duration of stent patency.	Institutional Review Board of Seoul National University Hospital (H-1707- 161-874) and Wonju Severance Christian Hospital (CR117068) and was registered with ClinicalTrials.gov (NCT03279809).	None
Fu, 2019 (57)	China	*Abdominal Radiology*	RCTs	MBO	72	Unilateral STENT (36)	Bilateral STENT (36)	1.Technical success rate; 2.Clinical success rates; 3.Stent dysfunction; 4.Overall adverse events; 5.Stent patency(days); 6.Overall survival.	None	None

### Appraise of methodological quality

The AMSTAR2 scale was used to evaluate the methodological quality of the 36 articles (**Table S5**). According to the standard of the AMSTAR2 scale (22), if there is more than one critical flaw in the literature, its methodological quality will be rated as critically low. Because most studies did not register protocols in advance, and did not provide a literature exclusion list, they did not meet the key items 2 and 7 of the AMSTAR2 scale, so their methodological quality were directly rated as critically low. Overall, 31 articles were rated as critically low, 4 as low and 1 as high.

### Combination treatment of biliary stents for unresectable MBO patients

**Figure 2:**
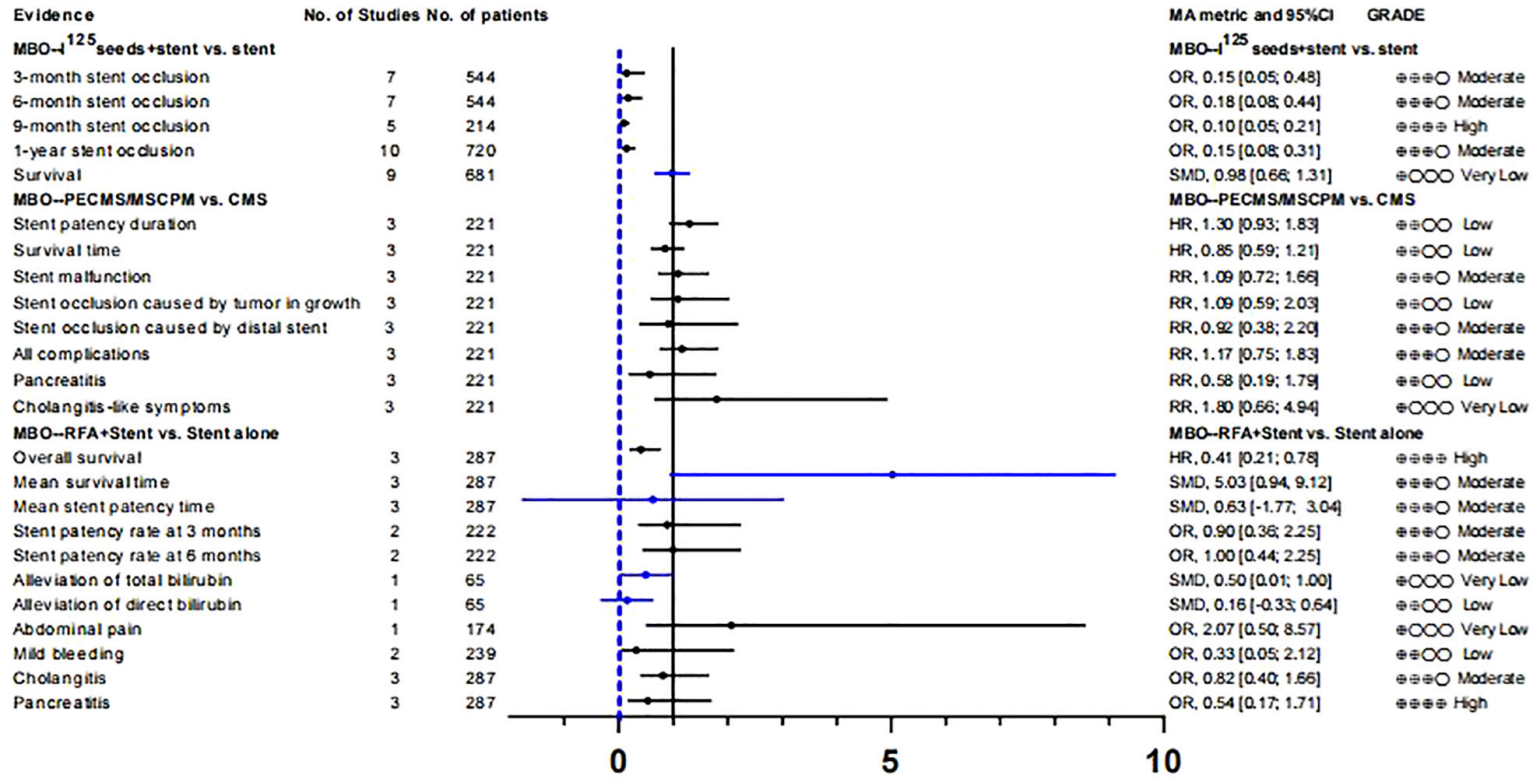
Combination treatment of biliary stents for unresectable MBO patients.

^125^I seeds can directly kill tumor cells and cause less damage to normal tissues and cells owing to their short effective radiation radius, low initial dose, and short radioactive half-life. Therefore, it can be used for internal radiation therapy in patients with malignant tumors (60, 61). A meta-analysis by Xiang et al. (43) investigated the therapeutic efficacy and safety of ^125^I+ biliary stents compared to stents alone in the treatment of MBO. After evaluation, we found that although the results showed that the curative effect of 125i + biliary stent was significantly better than that of the control group, there was significant heterogeneity and small study effects in all outcomes except “9-month stent occlusion” (OR, 0.10 [0.05; 0.21]). Therefore, only the evidence quality of “9-month stent occlusion” was rated as “High”, the rest of the evidence related to “stent occlusion” was rated as “Moderate”, and the evidence related to “survival” was rated as “Very low” (**Tables S8, 9**; **Figure 2**).

Yua et al. (44) studied the role of paclitaxel-loaded metal stents (PECMSs) in the treatment of MBO. However, after our recalculation and evaluation, we did not find any statistically significant conclusions, and there was no higher level of evidence(**Tables S8, 9**; **Figures 2**, **1**). Therefore, we believe that there is no evidence that patients with MBO benefit from paclitaxel combined with biliary stents.

As for radiofrequency ablation(RFA) combined with biliary stents in the treatment of MBO (45), we found that in terms of overall survival (HR, 0.41 [0.21; 0.78]; GRADE: High) and mean survival time (SMD, 5.03 [0.94,9.12]; GRADE: Moderate), the combination therapy significantly improved the prognosis of MBO patients without increasing the incidence of adverse reactions. However, there was no significant improvement in stent patency or jaundice (**Tables S8, 9**; **Figure 2**).

### Selection of stents for unresectable MBO patients

**Figure 3:**
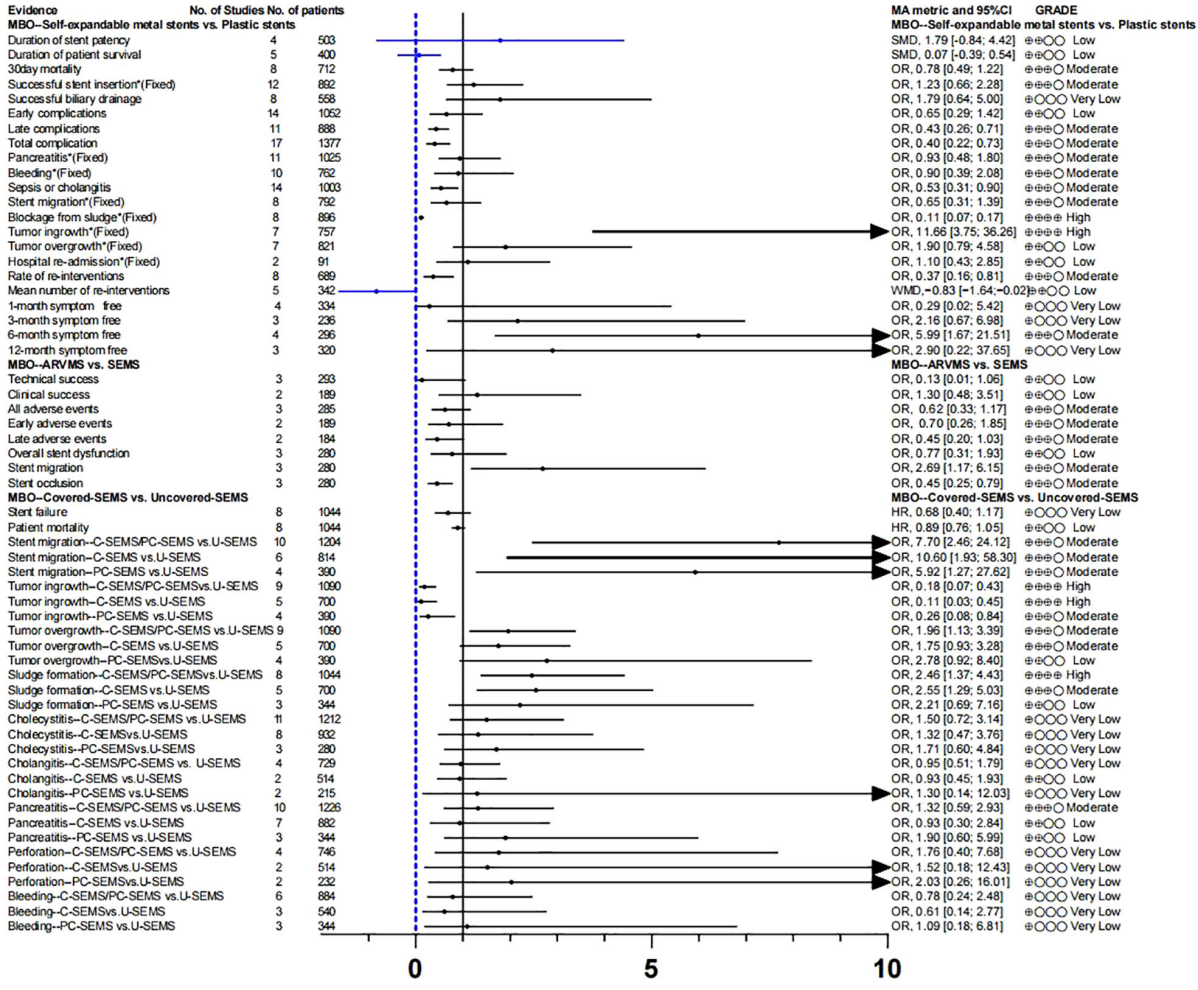
Selection of stents for unresectable MBO patients.

Endoscopic plastic biliary stent drainage (endoscopic retrograde biliary drainage, ERBD) is a common method for endoscopic treatment of bile duct stricture. Metal bile duct stents are mainly used for the treatment of unresectable malignant bile duct strictures or obstruction (62). We compared the advantages and disadvantages of self-expanding metal stents (SEMS)and plastic stent in MBO (46), and found no difference in stent patency rate, survival time, clinical success rate, and early complications. We found that self-expanding metal stents increased the risk of tumor ingrowth (OR,11.66[3.75; 36.26]; GRADE: High), decreased the incidence of sepsis or cholangitis (OR, 0.53 [0.31; 0.90]; GRADE: Moderate) and blockage from sludge (OR, 0.11 [0.07; 0.17]; GRADE: High), reduced the rate of re-interventions (OR, 0.37 [0.16; 0.81]; GRADE: Moderate)and improved the 6-month symptom free ratio (OR, 5.99 [1.67; 21.51]; GRADE: Moderate) (**Tables S8, 9**; **Figure 3**).

Anti-reflux valve metal scaffolds (ARVMS) are based on SEMS to add valves of different shapes to prevent bile reflux and reduce the risk of cholangitis (63). Some studies have compared the safety and efficacy of ARVMS and SEMS in patients with MBO. However, the superiority of these two stent types is still controversial (63–65). The meta-analysis data we summarized showed that (47), the risk of stent migration in ARVMS was significantly increased (OR, 2.69 [1.17; 6.15]; GRADE: Moderate), while the risk of stent occlusion (OR, 0.45 [0.25; 0.79]; GRADE: Moderate) was lower than that in SEMS. However, there was no significant difference in success rate and adverse reactions between the two groups (**Tables S8, 9**; **Figure 3**).

SEMS can be divided into covered SEMS (C-SEMS), partially covered SEMS (PC-SEMS)and uncovered SEMS (U-SEMS) according to whether they are covered or not. We further evaluated the differences in efficacy and safety of these types of stents for MBO (48). Overall, stent failure and patient mortality were not significantly different between C-SEMS/PC-

SEMS and U-SEMS. In terms of complications, the incidence of tumor ingrowth in the C-SEMS/PC-SEMS group was significantly lower (OR, 0.18 [0.07; 0.43]: GRADE : High), and the risk of stent migration (OR, 7.70 [2.46; 24.12]; GRADE: Moderate) was higher than that in the U-SEMS group. Tumor overgrowth (OR, 1.96 [1.13; 3.39]; GRADE: Low) and sludge formation (OR, 2.46 [1.37; 4.43]; GRADE: Moderate) showed high risk in the C-SEMS/PC-SEMS group, the quality of evidence was degraded due to inconsistency between subgroups, so we have reservations about this conclusion

(**Tables S8, 9**; **Figure 3**).

### Biliary drainage in MBO patients: PTBD, EUS-BD or ERCP-BD

**Figure 4:**
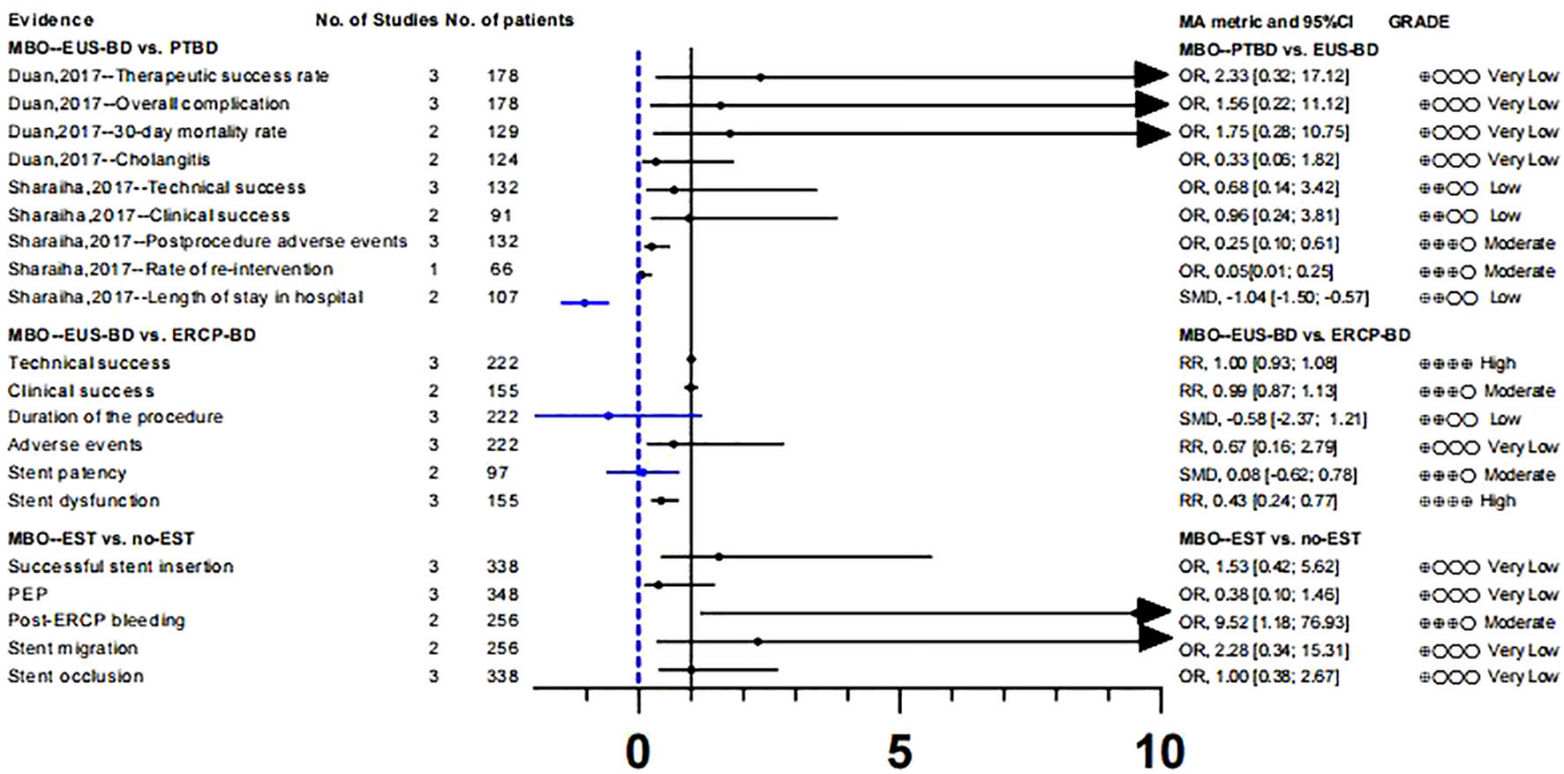
Biliary drainage in MBO patients: PTBD, EUS-BD or ERCP-BD.

To study whether there are differences in the efficacy and overall incidence of complications between PTBD and EUS-BD, we included two meta-analyses (49, 50). These two literatures included three RCTs respectively, and there was no data overlap (**Table S3**). According to our calculation and evaluation, there was no difference in the clinical success rate and mortality between PTBD and EUS-BD, and the quality of evidence for these outcomes was low. In terms of postoperative complications, only the outcomes reported by Sharaiha et al. (50) were statistically different(Postprocedure adverse events;OR, 0.25 [0.10; 0.61];GRADE: Moderate), suggesting a better safety profile for EUS-BD than for PTBD. In addition, sharaiha et al. (50) also reported that the rate of re-intervention and length of stay in hospitals were lower than in PTBD, however, their evidence quality was lower (**Tables S8, 9**; **Figure 4**).

In comparing ERCP-BD with EUS-BD (51), we found that there was no difference in success rate(technical success: GRADE : High; Clinical success: GRADE: Moderate) and stent patency(GRADE: Moderate), but the Stent dysfunction (RR, 0.43 [0.24; 0.77]; GRADE: High) in the EUS-BD group was lower than that in the ERCP-BD group (**Tables S8, 9**; **Figure 4**).

In MBO patients, we also analyzed the impact of EST before ERCP stent placement on various clinical outcomes (52). A randomized controlled study (66) suggested that the purpose of EST before stent placement was to reduce the incidence of PEP and make stent placement easier. After recalculation, we only found that the risk of post-ERCP bleeding (OR, 9.52 [1.18; 76.93]; GRADE: Moderate) was significantly increased in the EST group. The other clinical outcomes such as successful stent insertion, PEP, stent migration and stent occlusion were not significantly different. At the same time, the level of the evidence is very low, and its authenticity needs to be studied (**Tables S8, 9**; **Figure 4**).

### Preoperative biliary drainage in patients with obstructive jaundice or biliary cancer

**Figure 5:**
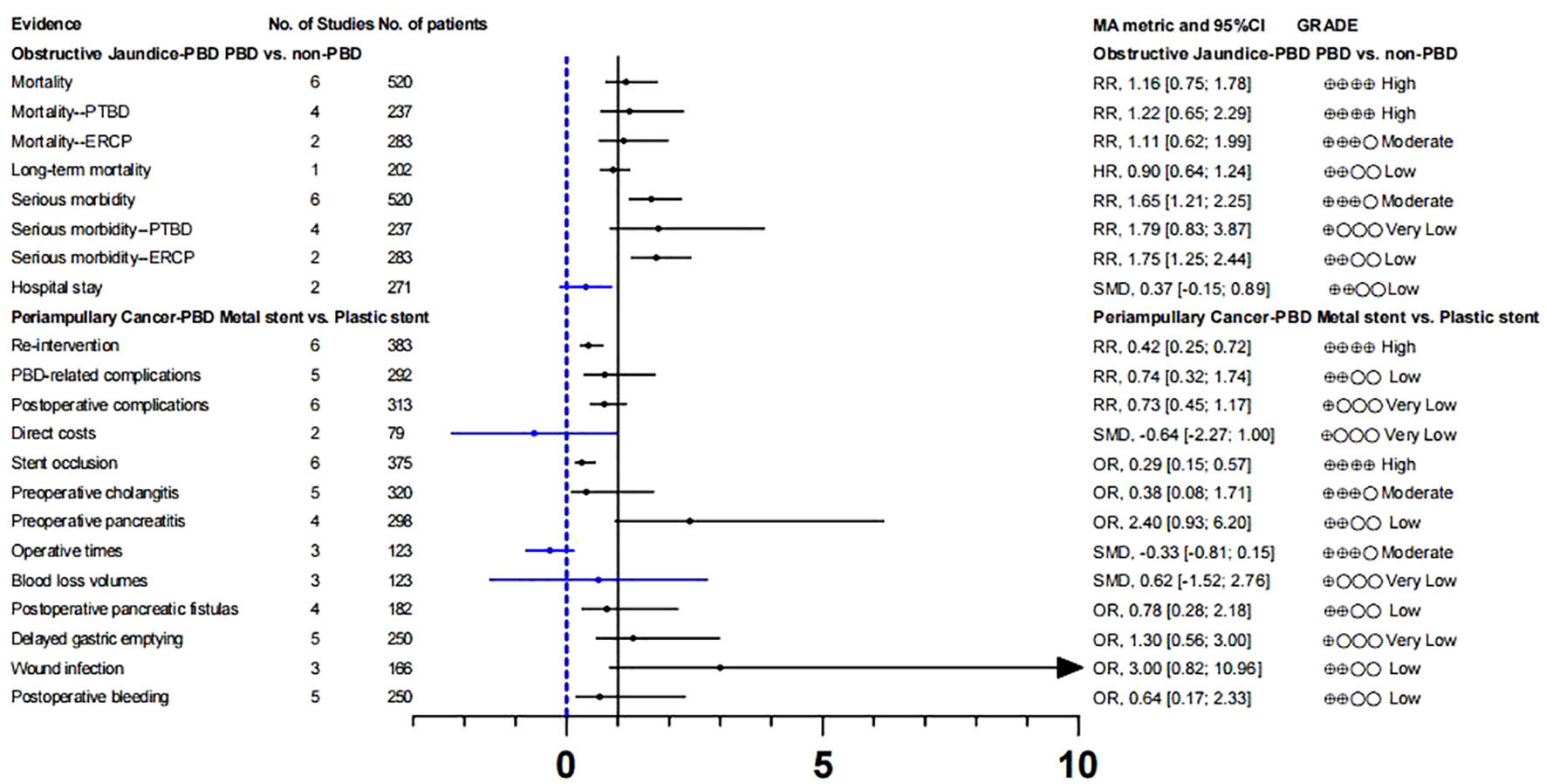
Preoperative biliary drainage in patients with obstructive jaundice or biliary cancer.

Whether patients with resectable obstructive jaundice require endoscopic bile drainage before surgery remains controversial. Studies have suggested that preoperative biliary drainage will increase the risk of bacteremia, fungal translocation, postoperative septicemia and wound infection, as well as hospital stay and total cost (13, 14). Fang et al. (58) conducted a meta-analysis to address these issues, which included six RCTs. The experimental group underwent PTBD or ERCP-BD for preoperative drainage, while the control group underwent direct surgery without drainage. There was no significant difference in postoperative mortality (GRADE: High) between preoperative bile drainage and direct surgery. Although PBD increases serious morbidity(RR, 1.65 [1.21; 2.25]; GRADE: Moderate), we suggest caution with this conclusion because of the limited number of studies and the inconsistency between subgroups. With respect to hospital stay, our calculations did not find a statistically significant difference between the two groups, and the strength of evidence for this conclusion was low(**Tables S8, 9**; **Figure 5**).

Recent guidelines recommend the use of metal stents for PBD in patients with periampullary cancer who have received long-term neoadjuvant chemotherapy and have a long waiting time for surgery (67, 68). However, the level of evidence supporting this recommendation is very low. In addition, some studies suggest that plastic stents should be used for PBD in patients with malignancies with recurrent cholangitis or jaundice when the preoperative waiting time is not too long (69). Therefore, we analyzed the clinical outcomes of periampullary cancer patients who underwent preoperative biliary drainage in recent years and further evaluated the efficacy and safety of metal stents and plastic stents on PBD in periampullary cancer patients (59). Among the clinical outcomes we included, there were only two pieces of high-quality evidence: re-intervention (RR,0.42 [0.25; 0.72]) and stent occlusion (OR, 0.29 [0.15; 0.57]).

This suggests that preoperative use of metal stents can effectively reduce the rate of re-intervention and the risk of stent occlusion. In addition, two pieces of moderate-level evidence showed that there was no statistically significant in preoperative cholangitis (OR, 0.38 [0.08; 1.71]) and operative times (SMD, -0.33 [-0.81; 0.15]]) between the two stents. The clinical reference value of other clinical outcomes is limited owing to the low quality of evidence. (**Tables S8, 9**; **Figure 5**).

### Evidence summary from 5 randomized controlled trials

Since only including evidence from SRoMAs may result in missing evidence in some aspects, we also included the four latest RCTs (**Tables 2**, **3**). The evidence involved in these RCTs has not yet been summarized by SRoMAs. Regarding EUS-BD for MBO patients, the two most commonly used methods for EUS-BD in MBO patients are Hepaticogastrostomy and Choledochoduodenostomy. An RCT conducted by Minaga et al. (54) showed that the two methods have no significant differences in other aspects, except that the former is superior to the latter in terms of technical success rates(HGS vs. CDS= 87.5% vs. 82.6%,p= 0.028).

**Table 3:** Evidence summary of included RCTs.

First author,year	Treatment	Evidence	No. of patients	Clinical Outcome	P-value
Minaga, 2019 (54)	Hepaticogastrostomy vs. Choledochoduodenostomy	Technical success rates	47	87.5% vs. 82.6%	0.028
Minaga, 2019 (54)	Hepaticogastrostomy vs. Choledochoduodenostomy	Clinical success rates	47	100% vs. 95.7%	0.489
Minaga, 2019 (54)	Hepaticogastrostomy vs. Choledochoduodenostomy	Mean procedure time	47	39.2±12.2 vs. 30.5±15.8 (minutes)	0.101
Minaga, 2019 (54)	Hepaticogastrostomy vs. Choledochoduodenostomy	Mean time to oral intake	47	3.13±2.45 vs. 4.48±1.09 (days)	0.228
Minaga, 2019 (54)	Hepaticogastrostomy vs. Choledochoduodenostomy	Overall adverse events rate	47	25% vs. 17.4%	0.524
Minaga, 2019 (54)	Hepaticogastrostomy vs. Choledochoduodenostomy	Reintervention rate	47	16.7% vs. 4.3%	0.171
Minaga, 2019 (54)	Hepaticogastrostomy vs. Choledochoduodenostomy	Median stent patency time	47	306 vs. Not reached (days)	0.593
Minaga, 2019 (54)	Hepaticogastrostomy vs. Choledochoduodenostomy	Median survival	47	145 (21–400) vs. 122 (24–408) (days)	0.644
Ryunosuke, 2021 (55)	Unilateral-ENBD vs. Bilateral-ENBD	Technical success	77	100% vs. 95%	0.49
Ryunosuke, 2021 (55)	Unilateral-ENBD vs. Bilateral-ENBD	Functional success	77	57% vs. 56%	0.99
Ryunosuke, 2021 (55)	Unilateral-ENBD vs. Bilateral-ENBD	Additional drainage	77	39% vs. 5.3%	<0.001
Ryunosuke, 2021 (55)	Unilateral-ENBD vs. Bilateral-ENBD	Early adverse events	77	19% vs. 31%	0.30
Choi, 2022 (56)	Acetylsalicylic acid+STENT vs. Placebo+STENT	Stent dysfunction	52	OR, 1.445 (0.365–5.724)	0.600
Choi, 2022 (56)	Acetylsalicylic acid+STENT vs. Placebo+STENT	Sstent patency	52	HR, 1.344 (0.388–4.653)	0.641
Fu, 2019 (57)	Unilateral STENT vs. Bilateral STENT	Technical success rate	60	83.3% vs. 83.3%	1.000
Fu, 2019 (57)	Unilateral STENT vs. Bilateral STENT	Clinical success rates	60	90% vs. 96.7%	0.605
Fu, 2019 (57)	Unilateral STENT vs. Bilateral STENT	Stent dysfunction	60	16.7% vs. 10.0%	0.704
Fu, 2019 (57)	Unilateral STENT vs. Bilateral STENT	Overall adverse events	60	10.0% vs. 10.0%	1.000
Fu, 2019 (57)	Unilateral STENT vs. Bilateral STENT	Stent patency	60	118 vs. 125 (days)	0.571
Fu, 2019 (57)	Unilateral STENT vs. Bilateral STENT	Overall survival	60	HR, 0.949 (0.558–1.613)	0.845

For MBO patients who planned to undergo ENBD drainage, although there was no statistical difference in the technical success rate, functional success rate, and early adverse events between unilateral-ENBD and bilateral-ENBD, the incidence of additional drainage in patients receiving bilateral-ENBD was significantly lower than that in patients with unilateral-ENBD (Unilateral-ENBD vs. bilateral-ENBD= 39% vs. 5.3%,p<0.001) (55). In addition, for biliary stent treatment, recent RCTs suggest that acetylsalicylic acid dose not improve stent dysfunction, nor will it prolong stent patency (56); for MBO patients who are scheduled to undergo biliary stent drainage, the two methods of unilateral stent and bilateral stent were not significantly difference in all aspects (57).

## Discussion

### Main finding and discussion

We conducted an umbrella review of biliary drainage for MBO. In our study, the evidence associated with biliary drainage in MBO from available SRoMAs was summarized, and the strength and validity of the evidence were evaluated. There were 12 published SRoMAs, and 4 RCTs were included, with a total of 144 pieces of evidence about different biliary drainage schemes for MBO. Only one SRoMA was rated as high in methodological quality, not as medium studies, four as low, and the rest as extremely low. Of the 124 pieces of evidence from SRoMAs assessed, 13 were rated “High” quality, 38 were rated “Moderate”, and the rest were rated “Low” or “Very low”.

In the meta-analysis of ^125^I+ stents for MBO (43), we considered only evidence that it reduced the risk of stent occlusion to be credible. Whether this combination would prolong survival was too low a quality of evidence to be determined further. Furthermore, we were unable to further assess the safety of this regimen because the study did not quantify its complications. Recent randomized controlled studies have shown (70–73) that this combination therapy can prolong patient survival without increasing the risk of complications compared to stent implantation alone. Therefore,it is necessary to conduct an updated meta-analysis to obtain more stronger evidence.

Drug-eluting stents were first used to reduce the rate of stent failure in coronary artery-related diseases. Currently, there are only a few studies on biliary stents. A meta-analysis included in our umbrella review summarized the application of paclitaxel drug-eluting stents in MBO (44). However, there is no evidence that the efficacy and safety of this type of stent are significantly different from those of metal stents alone.

A recent randomized controlled study on the treatment of MBO with paclitaxel-eluting stents (74) showed that the new paclitaxel-eluting biliary metallic stent (MSCPM-III) did not improve the survival of MBO patients and the time of recurrence of biliary obstruction, but the use of MSCPM-III reduced the tumor volume and did not increase the risk of complications. It is well known that the first-line chemotherapy regimen for biliary malignancies is gemcitabine + cisplatin. Although there are no report on the application of such chemotherapeutic drug-eluting stents in humans, a recent study (75) showed that the safety of gemcitabine eluting stents has been confirmed in animal experiments and may be applied in clinical practice in the future.

Radiofrequency ablation (RFA) is usually combined with endoscopic stent insertion, which may improve survival rate and stent patency in patients with MBO. In our study, we believe that combination therapy significantly improves the prognosis of patients with MBO but does not prolong stent patency time or increase the incidence of cholangitis and pancreatitis (45). However, the latest randomized controlled study on the treatment of MBO with U-SEMS + RFA showed that (76), this combined treatment had no positive effect on stent patency or survival rates. Since the study by Song et al. (45) included different types of stents, we believe that it is necessary to investigate whether different types of stents combined with radiofrequency differ in their efficacy in MBO patients.

As for the choice of metal stent and plastic stent, base on the evidence we summarized (46), there was no significant difference between them in the main clinical outcomes, such as stent patency rate, survival time, mortality, and symptom-free rate, but the quality of evidence was not high. Metal stent only showed an advantage in 6-month symptom-free rate. The two groups have their own characteristics in terms of different complication rates.As far as plastic stents are concerned, the average patency period is approximately 3-6 months. Once a plastic stent is blocked, timely replacement should be considered (77). For patients with high intrahepatic bile duct obstruction, plastic stents should be used cautiously when the drainage area is very limited, otherwise, they may lead to severe bile duct infection (77). As for metal stents, previous studies have shown that the patency rate of metal stents is significantly higher than that of plastic stents. For patients with an expected survival time of more than 6 months, implantation of metal stents will reduce the number of ERCP procedures, shorten hospital stay and reduce complications (78–80). Metal stents should not be placed if the tumor shows invasive growth or is complicated by bile duct tumor thrombus or high-risk bile duct obstruction with secondary bile duct invasion (77).

In the study of malignant distal bile duct obstruction, we identified two pieces of credible evidence (48): the use of C-SEMS/PC-SEMS can significantly reduce the incidence of tumor ingrowth, but increase the risk of stent migration. A recent meta-analysis also explored the same topic (53), which was not included in our study because it did not conduct a subgroup analysis of randomized controlled studies, but the conclusions of this study are still worthy of reference. The study found that, although there was no difference in the incidence of recurrent biliary obstruction (ROB) between C-SEMS and U-SEMS, the time of RBO in C-SEMS was significantly prolonged. This suggests that C-SEMS is superior to U-SEMS in preventing RBO in patients with malignant distal bile duct obstruction.

ERCP-BD is the standard treatment for unresectable malignant bile duct obstructions. When patients with MBO fail to undergo ERCP, other drainage methods, such as PTBD or EUS-BD can be used as alternative drainage options. Studies have found that EUS-BD is superior to PTBD in terms of the incidence of postoperative adverse reactions and rate of re-intervention (49). At present, there are few randomized controlled studies on this topic, therefore, it is impossible to further analyze whether there are differences between different EUS-BD procedures, as well as between different EUS-BD procedures and PTBD. Recently, with the progress in EUS-BD technology, studies have begun to compare the efficacy and safety of EUS-BD and ERCP-BD in the treatment of MBO. Two pieces of high-level evidence obtained in this study suggest that there is no difference in the technical success rate between the two methods, however, EUS-BD can reduce the incidence of stent dysfunction. Unfortunately, none of these studies has been designed to compare different EUS-BD procedures. Recently, an ongoing RCT project compares EUS-guided choledochoduodenostomy with first-line ERCP (81). It is expected that more studies are expected to confirm the value of EUS-BD in first-line application in the future.

Some studies have suggested that preoperative biliary drainage will increase the risk of bacteremia, fungal translocation, postoperative septicemia, and wound infection, as well as hospital stay and total

cost (13, 14). However, recent studies have shown that PBD can reduce the overall incidence of postoperative complications in patients with malignant obstructive jaundice and optimize patient conditions for radical HC resection (15, 16). We are confident that PBD for resectable MBO dose not increase mortality. To determine whether this will increase the risk of postoperative complications, more high-quality RCTs are needed. In addition, the type of patients and the clinical features or indicators that can be used as the best indication for preoperative biliary drainage in patients with MBO, it remains to be studied.

### Strengths and limitations

The present study has several strengths. First, our study synthesized evidence from the existing available published meta-analyses based on RCTs. Second, citation matrix and corrected coverage area (CCA) combined with screening criteria and AMSTAR2 scale were used for literature screening, and the latest, most abundant and high-quality literature was included, and the impact of data overlap on research conclusions was minimized to the greatest extent. Third, we use the DerSimonian and -Laird random effect model to recalculate the data, and the data are reliable. Fourth, we conducted an Eegger test to detect publication bias in the data. Finally, we used GRADE to classify the quality of evidence, which can be combined with the strength of evidence to provide reasonable suggestions.

However, there are some limitations to our study. First, it only focused on the conclusions of randomized controlled studies and did not include a meta-analysis of retrospective studies, which may lead to incomplete evidence included. Next, because the original data of some studies were not available, we could not recalculate, but this did not have a significant impact on the conclusion. Because of the rigorous items of the AMSTAR 2 instrument, most of the studies we included were rated as low or very low. Lastly,we were unable to use GRADE to assess evidence from the four RCTs included in this study. The validity of this evidence needs to be further evaluated using more clinical research data.

## Conclusion

In general, although a large number of systematic reviews and meta-analyses on biliary drainage have been published, there is still little high-quality evidence on whether biliary drainage, should be performed, the mode of biliary drainage and the choice of biliary stents. Therefore, there is still controversy. In addition, for more detailed questions, such as the best location of drainage stents for malignant biliary obstruction and what specific indicators suggest biliary drainage for biliary tumors before surgery, there is still a lack of randomized controlled trials and meta-analyses to provide high quality evidence. Further research is required in this regard.

## Author contributions

(i) Conception and design: YW, JL, BL. (ii) Administrative support: BL, JL, XX. (ii) Collection and assembly of data: YW, NW. (iii) Data analysis and interpretation: YW. (iv) Manuscript writing: YW, NW. (v) Final approval of manuscript: All authors. All authors contributed to the article and approved the submitted version.

## Glossary

**Table:**

ARVMS	antireflux valve metal stent
BBS	benign biliary strictures
CCA	corrected coverage area
CDS	Choledochoduodenostomy
CMSs	conventional covered metal stents
CoT	conservative treatment
C-SEMS	covered SEMS
ENBD	endoscopic nasobiliary drainage
ERBD	endoscopic retrograde biliary drainage
ERCP	endoscopic retrograde cholangiopancreatography
EST	endoscopic sphincterotomy
EUS-BD	endoscopic ultrasound-guided biliary drainage
GRADE	grading of recommendations assessment development and evaluation
GSP	gallstone pancreatitis
HGS	hepaticogastrostomy
HR	hazard ratio
MBO	malignant biliary obstruction
MPS	multiple plastic stents
MS	metal stents
MSCPMs	metallic stents covered with a paclitaxel incorporated membrane
OR	odds ratio
PBD	preoperative biliary drainage
PC	plastic stents
PC-SEMS	partially covered SEMS
PeC	percutaneous cholecystostomy
PECMSs	paclitaxel-eluting covered metal stents
PEP	post-endoscopic retrograde cholangiopancreatography pancreatitis
PTBD	percutaneous transhepatic biliary drainage
PTCD	percutaneous transhepatic cholangial drainage
RCT	randomized controlled trials
RFA	radiofrequency ablation
ROB	recurrent biliary obstruction
ROB-2	the revised cochrane risk of bias tool
RR	relative risk
SEMS	self-expandable metal stent
SMD	standardized mean difference
SRoMAs	systematic reviews or meta-analyses
TRBO	time to recurrent biliary obstruction
U-SEMS	uncovered SEMS
WMD	weighted mean difference
