# Supplementary material for: Biliary drainage in malignant biliary obstruction: an umbrella review of randomized controlled trials
Source: Front Oncol. 2023 Sep 5;13:1235490. doi: 10.3389/fonc.2023.1235490 (PMC10508238; doi:10.3389/fonc.2023.1235490)
Supplement: Supplementary file 1 [file DataSheet_1.pdf]

Table S1-1: Embase Search Strategy

No.	Query	Results
#1	'biliary stent'/exp	5225
#2	'biliary tract drainage'/exp	26620
#3	'biliary drain'/exp	426
#4	't tube'/exp	2550
#5	'balloon dilatation'/exp	21768
#6	'nasobiliary drainage'/exp	33
#7	'nasobiliary tube'/exp	168
#8	'endoscopic nasobiliary drainage'/exp	105
#9	'endoscopic nasobiliary drainage tube'/exp	14
#10	'percutaneous transhepatic drainage'/exp	1966
#11	'percutaneous transhepatic cholangial drainage'/exp	26
#12	'percutaneous transhepatic biliary drainage'/exp	168
#13	'endoscopic sphincterotomy'/exp	5393
#14	'endoscopic papillary large balloon dilation'/exp	87
#15	'endoscopic papillary balloon dilation'/exp	74
#16	'endoscopic ultrasonography guided biliary drainage'/exp	10
#17	#1 OR #2 OR #3 OR #4 OR #5 OR #6 OR #7 OR #8 OR #9 OR #10 OR #11 OR #12 OR #13 OR #14 OR #15 OR #16	57004
#18	'biliary stent':ti,ab,kw OR 'biliary tract drainage':ti,ab,kw OR 'biliary drain':ti,ab,kw OR 't tube':ti,ab,kw OR 'balloon dilatatio':ti,ab,kw OR 'nasobiliary drainage':ti,ab,kw OR 'nasobiliary tube':ti,ab,kw OR 'endoscopic nasobiliary drainage':ti,ab,kw OR enbd:ti,ab,kw OR 'endoscopic nasobiliary drainage tube':ti,ab,kw OR ptcd:ti,ab,kw OR 'percutaneous transhepatic cholangial drainage':ti,ab,kw OR 'percutaneous transhepatic drainage':ti,ab,kw OR ptbd:ti,ab,kw OR 'percutaneous transhepatic biliary drainage':ti,ab,kw OR est:ti,ab,kw OR 'endoscopic sphincterotomy':ti,ab,kw OR eplbd:ti,ab,kw OR 'endoscopic papillary large balloon dilation':ti,ab,kw OR epbd:ti,ab,kw OR 'endoscopic papillary balloon dilation':ti,ab,kw OR 'eus bd':ti,ab,kw OR 'endoscopic ultrasonography guided biliary drainage':ti,ab,kw	32717
#19	#17 OR #18	79636
#20	'bile duct atresia'/exp	8520
#21	'common bile duct cyst'/exp	3683
#22	'bile duct dilatation'/exp	4338
#23	'acute cholecystitis'/exp	9081
#24	'chronic cholecystitis'/exp	2196
#25	'acalculous cholecystitis'/exp	1629
#26	'emphysematous cholecystitis'/exp	319
#27	'cholecystitis'/exp	30912
#28	'acute cholangitis'/exp	125
#29	'acute obstructive suppurative cholangitis'/exp	24
#30	'common bile duct stone'/exp	12359
#31	'cholelithiasis'/exp	66024

#32	'gallstone'/exp	19786
#33	'biliary pancreatitis'/exp	70
#34	'ascariasis'/exp	6702
#35	'bile duct cancer'/exp	38925
#36	'gallbladder cancer'/exp	13038
#37	'bile duct carcinoma'/exp	34417
#38	'distal cholangiocarcinoma'/exp	44
#39	'vater papilla carcinoma'/exp	2793
#40	'primary sclerosing cholangitis'/exp	10459
#41	'immunoglobulin g4 related disease'/exp	4359
#42	'patient history of liver transplantation'/exp	542
#43	'anastomotic stricture'/exp	289
#44	#20 OR #21 OR #22 OR #23 OR #24 OR #25 OR #26 OR #27 OR #28 OR #29 OR #30 OR #31 OR #32 OR #33 OR #34 OR #35 OR #36 OR #37 OR #38 OR #39 OR #40 OR #41 OR #42 OR #43	164940
#45	'biliary atresia':ti,ab,kw OR 'bile duct atresia':ti,ab,kw OR 'congenital choledochal cyst':ti,ab,kw OR 'common bile duct cyst':ti,ab,kw OR 'congenital intrahepatic duct dilatation':ti,ab,kw OR 'bile duct dilatation':ti,ab,kw OR 'acute cholecystitis':ti,ab,kw OR 'chronic cholecystitis':ti,ab,kw OR 'acalculous cholecystitis':ti,ab,kw OR 'emphysematous cholecystitis':ti,ab,kw OR cholecystitis:ti,ab,kw OR 'acute cholangitis':ti,ab,kw OR 'acute obstructive suppurative cholangitis':ti,ab,kw OR aosc:ti,ab,kw OR choledocholithiasis:ti,ab,kw OR 'common bile duct stone':ti,ab,kw OR 'intrahepatic bile duct stones':ti,ab,kw OR 'extrahepatic bile duct stones':ti,ab,kw OR 'bile duct stones':ti,ab,kw OR 'biliary stone':ti,ab,kw OR cholelithiasis:ti,ab,kw OR gallstone:ti,ab,kw OR cholecystolithiasis:ti,ab,kw OR 'biliary pancreatitis':ti,ab,kw OR 'biliary ascariasis':ti,ab,kw OR ascariasis:ti,ab,kw OR 'biliary cancer':ti,ab,kw OR 'biliary tumor':ti,ab,kw OR 'biliary neoplasms':ti,ab,kw OR 'biliary neoplasm':ti,ab,kw OR 'biliary mass':ti,ab,kw OR 'biliary masses':ti,ab,kw OR 'bile duct cancer':ti,ab,kw OR 'bile duct tumor':ti,ab,kw OR 'bile duct neoplasms':ti,ab,kw OR 'bile duct neoplasm':ti,ab,kw OR 'bile duct mass':ti,ab,kw OR 'bile duct masses':ti,ab,kw OR 'biliary duct cancer':ti,ab,kw OR 'biliary duct tumor':ti,ab,kw OR 'biliary duct neoplasms':ti,ab,kw OR 'biliary duct neoplasm':ti,ab,kw OR 'biliary duct mass':ti,ab,kw OR 'biliary duct masses':ti,ab,kw OR btc:ti,ab,kw OR 'gallbladder cancer':ti,ab,kw OR 'gallbladder carcinoma':ti,ab,kw OR 'gallbladder neoplasms':ti,ab,kw OR 'gallbladder neoplasm':ti,ab,kw OR 'gallbladder tumor':ti,ab,kw OR 'gallbladder mass':ti,ab,kw OR 'gallbladder masses':ti,ab,kw OR gbc:ti,ab,kw OR 'perihilar cholangiocarcinoma':ti,ab,kw OR 'hilar cholangiocarcinoma':ti,ab,kw OR 'hilar bile duct carcinoma':ti,ab,kw OR hcca:ti,ab,kw OR 'intrahepatic cholangiocarcinoma':ti,ab,kw OR icc:ti,ab,kw OR 'distal cholangiocarcinoma':ti,ab,kw OR dcca:ti,ab,kw OR 'carcinoma of ampulla':ti,ab,kw OR 'vater papilla carcinoma':ti,ab,kw OR 'ampulla cancer':ti,ab,kw OR 'ampullary carcinoma':ti,ab,kw OR 'ampullary cancer':ti,ab,kw OR vpc:ti,ab,kw OR 'primary sclerosing cholangitis':ti,ab,kw OR psc:ti,ab,kw OR 'igg4 cholangitis':ti,ab,kw OR 'immunoglobulin g4 related disease igg4':ti,ab,kw OR 'after liver	168287

	transplantation':ti,ab,kw OR 'patient history of liver transplantation':ti,ab,kw OR 'anastomotic stricture':ti,ab,kw OR 'anastomotic stricture of bile duct':ti,ab,kw OR 'benign biliary strictures':ti,ab,kw OR 'malignant biliary stricture':ti,ab,kw	
#46	#44 OR #45	250432
#47	'systematic review'/exp	353846
#48	'meta analysis'/exp	250581
#49	#47 OR #48	469777
#50	'meta analysis':ti,ab,kw OR 'meta analyse':ti,ab,kw OR meta:ti,ab,kw OR 'systematic review':ti,ab,kw OR 'systematic overview':ti,ab,kw	478970
#51	#49 OR #50	608979
#52	#19 AND #46 AND #51	520

Table S1-2: PubMed Search Strategy

Search number	Query	Results
1	EUS-BD OR endoscopic ultrasonography guided biliary drainage OR EPBD OR endoscopic papillary balloon dilation OR EPLBD OR endoscopic papillary large balloon dilation OR EST OR endoscopic sphincterotomy OR PTBD OR percutaneous transhepatic biliary drainage OR PTCD OR percutaneous transhepatic cholangial drainage OR percutaneous transhepatic drainage OR endoscopic nasobiliary drainage tube OR ENBD OR endoscopic nasobiliary drainage OR nasobiliary tube OR nasobiliary drainage OR balloon dilatatio OR t tube OR biliary drain OR biliary tract drainage OR biliary stent	65,960
2	biliary atresia OR bile duct atresia OR congenital choledochal cyst OR common bile duct cyst OR congenital intrahepatic duct dilatation OR bile duct dilatation OR acute cholecystitis OR chronic cholecystitis OR acalculous cholecystitis OR Emphysematous cholecystitis OR cholecystitis OR acute cholangitis OR acute obstructive suppurative cholangitis OR AOSC OR choledocholithiasis OR common bile duct stone OR intrahepatic bile duct stones OR extrahepatic bile duct stones OR bile duct stones OR Biliary stone OR cholelithiasis OR gallstone OR cholecystolithiasis OR biliary pancreatitis OR biliary ascariasis OR ascariasis OR biliary cancer OR biliary tumor OR biliary neoplasms OR biliary neoplasm OR biliary mass OR biliary masses OR bile duct cancer OR bile duct tumor OR bile duct neoplasms OR bile duct neoplasm OR bile duct mass OR bile duct masses OR biliary duct cancer OR biliary duct tumor OR biliary duct neoplasms OR biliary duct neoplasm OR biliary duct mass OR biliary duct masses OR BTC OR gallbladder cancer OR gallbladder carcinoma OR gallbladder neoplasms OR gallbladder neoplasm OR gallbladder tumor OR gallbladder mass OR gallbladder masses OR GBC OR perihilar cholangiocarcinoma OR hilar cholangiocarcinoma OR hilar bile duct carcinoma OR HCCA OR intrahepatic cholangiocarcinoma OR ICC OR distal cholangiocarcinoma OR DCCA OR vater papilla carcinoma OR ampulla cancer OR ampullary carcinoma OR ampullary cancer OR VPC OR primary sclerosing cholangitis OR PSC OR IgG4 cholangitis OR immunoglobulin G4 related disease OR after liver transplantation OR liver transplantation OR anastomotic stricture OR anastomotic biliary stricture OR benign biliary strictures OR malignant biliary stricture	323,442
3	meta OR meta analysis OR meta analyses OR systematic review OR systematic overview	443,403
4	((#1) AND (#2)) AND (#3)	464
5	(((biliary tract disease[MeSH Terms]) OR (bile[MeSH Terms])) AND (drainage[MeSH Terms])) AND (Systematic Review OR Meta-Analysis[MeSH Terms])	91
6	(#4) OR (#5)	485

Table S1-3: Web of science Search Strategy

Search number	Query	Results
#1	TS=(EUS-BD OR endoscopic ultrasonography guided biliary drainage OR EPBD OR endoscopic papillary balloon dilation OR EPLBD OR endoscopic papillary large balloon dilation OR EST OR endoscopic sphincterotomy OR PTBD OR percutaneous transhepatic biliary drainage OR PTCD OR percutaneous transhepatic cholangial drainage OR percutaneous transhepatic drainage OR endoscopic nasobiliary drainage tube OR ENBD OR endoscopic nasobiliary drainage OR nasobiliary tube OR nasobiliary drainage OR balloon dilatatio OR t tube OR biliary drain OR biliary tract drainage OR biliary stent)	59,224
#2	TS=(biliary atresia OR bile duct atresia OR congenital choledochal cyst OR common bile duct cyst OR congenital intrahepatic duct dilatation OR bile duct dilatation OR acute cholecystitis OR chronic cholecystitis OR acalculous cholecystitis OR Emphysematous cholecystitis OR cholecystitis OR acute cholangitis OR acute obstructive suppurative cholangitis OR AOSC OR choledocholithiasis OR common bile duct stone OR intrahepatic bile duct stones OR extrahepatic bile duct stones OR bile duct stones OR Biliary stone OR cholelithiasis OR gallstone OR cholecystolithiasis OR biliary pancreatitis OR biliary ascariasis OR ascariasis OR biliary cancer OR biliary tumor OR biliary neoplasms OR biliary neoplasm OR biliary mass OR biliary masses OR bile duct cancer OR bile duct tumor OR bile duct neoplasms OR bile duct neoplasm OR bile duct mass OR bile duct masses OR biliary duct cancer OR biliary duct tumor OR biliary duct neoplasms OR biliary duct neoplasm OR biliary duct mass OR biliary duct masses OR BTC OR gallbladder cancer OR gallbladder carcinoma OR gallbladder neoplasms OR gallbladder neoplasm OR gallbladder tumor OR gallbladder mass OR gallbladder masses OR GBC OR perihilar cholangiocarcinoma OR hilar cholangiocarcinoma OR hilar bile duct carcinoma OR HCCA OR intrahepatic cholangiocarcinoma OR ICC OR distal cholangiocarcinoma OR DCCA OR vater papilla carcinoma OR ampulla cancer OR ampullary carcinoma OR ampullary cancer OR VPC OR primary sclerosing cholangitis OR PSC OR IgG4 cholangitis OR immunoglobulin G4 related disease OR after liver transplantation OR liver transplantation OR anastomotic stricture OR anastomotic biliary stricture OR benign biliary strictures OR malignant biliary stricture)	267,163
#3	TS=(meta OR meta analysis OR meta analyses OR systematic review OR systematic overview)	596,162
#4	#1 AND #2 AND #3	384

Table S1-4: Cochrane Database Search Strategy

ID	Search	Hits
#1	(EUS-BD OR endoscopic ultrasonography guided biliary drainage OR EPBD OR endoscopic papillary balloon dilation OR EPLBD OR endoscopic papillary large balloon dilation OR EST OR endoscopic sphincterotomy OR PTBD OR percutaneous transhepatic biliary drainage OR PTCD OR percutaneous transhepatic cholangial drainage OR percutaneous transhepatic drainage OR endoscopic nasobiliary drainage tube OR ENBD OR endoscopic nasobiliary drainage OR nasobiliary tube OR nasobiliary drainage OR balloon dilatatio OR t tube OR biliary drain OR biliary tract drainage OR biliary stent):ti,ab,kw AND (biliary atresia OR bile duct atresia OR congenital choledochal cyst OR common bile duct cyst OR congenital intrahepatic duct dilatation OR bile duct dilatation OR acute cholecystitis OR chronic cholecystitis OR acalculous cholecystitis OR Emphysematous cholecystitis OR cholecystitis OR acute cholangitis OR acute obstructive suppurative cholangitis OR AOSC OR choledocholithiasis OR common bile duct stone OR intrahepatic bile duct stones OR extrahepatic bile duct stones OR bile duct stones OR Biliary stone OR cholelithiasis OR gallstone OR cholecystolithiasis OR biliary pancreatitis OR biliary ascariasis OR ascariasis OR biliary cancer OR biliary tumor OR biliary neoplasms OR biliary neoplasm OR biliary mass OR biliary masses OR bile duct cancer OR bile duct tumor OR bile duct neoplasms OR bile duct neoplasm OR bile duct mass OR bile duct masses OR biliary duct cancer OR biliary duct tumor OR biliary duct neoplasms OR biliary duct neoplasm OR biliary duct mass OR biliary duct masses OR BTC OR gallbladder cancer OR gallbladder carcinoma OR gallbladder neoplasms OR gallbladder neoplasm OR gallbladder tumor OR gallbladder mass OR gallbladder masses OR GBC OR perihilar cholangiocarcinoma OR hilar cholangiocarcinoma OR hilar bile duct carcinoma OR HCCA OR intrahepatic cholangiocarcinoma OR ICC OR distal cholangiocarcinoma OR DCCA OR vater papilla carcinoma OR ampulla cancer OR ampullary carcinoma OR ampullary cancer OR VPC OR primary sclerosing cholangitis OR PSC OR IgG4 cholangitis OR immunoglobulin G4 related disease OR after liver transplantation OR liver transplantation OR anastomotic stricture OR anastomotic biliary stricture OR benign biliary strictures OR malignant biliary stricture):ti,ab,kw AND (meta OR meta analysis OR meta analyses OR systematic review OR systematic overview):ti,ab,kw in Cochrane Reviews (Word variations have been searched)	122

Table S2. List of excluded studies and reasons for their exclusion

No.	Title	Reason for Exclusion
1	A comparison of balloon-versus stent-based approach for dominant strictures in primary sclerosing cholangitis: a meta-analysis	Not a systematic review or meta analysis of randomized controlled trials
2	A meta-analysis and systematic review: Comparing mortality and morbidity of endoscopic versus percutaneous approach in palliation of advanced hilar malignancies	Meeting abstract
3	A meta-analysis and systematic review: Success of endoscopic ultrasound guided biliary stenting in patients with inoperable malignant biliary strictures and a failed ERCP	Not a systematic review or meta analysis of randomized controlled trials
4	A meta-analysis of randomized trials: immediate stent placement vs. surgical bypass in the palliative management of malignant biliary obstruction	Topics related to surgical resection or surgical biliary reconstruction
5	A meta-analysis of the effect of preoperative biliary stenting on patients with obstructive jaundice	Not a systematic review or meta analysis of randomized controlled trials
6	A stent with radioactive seed strand insertion for inoperable malignant biliary obstruction: A meta-analysis	Not a systematic review or meta analysis of randomized controlled trials
7	A systematic review and meta-analysis of randomized trials and prospective studies comparing covered and bare self-expandable metal stents for the treatment of malignant obstruction in the digestive tract	Not a systematic review or meta analysis of randomized controlled trials
8	A systematic review of biodegradable biliary stents: Promising biocompatibility without stent removal	OR/HR/RR value were not calculated
9	A systematic review of the comparison of the incidence of seeding metastasis between endoscopic biliary drainage and percutaneous transhepatic biliary drainage for resectable malignant biliary obstruction	Not a systematic review or meta analysis of randomized controlled trials
10	A systematic review on efficacy and safety of partially and fully covered self-expandable metal stent (SEMS) compared to multiple plastic stents (MPS) in the endoscopic management of anastomotic biliary stricture post orthotopic liver transplantation (OLT)	Meeting abstract
11	Adverse events with lumen-apposing metal stents in endoscopic gallbladder drainage: A systematic review and meta-analysis	Not a systematic review or meta analysis of randomized controlled trials
12	Benign biliary strictures: A systematic review on endoscopic treatment options	OR/HR/RR value were not calculated
13	Best option for preoperative biliary drainage in Klatskin tumor: A systematic review and meta-analysis	Not a systematic review or meta analysis of randomized controlled trials
14	Bilateral stenting for hilar biliary obstruction: a meta-analysis of side-by-side versus stent-in-stent	Not a systematic review or meta analysis of randomized controlled trials
15	BILATERAL VERSUS UNILATERAL BILIARY DRAINAGE FOR MALIGNANT HILAR OBSTRUCTION: A SYSTEMIC REVIEW AND META-ANALYSIS	Meeting abstract
16	Bilateral versus unilateral stenting for malignant hilar obstruction: A meta-analysis	Meeting abstract
17	Bilateral vs unilateral placement of metal stents for inoperable high-grade hilar biliary strictures: A systemic review and meta-analysis	Not a systematic review or meta analysis of randomized controlled trials
18	Biliary diversion in progressive familial intrahepatic cholestasis: a systematic review and meta-analysis	Topics related to surgical resection or surgical biliary reconstruction
19	BILIARY DRAINAGE IN PATIENTS WITH CHOLANGIOCARCINOMA: A COMPARISON BETWEEN ENDOSCOPIC RETROGRADE CHOLANGIOPANCREATOGRAPHY AND PERCUTANEOUS TRANSHEPATIC APPROACHES	Meeting abstract
20	Biliary reconstruction after choledochal cyst resection: a systematic review and meta-analysis on hepaticojejunostomy vs hepaticoduodenostomy	Topics related to surgical resection or surgical biliary reconstruction
21	Biliary reconstruction, its complications and management of biliary complications after adult liver transplantation: A systematic review of the incidence, risk factors and outcome	OR/HR/RR value were not calculated
22	Biliary stent with or without I-125 seeds for malignant obstructive jaundice: a systematic review and meta-analysis	Not a systematic review or meta analysis of randomized controlled trials
23	Biliary stenting versus bypass surgery for the palliation of malignant distal bile duct obstruction: a meta-analysis	Topics related to surgical resection or surgical biliary reconstruction
24	Biliary tract reconstruction with or without T-tube in orthotopic liver transplantation: a systematic review and meta-analysis	Topics related to surgical resection or surgical biliary reconstruction

25	Biodegradable versus multiple plastic stent implantation in benign biliary strictures: A systematic review and meta-analysis	Not a systematic review or meta analysis of randomized controlled trials
26	Choledochoduodenostomy Versus Hepaticogastrostomy in Endoscopic Ultrasound-guided Drainage for Malignant Biliary Obstruction: A Meta-analysis and Systematic Review	Not a systematic review or meta analysis of randomized controlled trials
27	Clinical outcome of endoscopic covered metal stenting for resolution of benign biliary stricture: Systematic review and meta-analysis	Not a systematic review or meta analysis of randomized controlled trials
28	Clinical outcomes of biliary drainage of malignant biliary obstruction due to colorectal cancer metastases: A systematic review	OR/HR/RR value were not calculated
29	Clinical outcomes, and long-term stent patency in patients with malignant biliary obstruction: A systematic review and meta-analysis	Meeting abstract
30	Comparative Effectiveness of Metal Versus Plastic Stents for Preoperative Biliary Drainage in Resectable and Borderline Resectable Distal Malignant Biliary Obstruction: a Systematic Review and Meta-Analysis	Meeting abstract
31	Comparative efficacy of various stents for palliation in patients with malignant extrahepatic biliary obstruction: A systematic review and network meta-analysis	Not a systematic review or meta analysis of randomized controlled trials
32	Comparing endoscopic ultrasound guided versus percutaneous biliary stenting in patients with inoperable malignant biliary strictures and a failed ERCP: A systematic review and meta-analysis	Meeting abstract
33	Comparing Outcomes Following Endoscopic Ultrasound-Guided Biliary Drainage Versus Percutaneous Transhepatic Biliary Drainage for Malignant Biliary Obstruction: A Systematic Review and Meta-Analysis	Not a systematic review or meta analysis of randomized controlled trials
34	Comparing outcomes of Roux-En-Y choledochojejunostomy versus duct-to-duct biliary anastomosis in liver transplantation in primary sclerosing cholangitis: A meta-analysis	Topics related to surgical resection or surgical biliary reconstruction
35	Comparison of Biliary Drainage Techniques for Malignant Biliary Obstruction: A Systematic Review and Network Meta-analysis	Not a systematic review or meta analysis of randomized controlled trials
36	Comparison of effect between nasobiliary drainage and biliary stenting in malignant biliary obstruction: a systematic review and updated meta-analysis	Not a systematic review or meta analysis of randomized controlled trials
37	Comparison of Effects of iodine-125 seeds stent and Common Biliary Stent in Palliative Treatment of Patients with Unresectable Cholangiocarcinoma: A Systematic Review and Meta-analysis	Unable to get full text
38	COMPARISON OF ENDOSCOPIC ULTRASOUND GUIDED BILIARY STENTING, PERCUTANEOUS TRANSHEPATIC BILIARY DRAINAGE, AND ENDOSCOPIC RETROGRADE CHOLANGIOPANCREATOGRAPHY IN PATIENTS WITH MALIGNANT BILIARY OBSTRUCTION: A SYSTEMATIC REVIEW AND META-ANALYSIS	Meeting abstract
39	Comparison of EUS Guided Biliary Drainage With Percutaneous Biliary Drainage: Updated Meta-Analysis	Meeting abstract
40	Comparison of EUS-guided endoscopic transpapillary and percutaneous gallbladder drainage for acute cholecystitis: a systematic review with network meta-analysis	Not a systematic review or meta analysis of randomized controlled trials
41	Comparison of Intraductal RFA Plus Stent versus Stent-Only Treatment for Unresectable Perihilar Cholangiocarcinoma-A Systematic Review and Meta-Analysis	Not a systematic review or meta analysis of randomized controlled trials
42	Comparison of liver transplantation outcomes in biliary atresia patients with and without prior portoenterostomy: A meta-analysis	Topics related to surgical resection or surgical biliary reconstruction
43	Comparison of long-term efficacy between endoscopic and percutaneous biliary drainage for resectable extrahepatic cholangiocarcinoma with biliary obstruction: A systematic review and meta-analysis	Not a systematic review or meta analysis of randomized controlled trials
44	Comparison of Metal and Plastic Stents for Preoperative Biliary Drainage in Resectable and Borderline Resectable Periampullary Cancer: A Meta-Analysis and System Review	Not a systematic review or meta analysis of randomized controlled trials
45	Comparison of Three Methods of Gallbladder Drainage for Patients with Acute Cholecystitis Who Are at High Surgical Risk: A Network Meta-Analysis and Systematic Review	Not a systematic review or meta analysis of randomized controlled trials
46	CONVERSION OF PERCUTANEOUS CHOLECYSTOSTOMY TO ENDOSCOPIC ULTRASOUND GUIDED GALL BLADDER DRAINAGE: SYSTEMATIC REVIEW AND META-ANALYSIS	Meeting abstract
47	Covered and Uncovered Self-Expandable Metallic Stents in the Treatment of Malignant Biliary Obstruction	Unable to get full text
48	COVERED SELF-EXPANDABLE METALLIC STENT VERSUS PLASTIC STENTS FOR ANASTOMOTIC BILIARY STRICTURES AFTER LIVER TRANSPLANTATION: A SYSTEMATIC REVIEW OF RANDOMIZED CONTROLLED TRIALS	Meeting abstract
49	Covered self-expanding metal stents may be preferable to plastic stents in the treatment of chronic pancreatitis-related biliary strictures: A systematic review comparing 2 methods of stent therapy in benign biliary strictures	OR/HR/RR value were not calculated

50	Double Stenting for Malignant Biliary and Duodenal Obstruction: A Systematic Review and Meta-Analysis	Not a systematic review or meta analysis of randomized controlled trials
51	Drug Eluting Versus Covered Metal Stents in Malignant Biliary Strictures-Is There a Clinical Benefit? A Systematic Review and Meta-Analysis	Not a systematic review or meta analysis of randomized controlled trials
52	Effect of Preoperative Biliary Drainage on Length of Post-Operative Hospital Stay in Patient With Malignant Obstructive Jaundice: A Meta-Analysis and Systematic Review	Meeting abstract
53	Effect of preoperative biliary drainage on malignant obstructive jaundice: a meta-analysis	Not a systematic review or meta analysis of randomized controlled trials
54	Effectiveness and safety of EUS-guided choledochoduodenostomy using lumen-apposing metal stents (LAMS): a systematic review and meta-analysis	Not a systematic review or meta analysis of randomized controlled trials
55	Effects of different preoperative biliary drainage methods for resected malignant obstruction jaundice on the incidence rate of implantation metastasis: A meta-analysis	Not a systematic review or meta analysis of randomized controlled trials
56	Efficacy and complications of nasobiliary tube placement in patients with bile leak after liver transplantation: A systematic review	Meeting abstract
57	Efficacy and safety of endoscopic gallbladder drainage in acute cholecystitis: Is it better than percutaneous gallbladder drainage?	Not a systematic review or meta analysis of randomized controlled trials
58	Efficacy and safety of endoscopic transpapillary gallbladder drainage in acute cholecystitis: An updated meta-analysis	Not a systematic review or meta analysis of randomized controlled trials
59	Efficacy and Safety of Endoscopic Ultrasound (EUS)-Guided Choledochoduodenostomy (CDD): A Systematic Review and Meta-Analysis	Not a systematic review or meta analysis of randomized controlled trials
60	Efficacy and Safety of Endoscopic Ultrasound-Guided Biliary Drainage: A Systematic Review and Meta-analysis	Not a systematic review or meta analysis of randomized controlled trials
61	Efficacy and safety of lumen apposing self-expandable metal stents for EUS guided cholecystostomy: A meta-analysis and systematic review	Not a systematic review or meta analysis of randomized controlled trials
62	Efficacy and safety of preoperative biliary drainage in patients undergoing pancreaticoduodenectomy: an updated systematic review and meta-analysis	Not a systematic review or meta analysis of randomized controlled trials
63	Efficacy of Different Endoscopic Stents in the Management of Postoperative Biliary Strictures A Systematic Review and Meta-analysis	Not a systematic review or meta analysis of randomized controlled trials
64	Efficacy of endoscopic biliary stents in patients with bile leak after liver transplantation: A systematic review	OR/HR/RR value were not calculated
65	Efficacy of preoperative biliary drainage in malignant obstructive jaundice: A meta-analysis and systematic review	Not a systematic review or meta analysis of randomized controlled trials
66	ENDOSCOPIC BALLOON DILATION VERSUS ENDOSCOPIC BALLOON DILATION WITH STENTING FOR DOMINANT STRICTURES IN PRIMARY SCLEROSING CHOLANGITIS: A COMPARATIVE META-ANALYSIS	Meeting abstract
67	ENDOSCOPIC BILATERAL DRAINAGE TECHNIQUES OF MALIGNANT HILAR BILIARY OBSTRUCTION: STENT-IN-STENT OR SIDE-BY-SIDE? A SYSTEMATIC REVIEW AND META-ANALYSIS	Meeting abstract
68	Endoscopic Bilateral Stent-in-Stent Versus Stent-by-Stent Deployment in Advanced Malignant Hilar Obstruction: A Meta-Analysis and Systematic Review	Not a systematic review or meta analysis of randomized controlled trials
69	Endoscopic Biliary Drainage Versus Percutaneous Transhepatic Biliary Drainage in Patients with Resectable Hilar Cholangiocarcinoma: A Systematic Review and Meta-Analysis	Not a systematic review or meta analysis of randomized controlled trials
70	Endoscopic biliary self-expandable metallic stent in malignant biliary obstruction with or without sphincterotomy: systematic review and meta-analysis	Not a systematic review or meta analysis of randomized controlled trials
71	Endoscopic biliary stenting in irretrievable common bile duct stones: stent exchange or expectant management-tertiary-centre experience and systematic review	OR/HR/RR value were not calculated
72	Endoscopic gallbladder drainage for management of acute cholecystitis	OR/HR/RR value were not calculated
73	Endoscopic gallbladder drainage for symptomatic gallbladder disease: a cumulative systematic review meta-analysis	Not a systematic review or meta analysis of randomized controlled trials
74	Endoscopic metal stenting for malignant hilar biliary obstruction: an update meta-analysis of unilateral versus bilateral stenting	Not a systematic review or meta analysis of randomized controlled trials

75	Endoscopic papillary balloon dilation vs endoscopic sphincterotomy: An update meta-analysis	Meeting abstract
76	ENDOSCOPIC SPHINCTEROTOMY VS ENDOSCOPIC PAPPILLARY LARGE BALLOON DILATION VS ENDOSCOPIC SPHINCTEROTOMY PLUS LARGE BALLOON DILATION FOR LARGE COMMON BILE DUCT STONE: A COMPARATIVE NETWORK META-ANALYSIS OF RANDOMIZED CONTROLLED TRIALS	Meeting abstract
77	Endoscopic stenting for malignant hilar biliary obstruction: should it be metal or plastic and unilateral or bilateral?	Not a systematic review or meta analysis of randomized controlled trials
78	Endoscopic treatment of benign biliary stricture using different stents: a systematic review and meta-analysis	Not a systematic review or meta analysis of randomized controlled trials
79	Endoscopic treatment of post-liver transplantation anastomotic biliary stricture: Systematic review and meta-analysis	Not a systematic review or meta analysis of randomized controlled trials
80	Endoscopic Ultrasound Guided Biliary Drainage Comes of Age: a Systematic Review and Meta-Analysis	Meeting abstract
81	Endoscopic ultrasound vs. endoscopic trans-papillary vs. percutaneous gallbladder drainage in high-risk acute cholecystitis patients: A systematic review and indirect comparative meta-analysis	Meeting abstract
82	Endoscopic ultrasound-guided biliary drainage versus percutaneous transhepatic biliary drainage after failed endoscopic retrograde cholangiopancreatography: a meta-analysis	Not a systematic review or meta analysis of randomized controlled trials
83	ENDOSCOPIC ULTRASOUND-GUIDED BILIARY DRAINAGE(EUS-BD)VS. ERCP IN MALIGNANT BILIARY OBSTRUCTION, DUODENAL STENOSIS AND ABNORMAL SURGICAL ANATOMY: A SYSTEMATIC REVIEW AND META ANALYSIS	Meeting abstract
84	Endoscopic Ultrasound-Guided Biliary Drainage: A Systematic Review and Meta-Analysis	Not a systematic review or meta analysis of randomized controlled trials
85	ENDOSCOPIC ULTRASOUND-GUIDED CHOLECYSTOSTOMY VERSUS PERCUTANEOUS CHOLECYSTOSTOMY IN THE TREATMENT OF ACUTE CHOLECYSTITIS: A SYSTEMATIC REVIEW AND META- ANALYSIS	Meeting abstract
86	Endoscopic ultrasound-guided gallbladder drainage for acute cholecystitis with a lumen-apposing metal stent: A systematic review of case series	OR/HR/RR value were not calculated
87	Endoscopic ultrasound-guided gallbladder drainage is better than percutaneous cholecystotomy in high-risk patients with acute cholecystitis: A meta-analysis	Meeting abstract
88	Endoscopic Ultrasound-Guided Gallbladder Drainage Using a Lumen-Apposing Metal Stent for Acute Cholecystitis: A Systematic Review	OR/HR/RR value were not calculated
89	Endoscopic ultrasound-guided gallbladder drainage versus percutaneous cholecystostomy for high risk surgical patients with acute cholecystitis: A systematic review and meta-Analysis	Not a systematic review or meta analysis of randomized controlled trials
90	Endoscopic Ultrasound-Guided Gallbladder Drainage Versus Percutaneous Transhepatic Gallbladder Drainage for Acute Cholecystitis with High Surgical Risk: An Up-to-Date Meta-Analysis and Systematic Review	Not a systematic review or meta analysis of randomized controlled trials
91	Endoscopic ultrasound-guided gallbladder drainage, transpapillary drainage, or percutaneous drainage in high risk acute cholecystitis patients: A systematic review and comparative meta-analysis	Not a systematic review or meta analysis of randomized controlled trials
92	Endoscopic ultrasound-guided transmural approach versus ERCP-guided transpapillary approach for primary decompression of malignant biliary obstruction: A meta-analysis	Not a systematic review or meta analysis of randomized controlled trials
93	Endoscopic ultrasound-guided transmural by gastroenterologist vs percutaneous transhepatic by interventional radiologist for gallbladder drainage in acute cholecystitis in high-risk surgical patients	Meeting abstract
94	ENDOSCOPIC ULTRASOUND-GUIDED TRANSMURAL GALLBLADDER DRAINAGE (EUS-GBD) VERSUS PERCUTANEOUS TRANSHEPATIC GALLBLADDER DRAINAGE (PT-GBD) IN HIGH-RISK PATIENTS WITH ACUTE CHOLECYSTITIS - A SYSTEMATIC REVIEW AND META-ANALYSIS	Meeting abstract
95	Endoscopic ultrasound-guided versus endoscopic retrograde cholangiopancreatography-guided biliary drainage for primary treatment of distal malignant biliary obstruction: A systematic review and meta-analysis	Not a systematic review or meta analysis of randomized controlled trials
96	Endoscopic ultrasound-guided vs ERCP-guided biliary drainage for malignant biliary obstruction: A up-to-date meta-analysis and systematic review	Not a systematic review or meta analysis of randomized controlled trials
97	Endoscopic versus Percutaneous Biliary Drainage in Palliation of Advanced Malignant Hilar Obstruction: A Meta-Analysis and Systematic Review	Not a systematic review or meta analysis of randomized controlled trials
98	Endoscopic versus percutaneous preoperative biliary drainage in patients with Klatskin tumour undergoing curative surgery: A systematic review and meta-analysis of short-term and long-term outcomes	Not a systematic review or meta analysis of randomized controlled trials
99	Endoscopic vs percutaneous approach for successful biliary drainage in palliation of advanced hilar malignancies: A meta-analysis and systematic review	Meeting abstract

100	Endoscopic vs percutaneous preoperative biliary drainage in hilar cholangiocarcinoma: A systematic review and meta-analysis	Meeting abstract
101	EUS Guided Biliary Drainage After Failed ERCP: a Meta Analysis of the Technical Success and Adverse Events	Meeting abstract
102	EUS GUIDED VERSUS ERCP GUIDED BILIARY DRAINAGE FOR MALIGNANT BILIARY OBSTRUCTION: META-ANALYSIS OF RANDOMIZED CONTROLLED TRIALS	Meeting abstract
103	EUS GUIDED VERSUS ERCP GUIDED BILIARY DRAINAGE FOR PRIMARY PALLIATION OF MALIGNANT BILIARY STRICTURES: A SYSTEMATIC REVIEW AND META-ANALYSIS	Meeting abstract
104	EUS VERSUS ERCP FOR PRIMARY DRAINAGE OF MALIGNANT BILIARY OBSTRUCTION: A SYSTEMATIC REVIEW AND META-ANALYSIS BASED ON RANDOMIZED TRIALS	Meeting abstract
105	EUS-GUIDED BILIARY DRAINAGE FOR FIRST AND SECOND-LINE DECOMPRESSION OF MALIGNANT BILIARY OBSTRUCTION: A SYSTEMATIC REVIEW AND META-ANALYSIS OF RANDOMIZED CONTROLLED TRIALS	Meeting abstract
106	EUS-guided biliary drainage is equivalent to ERCP for primary treatment of malignant distal biliary obstruction: A systematic review and meta-analysis	Not a systematic review or meta analysis of randomized controlled trials
107	EUS-GUIDED BILIARY DRAINAGE IS NON-INFERIOR TO ERCP FOR FIRST-LINE PALLIATION OF MALIGNANT DISTAL BILIARY	Meeting abstract
108	EUS-Guided Biliary Drainage Is Superior to Percutaneous Biliary Drainage: A Systematic Review and Meta-Analysis	Meeting abstract
109	EUS-guided biliary drainage: A systematic review and meta-analysis	Not a systematic review or meta analysis of randomized controlled trials
110	EUS-guided Choledochoduodenostomy Versus Hepaticogastrostomy A Systematic Review and Meta-analysis	Not a systematic review or meta analysis of randomized controlled trials
111	Eus-guided gallbladder drainage versus endoscopic trans-papillary gallbladder drainage for high risk surgical patients with acute cholecystitis: A systematic review and meta-analysis	Meeting abstract
112	EUS-guided versus endoscopic transpapillary gallbladder drainage in high-risk surgical patients with acute cholecystitis: a systematic review and meta-analysis	Not a systematic review or meta analysis of randomized controlled trials
113	EUS-guided versus percutaneous transhepatic cholangiography biliary drainage for obstructed distal malignant biliary strictures in patients who have failed endoscopic retrograde cholangiopancreatography: A systematic review and meta-analysis	Not a systematic review or meta analysis of randomized controlled trials
114	EUS-guided versus percutaneous transhepatic gallbladder drainage in patients with acute cholecystitis who are not eligible for surgery- a systematic review and meta-analysis	Meeting abstract
115	EUS-guided vs percutaneous drainage for acute cholecystitis in high-risk patients: A systematic review and meta-analysis	Meeting abstract
116	Fully covered self-expandable metal stents for treatment of benign biliary strictures	Unable to get full text
117	How to Choose Between Percutaneous Transhepatic and Endoscopic Biliary Drainage in Malignant Obstructive Jaundice: An Updated Systematic Review and Meta-analysis	Not a systematic review or meta analysis of randomized controlled trials
118	ID: 3520449 ENDOSCOPIC ULTRASOUND-GUIDED TRANSMURAL GALLBLADDER DRAINAGE (EUS-GBD) VERSUS PERCUTANEOUS TRANSHEPATIC GALLBLADDER DRAINAGE (PT-GBD) IN HIGH-RISK PATIENTS WITH ACUTE CHOLECYSTITIS - A SYSTEMATIC REVIEW AND META-ANALYSIS	Meeting abstract
119	ID: 3523801 THE SAFETY AND EFFICACY OF BALLOON DILATION COMPARED WITH STENT PLACEMENT FOR THE MANAGEMENT OF DOMINANT STRICTURE IN PRIMARY SCLEROSING CHOLANGITIS: A SYSTEMATIC REVIEW AND META-ANALYSIS	Meeting abstract
120	ID: 3524593 ENDOSCOPIC BALLOON DILATION VERSUS ENDOSCOPIC BALLOON DILATION WITH STENTING FOR DOMINANT STRICTURES IN PRIMARY SCLEROSING CHOLANGITIS: A COMPARATIVE META-ANALYSIS	Meeting abstract
121	ID: 3527052 ENDOSCOPIC ULTRASOUND-GUIDED GALLBLADDER DRAINAGE VERSUS PERCUTANEOUS TRANSHEPATIC CHOLECYSTOSTOMY FOR ACUTE CHOLECYSTITIS: A SYSTEMATIC REVIEW AND META-ANALYSIS	Meeting abstract
122	Internal drainage versus external drainage in palliation of malignant biliary obstruction: a meta-analysis and systematic review	Not a systematic review or meta analysis of randomized controlled trials
123	IS IT TIME FOR EUS-GUIDED GALLBLADDER DRAINAGE TO GO MAINSTREAM? AN UPDATED META-ANALYSIS	Meeting abstract
124	Is percutaneous approach better than endoscopic approach for successful biliary drainage in palliation of unresectable hilar cholangiocarcinoma: A meta-analysis and systematic review	Meeting abstract
125	Is Percutaneous Transhepatic Biliary Drainage Better than Endoscopic Drainage in the Management of Jaundiced Patients Awaiting Pancreaticoduodenectomy?	Not a systematic review or meta analysis

	A Systematic Review and Meta-analysis	of randomized controlled trials
126	Is safe and efficient by intraoperative endoscopic nasobiliary drainage over primary closure of the common bile duct for cholecystolithiasis combined with common bile duct stones: A meta-analysis	Meeting abstract
127	Laparoscopic portoenterostomy versus open portoenterostomy for the treatment of biliary atresia: a systematic review and meta-analysis of comparative studies	Topics related to surgical resection or surgical biliary reconstruction
128	Laparoscopic vs open portoenterostomy in biliary atresia: a systematic review and meta-analysis	Topics related to surgical resection or surgical biliary reconstruction
129	Lumen Apposing Metal Stents (LAMSs) for Drainage of Pancreatic and Gallbladder Collections: A Meta-analysis	Not a systematic review or meta analysis of randomized controlled trials
130	Malignant distal biliary obstruction: A systematic review and meta-analysis of endoscopic and surgical bypass results	Topics related to surgical resection or surgical biliary reconstruction
131	Management of occluded metal stents in malignant biliary obstruction: Similar outcomes with second metal stents compared to plastic stents	Not a systematic review or meta analysis of randomized controlled trials
132	Management of occluded self-expandable metallic stents in patients with malignant biliary obstruction: A systematic review and meta-analysis	Meeting abstract
133	Managing the post-liver transplantation anastomotic biliary stricture: multiple plastic versus metal stents: a systematic review	OR/HR/RR value were not calculated
134	Meta-analysis of Duct-to-duct versus Roux-en-Y biliary reconstruction following liver transplantation for primary sclerosing cholangitis	Topics related to surgical resection or surgical biliary reconstruction
135	Meta-analysis of outcomes of endoscopic ultrasound-guided gallbladder drainage versus percutaneous cholecystostomy for the management of acute cholecystitis	Not a systematic review or meta analysis of randomized controlled trials
136	Metaanalysis of randomized controlled trials of endoscopic retrograde cholangiography and endoscopic sphincterotomy for the treatment of acute biliary pancreatitis	Meeting abstract
137	Meta-analysis of randomized, controlled trials of endoscopic retrograde cholangiography (ERC) and endoscopic sphincterotomy (ES) in acute pancreatitis due to gallstones.	Meeting abstract
138	Meta-analysis of the efficacy of preoperative biliary drainage in hilar cholangiocarcinoma	Not a systematic review or meta analysis of randomized controlled trials
139	Meta-analysis of the efficacy of preoperative biliary drainage in patients undergoing liver resection for perihilar cholangiocarcinoma	Not a systematic review or meta analysis of randomized controlled trials
140	Meta-analysis of the long term success rate of different interventions in benign biliary strictures	Not a systematic review or meta analysis of randomized controlled trials
141	Meta-analysis on the endoscopic treatment of liver transplantation biliary stricture	Meeting abstract
142	METALLIC STENT VS. MULTIPLE PLASTIC STENTS FOR THE ANASTOMOTIC BILIARY STRICTURE AFTER LIVER TRANSPLANTATION. SYSTEMATIC REVIEW AND META-ANALYSIS OF RANDOMIZED CONTROLLED TRIALS	Meeting abstract
143	Methods for Drainage of Distal Malignant Biliary Obstruction after ERCP Failure: A Systematic Review and Network Meta-Analysis	Topics related to surgical resection or surgical biliary reconstruction
144	Mortality and morbidity with endoscopic versus percutaneous biliary drainage in palliation of unresectable hilar cholangiocarcinoma: A meta-analysis and systematic review	Meeting abstract
145	No benefit of covered vs uncovered self-expandable metal stents in patients with malignant distal biliary obstruction: a meta-analysis	Not a systematic review or meta analysis of randomized controlled trials
146	NO BENEFIT OF PERFORMING ENDOSCOPIC SPHINCTEROTOMY BEFORE STENT PLACEMENT IN PATIENTS WITH DISTAL MALIGNANT BILIARY STRICTURES: A META-ANALYSIS OF RCTS	Meeting abstract
147	Non-surgical percutaneous gallbladder drainage for the treatment of acute cholecystitis: Percutaneous gallbladder aspiration: A systematic review	Meeting abstract
148	Orthotopic liver transplantation: T-tube or not T-tube? Systematic review and meta-analysis of results	Topics related to surgical resection or surgical biliary reconstruction
149	Outcomes of endoscopic ultrasound-guided biliary drainage: A systematic review and meta-analysis	Not a systematic review or meta analysis of randomized controlled trials
150	Outcomes of laparoscopic Kasai portoenterostomy for biliary atresia: A systematic review	OR/HR/RR value were not calculated

151	Palliative Endoscopic and Percutaneous Biliary Radiofrequency Ablation with Stenting Compared to Biliary Stenting Alone in the Treatment of Unresectable Cholangiocarcinoma: A Meta-Analysis on Survival Outcomes	Meeting abstract
152	Palliative Endoscopic Biliary Radiofrequency Ablation with Stenting Compared to Percutaneous Biliary Radiofrequency Ablation with Stenting: A Meta-Analysis on Survival Outcomes	Meeting abstract
153	Palliative Percutaneous Biliary Radiofrequency Ablation with Stenting Compared to Biliary Stenting Alone in the Treatment of Unresectable Cholangiocarcinoma: A Meta-Analysis on Survival Outcomes	Meeting abstract
154	Percutaneous metal stenting for malignant hilar biliary obstruction: a systematic review and meta-analysis of unilateral versus bilateral stenting	Not a systematic review or meta analysis of randomized controlled trials
155	Percutaneous transhepatic biliary drainage vs. Endoscopic biliary drainage in periampullary cancer patients undergoing pancreaticoduodenectomy-a systematic review and meta-analysis	Not a systematic review or meta analysis of randomized controlled trials
156	Percutaneous transhepatic gallbladder drainage combined with laparoscopic cholecystectomy: A meta-Analysis of randomized controlled trials	Topics related to surgical resection or surgical biliary reconstruction
157	Percutaneous vs. endoscopic pre-operative biliary drainage in hilar cholangiocarcinoma - A systematic review and meta-analysis	Not a systematic review or meta analysis of randomized controlled trials
158	Plastic or metal stents for benign extrahepatic biliary strictures: a systematic review	OR/HR/RR value were not calculated
159	Preoperative biliary drainage before resection for hilar cholangiocarcinoma: Whether or not? A systematic review	Not a systematic review or meta analysis of randomized controlled trials
160	Preoperative biliary drainage in hilar cholangiocarcinoma: Systematic review and meta-analysis	Not a systematic review or meta analysis of randomized controlled trials
161	Preoperative Biliary Drainage in Patients with Resectable Perihilar Cholangiocarcinoma: Is Percutaneous Transhepatic Biliary Drainage Safer and More Effective than Endoscopic Biliary Drainage? A Meta-Analysis	Not a systematic review or meta analysis of randomized controlled trials
162	Pre-operative biliary drainage in perihilar cholangiocarcinoma: an overview of systematic reviews	Meeting abstract
163	Preoperative biliary drainage in resectable pancreatic cancer: a systematic review and network meta-analysis	Not a systematic review or meta analysis of randomized controlled trials
164	PRE-OPERATIVE BILIARY DRAINAGE IN THE PERIAMPULLARY NEOPLASIA - A SYSTEMATIC REVIEW	Meeting abstract
165	Preoperative endoscopic stent placement before pancreaticoduodenectomy: a meta-analysis of the effect on morbidity and mortality	Not a systematic review or meta analysis of randomized controlled trials
166	PRIMARY CLOSURE OF CBD WITH ANTEGRADE STENTING FOR CHOLEDOCHOLITHIASIS	Meeting abstract
167	Primary EUS-guided biliary drainage versus ERCP drainage for the management of malignant biliary obstruction: A systematic review and meta-analysis	Not a systematic review or meta analysis of randomized controlled trials
168	Primary suture versus T-tube drainage after laparoscopic common bile duct stone exploration: A systematic review	Meeting abstract
169	Radiofrequency ablation combined with biliary stent placement versus stent placement alone for malignant biliary strictures: a systematic review and meta-analysis	Not a systematic review or meta analysis of randomized controlled trials
170	Radiofrequency ablation combined with biliary stenting versus stenting alone for malignant biliary strictures-a systematic review and meta-analysis	Meeting abstract
171	Relationship of postoperative complications from preoperative biliary stents after pancreaticoduodenectomy. A new cohort analysis and meta-analysis of modern studies	Not a systematic review or meta analysis of randomized controlled trials
172	Relief of jaundice in malignant biliary obstruction: When should we consider endoscopic ultrasonography-guided hepaticogastrostomy as an option?	OR/HR/RR value were not calculated
173	Risk of Post-ERCP Pancreatitis With the Placement of Covered Versus Uncovered Self-Expandable Biliary Metal Stents: Systematic Review and Meta-Analysis	Meeting abstract
174	Roux-en-Y choledochojejunostomy versus duct-to-duct biliary anastomosis in liver transplantation for primary sclerosing cholangitis: A meta-analysis	Topics related to surgical resection or surgical biliary reconstruction
175	SAFETY AND EFFICACY OF ENDOSCOPIC TRANSPAPILLARY GALLBLADDER STENTING FOR SYMPTOMATIC GALLBLADDER DISEASE IN CIRRHOSIS: A SYSTEMATIC REVIEW AND META-ANALYSIS	Meeting abstract
176	Safety and Efficacy of Trans-Luminal Biliary Drainage Using the Choledochduodenostomy vs Hepaticogastrostomy After Failed ERCP. Systemic Review and Meta-Analysis	Meeting abstract

177	Side-by-side versus stent-in-stent bilateral stenting for malignant hilar biliary obstruction: a meta-analysis	Not a systematic review or meta analysis of randomized controlled trials
178	Sphincterotomy with endoscopic biliary drainage for severe acute cholangitis: a meta-analysis	Not a systematic review or meta analysis of randomized controlled trials
179	Stent insertion for hilar cholangiocarcinoma: a meta-analysis of comparison between unilateral and bilateral stenting	Not a systematic review or meta analysis of randomized controlled trials
180	STENT VERSUS BALLOON DILATION FOR THE TREATMENT OF DOMINANT STRICTURES IN PRIMARY SCLEROSING CHOLANGITIS: A SYSTEMATIC REVIEW AND META-ANALYSIS	Meeting abstract
181	Stenting with high-intensity focused ultrasound ablation for distal biliary obstruction caused by pancreatic carcinoma: a meta-analysis	Not a systematic review or meta analysis of randomized controlled trials
182	Systematic appraisal of the role of metallic endobiliary stents in the treatment of benign bile duct stricture	OR/HR/RR value were not calculated
183	Systematic review and meta-analysis of biliary reconstruction techniques in orthotopic deceased donor liver transplantation	Topics related to surgical resection or surgical biliary reconstruction
184	Systematic review and meta-analysis of metal versus plastic stents for preoperative biliary drainage in resectable periampullary or pancreatic head tumors	Not a systematic review or meta analysis of randomized controlled trials
185	SYSTEMATIC REVIEW AND META-ANALYSIS OF STUDIES COMPARING METAL OR PLASTIC STENTS FOR PREOPERATIVE BILIARY DRAINAGE IN RESECTABLE PANCREATIC CANCER	Meeting abstract
186	Systematic Review and Meta-analysis: Partial External Biliary Diversion in Progressive Familial Intrahepatic Cholestasis	OR/HR/RR value were not calculated
187	Systematic review with meta-analysis of studies comparing primary duct closure and T-tube drainage after laparoscopic common bile duct exploration for choledocholithiasis	Not a systematic review or meta analysis of randomized controlled trials
188	T - Tube Biliary Reconstruction in Liver Transplantation: Useful or Harmful? A Meta Analysis.	Topics related to surgical resection or surgical biliary reconstruction
189	The effect and safety of preoperative biliary drainage in patients with hilar cholangiocarcinoma: An updated meta-analysis	Not a systematic review or meta analysis of randomized controlled trials
190	The effect of preoperative biliary stents on outcomes after pancreaticoduodenectomy: A meta-analysis	Not a systematic review or meta analysis of randomized controlled trials
191	The Efficacy and Safety of Endoscopic Balloon Dilation Combined with Stenting in Patients with Biliary Anastomotic Strictures After Orthotopic Liver Transplantation	Not a systematic review or meta analysis of randomized controlled trials
192	THE EFFICACY AND SAFETY OF ENDOSCOPIC ULTRASOUND GUIDED BILIARY DRAINAGE IN BENIGN BILIARY DISEASE IN PATIENTS WITH SURGICALLY ALTERED ANATOMY	Meeting abstract
193	The efficacy and safety of fully covered self expandible metal biliary stent for the treatment of post ERCP sphincterotomy bleeding: A systematic review and meta-analysis	Meeting abstract
194	THE EFFICACY AND SAFETY OF PREOPERATIVE BILIARY DRAINAGE IN PATIENTS WITH OBSTRUCTIVE JAUNDICE: A SYSTEMATIC REVIEW AND META-ANALYSIS	Meeting abstract
195	THE SAFETY AND EFFICACY OF BALLOON DILATION COMPARED WITH STENT PLACEMENT FOR THE MANAGEMENT OF DOMINANT STRICTURE IN PRIMARY SCLEROSING CHOLANGITIS: A SYSTEMATIC REVIEW AND META-ANALYSIS	Meeting abstract
196	The safety and efficacy of nasobiliary drainage versus biliary stenting in malignant biliary obstruction A systematic review and meta-analysis	Not a systematic review or meta analysis of randomized controlled trials
197	The treatment of critically ill patients with acute cholecystitis - A systematic review and meta-analysis comparing percutaneous cholecystostomy and cholecystectomy	Topics related to surgical resection or surgical biliary reconstruction
198	The value of preoperative biliary drainage in hilar cholangiocarcinoma: A systematic review and meta analysis of 10 years' literatures	Not a systematic review or meta analysis of randomized controlled trials
199	To drain or not to drain: A meta-analysis on the efficacy of preoperative biliary drainage for tumors causing obstructive jaundice	Meeting abstract
200	TREATMENT OF MALIGNANT HILAR BILIARY OBSTRUCTION IN CHOLANGIOCARCINOMA – A META-ANALYTIC COMPARISON OF ENDOSCOPIC STENT-IN-STENT VERSUS SIDE-BY-SIDE INSERTION OF SELF-EXPANDING METAL STENTS	Meeting abstract
201	T-tube biliary reconstruction in liver transplantation: Useful or harmful? A meta analysis	Topics related to surgical resection or surgical biliary reconstruction

202	T-tube or no T-tube for biliary tract reconstruction in orthotopic liver transplantation: an updated systematic review and meta-analysis	Topics related to surgical resection or surgical biliary reconstruction
203	T-Tube or no T-tube in the reconstruction of the biliary tract during orthotopic liver transplantation: systematic review and meta-analysis	Topics related to surgical resection or surgical biliary reconstruction
204	T-Tube Use After Laparoscopic Common Bile Duct Exploration	Not a systematic review or meta analysis of randomized controlled trials
205	T-tube vs no T-tube for biliary tract reconstruction in adult orthotopic liver transplantation: An updated systematic review and meta-analysis	Topics related to surgical resection or surgical biliary reconstruction
206	UNILATERAL VERSUS BILATERAL ENDOSCOPIC STENTING IN PATIENTS WITH UNRESECTABLE MALIGNANT HILAR OBSTRUCTION: A SYSTEMATIC REVIEW AND META-ANALYSIS	Meeting abstract
207	Use of fully covered self-expanding metal stents for management of choledocholithiasis: A systematic review and meta-analysis	Meeting abstract
208	Value of T-tube in biliary tract reconstruction during orthotopic liver transplantation: a meta-analysis	Topics related to surgical resection or surgical biliary reconstruction
209	WHICH TECHNIQUE IS BETTER: EUS VS ERCP GUIDED BILIARY DRAINAGE FOR DISTAL MALIGNANT BILIARY OBSTRUCTION: A SYSTEMATIC REVIEW AND META-ANALYSIS	Meeting abstract
210	Y-CONFIGURATION SELF EXPANDING METAL STENT WITH LARGE INTERSTICES FOR TREATMENT OF CHOLANGIOCARCINOMA MALIGNANT HILAR BILIARY OBSTRUCTION- A META-ANALYSIS	Meeting abstract
211	Covered Self-Expanding Metal Stents Versus Multiple Plastic Stents for Benign Biliary Strictures: An Updated Meta-Analysis of Randomized Controlled Trials	Subject is not malignant biliary obstruction
212	Effect of covered self-expanding metal stents compared with multiple plastic stents on benign biliary stricture: A meta-analysis	Subject is not malignant biliary obstruction
213	Efficacy of self-expandable metal stents in management of benign biliary strictures and comparison with multiple plastic stents: a meta-analysis	Subject is not malignant biliary obstruction
214	Metal versus plastic stents in the management of benign biliary strictures: Systematic review and meta-analysis of randomized controlled trials	Subject is not malignant biliary obstruction
215	Endoscopic treatment of anastomotic biliary stricture after adult deceased donor liver transplantation with multiple plastic stents versus self-expandable metal stents: a systematic review and meta-analysis	Subject is not malignant biliary obstruction
216	Management of anastomotic biliary stricture after liver transplantation: metal versus plastic stent	Subject is not malignant biliary obstruction
217	Metallic vs plastic stents to treat biliary stricture after liver transplantation: a systematic review and meta-analysis based on randomized trials	Subject is not malignant biliary obstruction
218	Multiple plastic versus fully covered metal stents for managing post-liver transplantation anastomotic biliary strictures: a meta-analysis of randomized controlled trials	Subject is not malignant biliary obstruction
219	Percutaneous cholecystostomy for high - risk surgical patients with acute calculous cholecystitis	Subject is not malignant biliary obstruction
220	Meta-Analysis of Early Endoscopic Retrograde Cholangiopancreatography (ERCP) ± Endoscopic Sphincterotomy (ES) Versus Conservative Management for Gallstone Pancreatitis (GSP)	Subject is not malignant biliary obstruction
221	Primary closure versus T-tube drainage after common bile duct exploration for choledocholithiasis	Subject is not malignant biliary obstruction
222	Is the end of the T-tube drainage era in laparoscopic choledochotomy for common bile duct stones is coming? A systematic review and meta-analysis	Subject is not malignant biliary obstruction
223	T-tube drainage versus primary closure after laparoscopic common bile duct exploration	Subject is not malignant biliary obstruction
224	Primary closure versus T-tube drainage in laparoscopic common bile duct exploration: A meta-analysis of randomized clinical trials	Subject is not malignant biliary obstruction
225	Primary duct closure versus T-tube drainage after laparoscopic common bile duct exploration: a meta-analysis	Subject is not malignant biliary obstruction
226	Should T-Tube Drainage be Performed for Choledocholithiasis after Laparoscopic Common Bile Duct Exploration? A Systematic Review and Meta-Analysis of Randomized Controlled Trials	Subject is not malignant biliary obstruction
227	Can T-tube drainage be replaced by primary suture technique in laparoscopic common bile duct exploration? A meta-analysis of randomized controlled trials	Subject is not malignant biliary obstruction
228	Primary closure versus T-tube drainage after open common bile duct exploration	Subject is not malignant biliary obstruction
229	T-tube drainage versus primary closure after open common bile duct exploration.	Subject is not malignant biliary obstruction
230	Palliative biliary stents for obstructing pancreatic carcinoma	Subject is not malignant biliary obstruction

Table S3: Citation matrices for meta-analyses with overlapping associations

3-1 Malignant biliary obstruction -- radioactive stent implantation

Meta-analysis	Xu, 2018	Xiang, 2021	Sha, 2021	Chen, 2021
Overlapping associations	Malignant biliary obstruction -- radioactive stent implantation			
Individual study				
Hasimu, 2016	√	√	√	√
Zhu, 2012	√	√	√	√
Yao, 2014	√	√	√	×
Liu, 2016	√	√	√	×
Chen, 2012	√	√	√	√
Jiao, 2017	×	√	√	√
Zhu, 2018	×	√	√	√
Chen, 2018	×	√	√	√
Zhou, 2018	×	√	×	×
Zhao, 2015	×	√	×	×
Ren, 2015	×	√	×	×
Total (No of publications per meta-analysis)	5	11	8	6
Grand Total (N)	30			
Rows (r)	11			
Columns (c)	4			
Corrected covered area (CCA)%	57.58%			
Formula of CAA, CCA (%) = N-r / rc-r. [N = number of included publications; r = number of rows (Individual publications); c = number of columns (number of meta-analyses)]				

3-2 Malignant biliary obstruction -- self expanding stent vs. plastic stent

Meta-analysis	Moole, 2017	Yuan, 2017	Hong, 2013	Almadi, 2017	Sawas, 2015	Moss, 2007	Scatimburgo, 2021	Pu, 2015
Overlapping associations	Malignant biliary obstruction -- self expanding stent vs. plastic stent							
Individual study								
Moses, 2013	√	√	×	√	√	√	√	√
Soderlund, 2006	√	√	√	√	√	×	√	√
Kaassis, 2003	√	×	√	√	√	√	√	√
Prat, 1998	√	√	√	√	√	√	√	√
Lammer, 1996	√	√	√	√	√	×	×	×
Knyrim, 1992	×	×	×	√	×	×	×	×
Knyrim, 1993	√	×	√	√	√	√	√	√
Davids, 1992	√	√	√	√	√	√	√	√
Wagner, 1993	×	√	√	√	√	×	×	√
Pinol, 2002	×	√	×	√	√	×	×	×
Sangchan, 2012	×	√	√	√	√	×	×	√
Mukai, 2013	×	√	√	√	√	×	×	√
Schmidt, 2014	×	√	×	√	×	×	√	×
Katsinelos, 2006	×	×	√	√	√	×	√	√
Walter, 2014	×	×	×	×	×	×	×	√
Walter, 2015	×	×	×	√	×	×	√	×
Walter, 2017	×	×	×	×	×	×	√	×
Bernon, 2012	×	×	×	√	×	×	×	√
Isayama, 2011	×	×	×	√	×	×	√	√
Travis, 1997	×	×	×	√	×	×	×	×
Carr-Locke, 1993	×	×	×	√	√	√	×	×
Rösch, 1997	×	×	×	√	×	√	×	×
Bernon, 2018	×	×	×	×	×	×	√	×
Total (No of publications per meta-analysis)	7	10	10	20	13	7	12	13
Grand Total (N)	92							
Rows (r)	23							
Columns (c)	8							
Corrected covered area (CCA)%	42.86%							
Formula of CAA, CCA (%) = N-r / rc-r. [N = number of included publications; r = number of rows (Individual publications); c = number of columns (number of meta-analyses)]								

3-3 Malignant biliary obstruction -- covered stent vs. uncovered stent

Meta-analysis	Chen, 2016	Yamashita, 2022	Moole, 2016	Tringali, 2018	Saleem, 2011
Overlapping associations	Malignant biliary obstruction -- covered stent vs. uncovered stent				
Individual study					
Kullman, 2010	√	√	√	√	√
Krokidis, 2010	√	×	√	√	√
Krokidis, 2011	√	×	√	√	√
Kitano, 2013	√	√	√	√	×
Ung, 2013	√	√	√	√	×
Yang, 2015	√	√	×	√	×
Isayama, 2004	×	√	√	√	√
Telford, 2010	×	√	√	√	√
Conio, 2018	×	√	×	×	×
Seo, 2019	×	√	×	×	×
Sakai, 2021	×	√	×	×	×
Lee, 2014	×	×	√	√	×
Gonzales-Huix 2008	×	×	×	√	×
Smits Me 1995	×	×	×	√	×
Total (No of publications per meta-analysis)	6	9	8	11	5
Grand Total (N)	39				
Rows (r)	14				
Columns (c)	5				
Corrected covered area (CCA)%	44.64%				
Formula of CAA, CCA (%) = N-r / rc-r. [N = number of included publications; r = number of rows (Individual publications); c = number of columns (number of meta-analyses)]					

3-4 Malignant biliary obstruction -- EUS vs. PTCD(PTBD)

Meta-analysis	Duan, 2017	Zhao, 2015	Leng, 2014	Sharaiha, 2017	Miller, 2019
Overlapping associations	Malignant biliary obstruction -- EUS vs. PTCD				
Individual study					
Speer, 1987	√	√	√	×	×
Pinol, 2002	√	√	√	×	×
Saluja, 2008	√	√	√	×	×
Artifon, 2012	×	×	×	√	√
Giovannini, 2015	×	×	×	√	√
Lee, 2016	×	×	×	√	√
Total (No of publications per meta-analysis)	3	3	3	3	3
Grand Total (N)	15				
Rows (r)	6				
Columns (c)	5				
Corrected covered area (CCA)%	37.5%				
Formula of CAA, CCA (%) = N-r / rc-r. [N = number of included publications; r = number of rows (Individual publications); c = number of columns (number of meta-analyses)]					

3-5 Malignant biliary obstruction -- EUS-BD vs.ERCP-BD

Meta-analysis	Logiudice, 2019	Han, 2019	Li, 2019	Miller, 2019
Overlapping associations	Malignant biliary obstruction -- EUS-BD vs.ERCP-BD			
Individual study				
Bang, 2018	√	√	√	√
Paik, 2018	√	√	√	√
Park, 2018	√	√	√	√
Total (No of publications per meta-analysis)	3	3	3	3
Grand Total (N)	12			
Rows (r)	3			
Columns (c)	4			
Corrected covered area (CCA)%	100.00%			
Formula of CAA, CCA (%) = N-r / rc-r. [N = number of included publications; r = number of rows (Individual publications); c = number of columns (number of meta-analyses)]				

3-6 Preoperative biliary drainage of obstructive jaundice

Meta-analysis	Sewnath, 2002	Fang, 2013	Fang, 2013(Cochrane)	Scheufele, 2017	Chen, 2015
Overlapping associations	Preoperative biliary drainage of obstructive jaundice				
Individual study					
Hatfifield, 1982	√	×	×	×	×
McPherson, 1984	√	√	√	×	×
Eshuis, 2010	×	×	×	√	×
Lai, 1994	√	√	√	√	√
van der Gaag, 2010	×	√	√	√	√
Hatfield, 1982	×	√	√	×	√
Lygidakis, 1987	×	×	×	×	√
McPherson, 1984	×	×	×	×	√
Pitt, 1985	√	√	√	×	√
Smith, 1985	√	×	×	×	√
Wig, 1999	×	√	√	×	√
Total (No of publications per meta-analysis)	5	6	6	3	8
Grand Total (N)	28				
Rows (r)	11				
Columns (c)	5				
Corrected covered area (CCA)%	38.64%				
Formula of CAA, CCA (%) = N-r / rc-r. [N = number of included publications; r = number of rows (Individual publications); c = number of columns (number of meta-analyses)]					

3-7 Preoperative periampullary carcinoma -- metal stent vs. plastic stent

Meta-analysis	Zhang, 2021	Watanabe, 2022
Overlapping associations	Preoperative periampullary carcinoma -- metal stent vs. plastic stent	
Individual study		
Cho, 2020	√	√
Gonz, 2014	√	√
Olsson, 2017	√	√
Song, 2016	√	√
Tol, 2015	√	×
Gardner, 2016	×	√
Mandai, 2021	×	√
Tamura, 2021	×	√
Total (No of publications per meta-analysis)	5	7
Grand Total (N)	12	
Rows (r)	8	
Columns (c)	2	
Corrected covered area (CCA)%	50.00%	
Formula of CAA, CCA (%) = N-r / rc-r. [N = number of included publications; r = number of rows (Individual publications); c = number of columns (number of meta-analyses)]		

TableS4. Data extraction form for included systematic reviews or meta analyses and the corresponding individual studies.

S4-1: Data extraction form for included systematic reviews or meta analyses

Systematic reviews or meta analyses	
1	First author
2	Country
3	Publication year
4	Journal name
5	Original article retrieval time
6	Total No. of included studies
7	Individual studies design
8	Diseases type
9	Total No. of patients
10	Intervention (No.of cases)
11	Control (No.of cases)
12	Quality appraisal tool
13	Information of funding
14	Conflict of interests
15	Clinical Outcome
16	Effects model
17	MA metric
18	Estimates
19	95%CI
20	Publication bias
21	Heterogeneity

S4-2: Data extraction form for the corresponding individual studies

The corresponding individual studies	
1	First author
2	Publication year
3	Intervention (Event/Total)
4	Control (Event/Total)
5	HR/RR/OR value
6	95%CI

TableS5. AMSTAR2 was used to evaluate the methodological quality of the included literature

Study	Q 1	Q 2*	Q 3	Q 4*	Q 5	Q 6	Q 7*	Q 8	Q 9*	Q 10	Q 11*	Q 12	Q 13*	Q 14	Q 15*	Q 16	AMSTAR-2 overall quality
Xu,2018	Y	N	N	PY	N	N	N	Y	Y	N	Y	Y	Y	Y	Y	Y	Critically Low
Xiang,2021	Y	N	N	PY	Y	Y	N	PY	Y	N	Y	N	N	Y	Y	Y	Critically Low
Sha,2021	Y	N	N	PY	N	Y	N	Y	Y	N	Y	Y	Y	Y	Y	Y	Critically Low
Chen,2021	Y	N	N	Y	Y	Y	N	PY	Y	N	Y	Y	Y	Y	Y	Y	Critically Low
Moole,2017	Y	N	N	Y	N	Y	N	N	N	N	N	N	N	N	Y	Y	Critically Low
Yuan,2017	Y	N	Y	PY	N	N	N	PY	N	N	N	N	N	N	Y	Y	Critically Low
Hong,2013	Y	N	Y	PY	N	N	N	N	PY	N	N	N	N	N	Y	Y	Critically Low
Almadi,2017	Y	N	Y	Y	N	Y	N	Y	Y	N	Y	Y	Y	Y	Y	Y	Critically Low
Sawas,2015	Y	N	N	PY	N	Y	N	PY	PY	N	Y	N	N	Y	Y	Y	Critically Low
Moss, 2007	Y	N	Y	Y	Y	N	N	PY	N	N	N	N	N	N	N	Y	Critically Low
Scatimburgo,2021	Y	Y	Y	Y	Y	Y	N	Y	Y	N	Y	Y	Y	Y	Y	Y	Low
Pu,2015	Y	Y	N	Y	Y	Y	Y	PY	PY	N	N	Y	Y	N	Y	Y	Low
Duan,2017	Y	N	N	Y	Y	Y	N	PY	PY	N	N	N	N	Y	Y	Y	Critically Low
Zhao,2015	Y	N	N	PY	N	Y	N	Y	PY	N	Y	N	N	Y	N	Y	Critically Low
Leng,2014	Y	N	N	Y	Y	Y	N	Y	PY	N	Y	N	N	Y	Y	Y	Critically Low
Sharaiha,2017	Y	N	N	Y	Y	Y	N	PY	PY	N	Y	Y	Y	Y	Y	Y	Critically Low
Miller,2019*	Y	N	Y	PY	Y	Y	N	PY	PY	N	Y	N	N	Y	Y	Y	Critically Low
Yuan,2019	Y	N	N	PY	N	Y	N	Y	N	N	N	N	N	N	Y	Y	Critically Low
Song,2022	Y	Y	N	Y	N	Y	N	Y	Y	N	Y	Y	Y	Y	Y	Y	Low
Renno,2019	Y	N	N	PY	Y	N	N	PY	PY	N	Y	N	N	Y	Y	Y	Critically Low
Chen,2016	Y	N	N	PY	Y	Y	N	PY	N	N	N	N	N	N	Y	Y	Critically Low
Yamashita,2022	Y	N	N	Y	Y	N	N	PY	PY	N	Y	N	N	Y	Y	Y	Critically Low
Moole,2016	Y	N	Y	Y	Y	Y	N	PY	PY	N	Y	N	Y	Y	Y	Y	Critically Low
Tringali,2018	Y	Y	N	PY	Y	Y	N	Y	Y	N	N	N	N	N	Y	Y	Critically Low
Saleem,2011	Y	Y	Y	Y	Y	Y	N	Y	PY	N	N	N	N	N	Y	Y	Critically Low
Logiudice,2019	Y	Y	Y	Y	Y	N	N	PY	Y	N	Y	Y	Y	Y	Y	Y	Low
Han,2019	Y	N	N	PY	Y	Y	N	PY	PY	N	N	N	N	N	Y	Y	Critically Low
Li,2019	Y	Y	N	Y	Y	Y	N	Y	PY	N	N	N	N	N	Y	Y	Critically Low
Cui,2014	Y	N	N	PY	N	Y	N	PY	PY	N	N	N	N	N	N	Y	Critically Low
Zhang,2021	Y	N	Y	PY	N	Y	N	Y	PY	N	Y	N	N	Y	Y	Y	Critically Low
Watanabe,2022	Y	Y	Y	Y	N	Y	N	PY	Y	N	N	Y	Y	N	Y	Y	Critically Low
Sewnath,2002	Y	N	N	Y	Y	Y	N	PY	PY	N	N	Y	Y	N	N	Y	Critically Low
Fang,2013	Y	N	N	Y	Y	Y	N	PY	Y	N	Y	Y	Y	N	Y	Y	Critically Low
Fang,2013 (Cochrane)	Y	Y	N	Y	Y	Y	Y	Y	Y	Y	Y	Y	Y	Y	Y	Y	High
Scheufele,2017	Y	N	N	PY	Y	Y	N	PY	PY	N	N	Y	Y	N	Y	Y	Critically Low
Chen,2015	Y	N	Y	PY	N	Y	N	PY	N	N	N	N	N	N	Y	Y	Critically Low

AMSTAR-2 items: Q1: Did the research questions and inclusion criteria for the review include the components of PICO? Q2: Did the report of the review contain an explicit statement that the review methods were established prior to the conduct of the review, and did the report justify any signifificant deviations from the protocol? Q3: Did the review authors explain their selection of the study designs for inclusion in the review? Q4: Did the review authors use a comprehensive Literature search strategy? Q5: Did the review authors perform study selection in duplicate? Q6: Did the review authors perform data extraction in duplicate? Q7: Did the review authors provide a list of excluded studies and justify the exclusions? Q8: Did the review authors describe the included studies in adequate detail? Q9: Did the review authors use a satisfactory technique for assessing the risk of bias (RoB) in individual studies that were included in the review? Q10: Did

the review authors report on the sources of funding for the studies included in the review? Q11: If meta-analysis was performed, did the review authors use appropriate methods for statistical combination of results? Q12: If meta-analysis was performed, did the review authors assess the potential impact of RoB in individual studies on the results of the meta-analysis or other evidence synthesis? Q13: Did the review authors account for RoB in primary studies when interpreting/discussing the results of the review? Q14: Did the review authors provide a satisfactory explanation for, and discussion of, any heterogeneity observed in the results of the review? Q15: If they performed quantitative synthesis, did the review authors carry out an adequate investigation of publication bias (small study bias) and discuss its likely impact on the results of the review? Q16: Did the review authors report any potential sources of conflict of interest, including any funding they received for conducting the review?

Table S6: General characteristics of meta-analyses with overlapping associations

First author, Year	Overlapping associations	No of publications per meta-analysis	AMSTAR-2 overall quality	Corrected covered area (CCA)	Decision to retain(Y or N)
Xu,2018	MBO -- radioactive stent implantation	5	Critically Low	57.58%	N
Xiang,2021		11	Critically Low		Y
Sha,2021		8	Critically Low		N
Chen,2021		6	Critically Low		N
Moole,2017	MBO -- self expanding stent vs. plastic stent	7	Critically Low	42.86%	N
Yuan,2017		10	Critically Low		N
Hong,2013		10	Critically Low		N
Almadi,2017		20	Critically Low		Y
Sawas,2015		13	Critically Low		N
Moss,2007		7	Critically Low		N
Scatimburgo,2021		12	Low		N
Pu,2015		13	Low		N
Chen,2016	MBO -- covered stent vs. uncovered stent	6	Critically Low	44.64%	N
Yamashita,2022		9	Critically Low		N
Moole,2016		8	Critically Low		N
Tringali,2018		11	Critically Low		Y
Saleem,2011		5	Critically Low		N
Duan,2017	MBO -- EUS vs. PTCD	3	Critically Low	37.50%	Y
Zhao,2015		3	Critically Low		N
Leng,2014		3	Critically Low		N
Sharaiha,2017		3	Critically Low		Y
Miller,2019*		3	Critically Low		N
Logiudice,2019	MBO -- EUS-BD vs.ERCP-BD	3	Low	100.00%	Y
Han,2019		3	Critically Low		N
Li,2019		3	Critically Low		N
Miller,2019*		3	Critically Low		N
Sewnath,2002	Preoperative biliary drainage of obstructive jaundice	5	Critically Low	38.64%	N
Fang,2013		6	Critically Low		N
Fang,2013 (Cochrane)		6	High		Y
Scheufele,2017		3	Critically Low		N
Chen,2015		8	Critically Low		N
Zhang,2021	Preoperative periampullary carcinoma -- metal stent vs. plastic stent	5	Critically Low	50.00%	N
Watanabe,2022		7	Critically Low		Y

TableS7. List of studies included in analysis and meta-analyses with overlapping associations excluded from analysis.

S7-1 List of studies included in analysis

1	Xiang,2021	Iodine-125 seeds combined with biliary stent placement versus stent placement alone for unresectable malignant biliary obstruction: A meta-analysis of randomized controlled trials
2	Yuan,2019	Efficacy and safety evaluation of paclitaxel-loaded metal stents in patients with malignant biliary obstructions
3	Song,2022	Local palliative therapies for unresectable malignant biliary obstruction: radiofrequency ablation combined with stent or biliary stent alone? An updated meta-analysis of nineteen trials
4	Almadi,2017	Plastic vs. Self-Expandable Metal Stents for Palliation in Malignant Biliary Obstruction: A Series of Meta-Analyses
5	Renno,2019	Antireflux valve metal stent versus conventional self-expandable metal stent in distal malignant biliary obstruction: a systematic review and meta-analysis
6	Tringali,2018	Covered vs. uncovered self-expandable metal stents for malignant distal biliary strictures: a systematic review and meta-analysis
7	Duan,2017	Comparison of efficacy and complications of endoscopic and percutaneous biliary drainage in malignant obstructive jaundice: A systematic review and meta-analysis
8	Sharaiha,2017	Efficacy and safety of EUS-guided biliary drainage in comparison with percutaneous biliary drainage when ERCP fails: a systematic review and meta-analysis
9	Logiudice,2019	Endoscopic ultrasound-guided vs endoscopic retrograde cholangiopancreatography biliary drainage for obstructed distal malignant biliary strictures: A systematic review and meta-analysis
10	Cui,2014	Biliary stenting with or without sphincterotomy for malignant biliary obstruction: a meta-analysis
11	Fang,2013	Pre-operative biliary drainage for obstructive jaundice
12	Watanabe,2022	Metal vs plastic stents for preoperative biliary drainage in patients with periampullary cancer: An updated systematic review and meta-analysis
13	Minaga,2019	Comparison of the efficacy and safety of endoscopic ultrasound-guided choledochoduodenostomy and hepaticogastrostomy for malignant distal biliary obstruction: Multicenter, randomized, clinical trial
14	Ryunosuke,2021	Unilateral versus Bilateral Endoscopic Nasobiliary Drainage and Subsequent Metal Stent Placement for Unresectable Malignant Hilar Obstruction: A Multicenter Randomized Controlled Trial
15	Choi,2022	Acetylsalicylic acid for metal stent in malignant distal common bile duct obstruction: A randomized controlled trial
16	Fu,2019	Percutaneous stenting for malignant hilar biliary obstruction: a randomized controlled trial of unilateral versus bilateral stenting

S7-2 List of meta-analyses with overlapping associations excluded from analysis

1	Xu,2018	A Systematic Review and Meta-analysis of Intraluminal Brachytherapy Versus Stent Alone in the Treatment of Malignant Obstructive Jaundice
2	Sha,2021	Irradiation stent insertion for inoperable malignant biliary obstruction: a meta-analysis of randomized controlled trials
3	Chen,2021	Percutaneous biliary stent combined with brachytherapy using I-125 seeds for treatment of unresectable malignant obstructive jaundice: A meta-analysis
4	Moole,2017	Are self-expandable metal stents superior to plastic stents in palliating malignant distal biliary strictures? A meta-analysis and systematic review
5	Yuan,2017	Comparison of plastic stents with self-expandable metal stents in palliative treatment of malignant biliary obstruction: a meta-analysis
6	Hong,2013	Metal versus plastic stents for malignant biliary obstruction: an update meta-analysis
7	Sawas,2015	Self-expandable metal stents versus plastic stents for malignant biliary obstruction: a meta-analysis
8	Moss, 2007	Do the benefits of metal stents justify the costs? A systematic review and meta-analysis of trials comparing endoscopic stents for malignant biliary obstruction
9	Scatimburgo,2021	Biliary drainage in inoperable malignant biliary distal obstruction: A systematic review and meta-analysis
10	Pu,2015	Endoscopic stenting for inoperable malignant biliary obstruction: A systematic review and meta-analysis
11	Zhao,2015	Comparison of percutaneous transhepatic biliary drainage and endoscopic biliary drainage in the management of malignant biliary tract obstruction: a meta-analysis
12	Leng,2014	Percutaneous transhepatic and endoscopic biliary drainage for malignant biliary tract obstruction: a meta-analysis
13	Miller,2019	Endoscopic ultrasound-guided biliary drainage for distal malignant obstruction: a systematic review and meta-analysis of randomized trials
14	Chen,2016	Covered Stents versus Uncovered Stents for Unresectable Malignant Biliary Strictures: A Meta-Analysis
15	Yamashita,2022	Covered versus uncovered metal stent for endoscopic drainage of a malignant distal biliary obstruction: Meta-analysis
16	Moole,2016	Covered versus uncovered self-expandable metal stents for malignant biliary strictures: A meta-analysis and systematic review
17	Saleem,2011	Meta-analysis of randomized trials comparing the patency of covered and uncovered self-expandable metal stents for palliation of distal malignant bile duct obstruction
18	Han,2019	EUS-guided biliary drainage versus ERCP for first-line palliation of malignant distal biliary obstruction: A systematic review and meta-analysis
19	Li,2019	Is ERCP-BD or EUS-BD the preferred decompression modality for malignant distal biliary obstruction? A meta-analysis of randomized controlled trials
20	Zhang,2021	Comparison of metal stents versus plastic stents for preoperative biliary drainage: a meta-analysis of five randomized controlled trials
21	Sewnath,2002	A meta-analysis on the efficacy of preoperative biliary drainage for tumors causing obstructive jaundice
22	Fang,2013	Meta-analysis of randomized clinical trials on safety and efficacy of biliary drainage before surgery for obstructive jaundice
23	Scheufele,2017	Preoperative biliary stenting versus operation first in jaundiced patients due to malignant lesions in the pancreatic head: A meta-analysis of current literature
24	Chen,2015	Effect of Preoperative Biliary Drainage on Complications Following Pancreatoduodenectomy: A Meta-Analysis

TableS8. Characteristics and quality assessment of evidence.

Diseases type& Treatment<br>Author, year	Evidence	No. of Studies	No. of patients	Random-efects model<br>Relative risk and 95% CIs	P-value	Heterogeneity<br>I^2	 P-value	Small-study effects
**MBO--I[125]seeds+stent vs. stent**								
Xiang,2021	3-month stent occlusion	7	544	OR, 0.15 [0.05; 0.48]	0.0013	61.80%	0.0153	0.0450
Xiang,2021	6-month stent occlusion	7	544	OR, 0.18 [0.08; 0.44]	0.0001	64.10%	0.0103	0.0275
Xiang,2021	9-month stent occlusion	5	214	OR, 0.10 [0.05; 0.21]	<0.0001	0.00%	0.8818	0.4707
Xiang,2021	1-year stent occlusion	10	720	OR, 0.15 [0.08; 0.31]	<0.0001	53.80%	0.0214	0.0060
Xiang,2021	Survival	9	681	SMD, 0.98 [0.66; 1.31]	<0.0001	74.80%	0.0001	0.0050
**MBO--PECMS/MSCPM vs. CMS**								
Yuan,2019	Stent patency duration	3	221	HR, 1.30 [0.93; 1.83]	0.1288	24.10%	0.2680	NA
Yuan,2019	Survival time	3	221	HR, 0.85 [0.59; 1.21]	0.3817	31.90%	0.2300	NA
Yuan,2019	Stent malfunction	3	221	RR, 1.09 [0.72; 1.66]	0.6913	1.60%	0.3618	0.7814
Yuan,2019	Stent occlusion caused by tumor in growth	3	221	RR, 1.09 [0.59; 2.03]	0.7744	0.00%	0.9294	0.8675
Yuan,2019	Stent occlusion caused by distal stent	3	221	RR, 0.92 [0.38; 2.20]	0.8495	24.30%	0.2666	0.2480
Yuan,2019	All complications	3	221	RR, 1.17 [0.75; 1.83]	0.4996	0.00%	0.4811	0.3143
Yuan,2019	Pancreatitis	3	221	RR, 0.58 [0.19; 1.79]	0.3406	0.00%	0.7403	0.3911
Yuan,2019	Cholangitis-like symptoms	3	221	RR, 1.80 [0.66; 4.94]	0.2517	6.10%	0.3448	0.0080
**MBO--RFA+Stent vs. Stent alone**								
Song,2022	Overall survival	3	287	HR, 0.41 [0.21; 0.78]	0.0072	65.80%	0.0539	0.6539
Song,2022	Mean survival time	3	287	SMD, 5.03 [0.94, 9.12]	0.0200	99.00%	NA	NA
Song,2022	Mean stent patency time	3	287	SMD, 0.63 [-1.77; 3.04]	0.6061	98.50%	<0.0001	0.1131
Song,2022	Stent patency rate at 3 months	2	222	OR, 0.90 [0.36; 2.25]	0.8297	52.10%	0.1485	–
Song,2022	Stent patency rate at 6 months	2	222	OR, 1.00 [0.44; 2.25]	0.9922	29.50%	0.2338	–
Song,2022	Alleviation of total bilirubin	1	65	SMD, 0.50 [0.01; 1.00]	0.0500	–	–	–
Song,2022	Alleviation of direct bilirubin	1	65	SMD, 0.16 [-0.33; 0.64]	0.5300	–	–	–
Song,2022	Abdominal pain	1	174	OR, 2.07 [0.50; 8.57]	0.3095	–	–	–
Song,2022	Mild bleeding	2	239	OR, 0.33 [0.05; 2.12]	0.2418	0.00%	0.9907	–
Song,2022	Cholangitis	3	287	OR, 0.82 [0.40; 1.66]	0.5756	0.00%	0.4824	0.0271
Song,2022	Pancreatitis	3	287	OR, 0.54 [0.17; 1.71]	0.2973	0.00%	0.5247	0.2947
**MBO--Self-expandable metal stents vs. Plastic stents**								
Almadi,2017	Duration of stent patency	4	503	SMD, 1.79 [-0.84; 4.42]	0.1813	97.30%	<0.0001	0.1051
Almadi,2017	Duration of patient survival	5	400	SMD, 0.07 [-0.39; 0.54]	0.7597	80.50%	0.0004	0.6717
Almadi,2017	30day mortality	8	712	OR, 0.78 [0.49; 1.22]	0.2762	7.10%	0.3754	0.2621
Almadi,2017	Successful stent insertion*(Fixed)	12	892	OR, 1.23 [0.66; 2.28]	0.5233	0.00%	0.63	0.6500
Almadi,2017	Successful biliary drainage	8	558	OR, 1.79 [0.64; 5.00]	0.2702	51.00%	0.0800	0.2500
Almadi,2017	Early complications	14	1052	OR, 0.65 [0.29; 1.42]	0.2916	67.00%	<0.0100	0.1700
Almadi,2017	Late complications	11	888	OR, 0.43 [0.26; 0.71]	0.0011	58.60%	0.0072	0.8582
Almadi,2017	Total complication	17	1377	OR, 0.40 [0.22; 0.73]	0.0031	72.50%	<0.0001	0.4643
Almadi,2017	Pancreatitis*(Fixed)	11	1025	OR, 0.93 [0.48; 1.80]	0.9656	0.00%	0.8000	0.8300
Almadi,2017	Bleeding*(Fixed)	10	762	OR, 0.90 [0.39; 2.08]	0.8169	0.00%	0.9900	0.8500
Almadi,2017	Sepsis or cholangitis	14	1003	OR, 0.53 [0.31; 0.90]	0.0194	28.80%	0.1478	0.7141

Almadi,2017	Stent migration*(Fixed)	8	792	OR, 0.65 [0.31; 1.39]	0.2635	24.00%	0.2400	0.5600
Almadi,2017	Blockage from sludge*(Fixed)	8	896	OR, 0.11 [0.07; 0.17]	<0.0001	0.00%	0.6100	0.5900
Almadi,2017	Tumor ingrowth*(Fixed)	7	757	OR, 11.66 [3.75; 36.26]	<0.0001	0.00%	0.9700	0.1100
Almadi,2017	Tumor overgrowth*(Fixed)	7	821	OR, 1.90 [0.79; 4.58]	0.1527	19.00%	0.2800	0.2600
Almadi,2017	Hospital re-admission*(Fixed)	2	91	OR, 1.10 [0.43; 2.85]	0.8540	55.00%	0.14	−
Almadi,2017	Rate of re-interventions	8	689	OR, 0.37 [0.16; 0.81]	0.0129	74.70%	0.0003	0.9767
Almadi,2017	Mean number of re-interventions	5	342	WMD,−0.83[−1.64;−0.02]	NA	95.00%	<0.0100	0.2500
Almadi,2017	1-month symptom free	4	334	OR, 0.29 [0.02; 5.42]	0.3933	92.00%	<0.0100	0.3100
Almadi,2017	3-month symptom free	3	236	OR, 2.16 [0.67; 6.98]	0.1958	67.00%	0.0481	0.1442
Almadi,2017	6-month symptom free	4	296	OR, 5.99 [1.67; 21.51]	0.0060	77.00%	0.0049	0.1936
Almadi,2017	12-month symptom free	3	320	OR, 2.90 [0.22; 37.65]	0.4148	83.30%	0.0025	0.0234
**MBO--ARVMS vs. SEMS**								
Renno,2019	Technical success	3	293	OR, 0.13 [0.01; 1.06]	0.0571	−	−	−
Renno,2019	Clinical success	2	189	OR, 1.30 [0.48; 3.51]	0.6098	0.00%	0.8621	−
Renno,2019	All adverse events	3	285	OR, 0.62 [0.33; 1.17]	0.1396	15.70%	0.3054	0.5720
Renno,2019	Early adverse events	2	189	OR, 0.70 [0.26; 1.85]	0.4719	0.00%	0.4170	−
Renno,2019	Late adverse events	2	184	OR, 0.45 [0.20; 1.03]	0.0581	5.50%	0.3037	−
Renno,2019	Overall stent dysfunction	3	280	OR, 0.77 [0.31; 1.93]	0.5787	71.90%	0.0283	0.9819
Renno,2019	Stent migration	3	280	OR, 2.69 [1.17; 6.15]	0.0193	3.80%	0.3537	0.9266
Renno,2019	Stent occlusion	3	280	OR, 0.45 [0.25; 0.79]	0.0060	2.90%	0.3572	0.0693
**MBO--Covered-SEMS vs. Uncovered-SEMS**								
Tringali,2018	Stent failure	8	1044	HR, 0.68 [0.40; 1.17]	0.1596	74.00%	<0.01	Detected
Tringali,2018	Patient mortality	8	1044	HR, 0.89 [0.76; 1.05]	0.1581	28.00%	0.2000	Detected
Tringali,2018	Stent migration--C-SEMS/PC-SEMS vs.U-SEMS	10	1204	OR, 7.70 [2.46; 24.12]	0.0005	0.03%	0.9825	< 0.0001
Tringali,2018	Stent migration--C-SEMS vs.U-SEMS	6	814	OR, 10.60 [1.93; 58.30]	0.0066	0.00%	0.8854	0.0699
Tringali,2018	Stent migration--PC-SEMS vs.U-SEMS	4	390	OR, 5.92 [1.27; 27.62]	0.0235	0.00%	0.8993	0.0168
Tringali,2018	Tumor ingrowth--C-SEMS/PC-SEMSvs.U-SEMS	9	1090	OR, 0.18 [0.07; 0.43]	0.0001	37.80%	0.1170	0.0058
Tringali,2018	Tumor ingrowth--C-SEMS vs.U-SEMS	5	700	OR, 0.11 [0.03; 0.45]	0.0020	45.50%	0.1191	0.0330
Tringali,2018	Tumor ingrowth--PC-SEMS vs.U-SEMS	4	390	OR, 0.26 [0.08; 0.84]	0.0249	42.20%	0.1583	0.2033
Tringali,2018	Tumor overgrowth--C-SEMS/PC-SEMS vs.U-SEMS	9	1090	OR, 1.96 [1.13; 3.39]	0.0161	0.00%	0.9504	0.4472
Tringali,2018	Tumor overgrowth--C-SEMS vs.U-SEMS	5	700	OR, 1.75 [0.93; 3.28]	0.0825	0.00%	0.7524	0.9716
Tringali,2018	Tumor overgrowth--PC-SEMSvs.U-SEMS	4	390	OR, 2.78 [0.92; 8.40]	0.0701	0.00%	0.9587	0.3832
Tringali,2018	Sludge formation--C-SEMS/PC-SEMSvs.U-SEMS	8	1044	OR, 2.46 [1.37; 4.43]	0.0027	0.00%	0.9815	0.3994
Tringali,2018	Sludge formation--C-SEMS vs.U-SEMS	5	700	OR, 2.55 [1.29; 5.03]	0.0071	0.00%	0.9579	0.7702
Tringali,2018	Sludge formation--PC-SEMS vs.U-SEMS	3	344	OR, 2.21 [0.69; 7.16]	0.1842	0.00%	0.6585	0.2154
Tringali,2018	Cholecystitis--C-SEMS/PC-SEMS vs.U-SEMS	11	1212	OR, 1.50 [0.72; 3.14]	0.2841	0.00%	NA	NA
Tringali,2018	Cholecystitis--C-SEMSvs.U-SEMS	8	932	OR, 1.32 [0.47; 3.76]	0.6131	0.00%	NA	NA
Tringali,2018	Cholecystitis--PC-SEMSvs.U-SEMS	3	280	OR, 1.71 [0.60; 4.84]	0.3184	0.00%	NA	NA
Tringali,2018	Cholangitis--C-SEMS/PC-SEMS vs. U-SEMS	4	729	OR, 0.95 [0.51; 1.79]	0.8821	16.00%	NA	NA
Tringali,2018	Cholangitis--C-SEMS vs.U-SEMS	2	514	OR, 0.93 [0.45; 1.93]	0.8556	25.00%	NA	−
Tringali,2018	Cholangitis--PC-SEMS vs.U-SEMS	2	215	OR, 1.30 [0.14; 12.03]	0.8288	0.00%	NA	−

Tringali,2018	Pancreatitis--C-SEMS/PC-SEMS vs.U-SEMS	10	1226	OR, 1.32 [0.59; 2.93]	0.4998	0.00%	0.6211	0.9131
Tringali,2018	Pancreatitis--C-SEMS vs.U-SEMS	7	882	OR, 0.93 [0.30; 2.84]	0.8986	0.00%	0.6748	0.3319
Tringali,2018	Pancreatitis--PC-SEMS vs.U-SEMS	3	344	OR, 1.90 [0.60; 5.99]	0.2722	5.40%	0.3476	0.7129
Tringali,2018	Perforation--C-SEMS/PC-SEMS vs.U-SEMS	4	746	OR, 1.76 [0.40; 7.68]	0.4622	0.00%	NA	NA
Tringali,2018	Perforation--C-SEMSvs.U-SEMS	2	514	OR, 1.52 [0.18; 12.43]	0.7115	0.00%	NA	–
Tringali,2018	Perforation--PC-SEMSvs.U-SEMS	2	232	OR, 2.03 [0.26; 16.01]	0.5108	0.00%	NA	–
Tringali,2018	Bleeding--C-SEMS/PC-SEMS vs.U-SEMS	6	884	OR, 0.78 [0.24; 2.48]	0.6898	0.00%	NA	NA
Tringali,2018	Bleeding--C-SEMSvs.U-SEMS	3	540	OR, 0.61 [0.14; 2.77]	0.5269	0.00%	NA	NA
Tringali,2018	Bleeding--PC-SEMS vs.U-SEMS	3	344	OR, 1.09 [0.18; 6.81]	0.9321	0.00%	NA	NA
**MBO-- EUS-BD vs. -PTBD**								
Duan,2017	Therapeutic success rate	3	178	OR, 2.33 [0.32; 17.12]	0.4050	87.80%	0.0003	0.3867
Duan,2017	Overall complication	3	178	OR, 1.56 [0.22; 11.12]	0.6575	88.20%	0.0002	0.0080
Duan,2017	30-day mortality rate	2	129	OR, 1.75 [0.28; 10.75]	0.5467	49.50%	0.1594	–
Duan,2017	Cholangitis	2	124	OR, 0.33 [0.06; 1.82]	0.2034	69.00%	0.0723	–
Sharaiha,2017	Technical success	3	132	OR, 0.68 [0.14; 3.42]	0.6419	0.00%	0.4524	0.8972
Sharaiha,2017	Clinical success	2	91	OR, 0.96 [0.24; 3.81]	0.9489	0.00%	0.9867	–
Sharaiha,2017	Postprocedure adverse events	3	132	OR, 0.25 [0.10; 0.61]	0.0023	0.00%	0.6986	0.0185
Sharaiha,2017	Rate of re-intervention	1	66	OR, 0.05[0.01; 0.25]	0.0003	–	–	–
Sharaiha,2017	Length of stay in hospital	2	107	SMD, -1.04 [-1.50; -0.57]	<0.0001	21.60%	0.2586	–
**MBO--EUS-BD vs. ERCP-BD**								
Logiudice,2019	Technical success	3	222	RR, 1.00 [0.93; 1.08]	0.9451	0.00%	0.5640	0.3391
Logiudice,2019	Clinical success	2	155	RR, 0.99 [0.87; 1.13]	0.9045	0.00%	0.9495	–
Logiudice,2019	Duration of the procedure	3	222	SMD, -0.58 [-2.37; 1.21]	0.5247	0.972	<0.0001	0.6248
Logiudice,2019	Adverse events	3	222	RR, 0.67 [0.16; 2.79]	0.5771	79.30%	0.0280	–
Logiudice,2019	Stent patency	2	97	SMD, 0.08 [-0.62; 0.78]	0.8158	62.20%	0.1039	–
Logiudice,2019	Stent dysfunction	3	155	RR, 0.43 [0.24; 0.77]	0.0045	0.00%	0.7835	0.1629
**MBO--EST vs. no-EST**								
Cui,2014	Successful stent insertion	3	338	OR, 1.53 [0.42; 5.62]	0.5187	–	–	–
Cui,2014	PEP	3	348	OR, 0.38 [0.10; 1.46]	0.1595	31.40%	0.2272	–
Cui,2014	Post-ERCP bleeding	2	256	OR, 9.52 [1.18; 76.93]	0.0345	0.00%	0.7842	–
Cui,2014	Stent migration	2	256	OR, 2.28 [0.34; 15.31]	0.3977	50.30%	0.1559	–
Cui,2014	Stent occlusion	3	338	OR, 1.00 [0.38; 2.67]	0.9924	0.00%	0.7901	0.0117
**Obstructive Jaundice-PBD PBD vs. non-PBD**								
Fang,2013	Mortality	6	520	RR, 1.16 [0.75; 1.78]	0.5019	0.00%	0.7052	0.2491
Fang,2013	Mortality--PTBD	4	237	RR, 1.22 [0.65; 2.29]	0.5416	0.00%	0.4092	0.2372
Fang,2013	Mortality--ERCP	2	283	RR, 1.11 [0.62; 1.99]	0.7262	0.00%	0.8544	–
Fang,2013	Long-term mortality	1	202	HR, 0.90 [0.64; 1.24]	0.5293	–	–	–
Fang,2013	Serious morbidity	6	520	RR, 1.65 [1.21; 2.25]	0.0014	44.20%	0.1104	0.6736
Fang,2013	Serious morbidity--PTBD	4	237	RR, 1.79 [0.83; 3.87]	0.1367	64.20%	0.0388	0.0139
Fang,2013	Serious morbidity--ERCP	2	283	RR, 1.75 [1.25; 2.44]	0.0011	0.00%	0.5511	–

Fang,2013	Hospital stay	2	271	SMD, 0.37 [-0.15; 0.89]	0.1660	73.10%	0.0539	–
**Periampullary Cancer-PBD Metal stent vs. Plastic stent**								
Watanabe,2022	Re-intervention	6	383	RR, 0.42 [0.25; 0.72]	0.0015	0.00%	0.5993	0.2345
Watanabe,2022	PBD-related complications	5	292	RR, 0.74 [0.32; 1.74]	0.4951	63.00%	0.0287	0.6009
Watanabe,2022	Postoperative complications	6	313	RR, 0.73 [0.45; 1.17]	0.1981	57.00%	0.0400	NA
Watanabe,2022	Direct costs	2	79	SMD, -0.64 [-2.27; 1.00]	0.4463	88.00%	0.0040	–
Watanabe,2022	Stent occlusion	6	375	OR, 0.29 [0.15; 0.57]	0.0004	0.00%	0.5662	0.5110
Watanabe,2022	Preoperative cholangitis	5	320	OR, 0.38 [0.08; 1.71]	0.2060	35.30%	0.1860	0.6621
Watanabe,2022	Preoperative pancreatitis	4	298	OR, 2.40 [0.93; 6.20]	0.0714	10.90%	0.3385	0.1896
Watanabe,2022	Operative times	3	123	SMD, -0.33 [-0.81; 0.15]	0.1799	29.50%	0.2423	0.5892
Watanabe,2022	Blood loss volumes	3	123	SMD, 0.62 [-1.52; 2.76]	0.5698	92.50%	<0.0001	0.6205
Watanabe,2022	Postoperative pancreatic fistulas	4	182	OR, 0.78 [0.28; 2.18]	0.6289	0.00%	0.5169	0.8911
Watanabe,2022	Delayed gastric emptying	5	250	OR, 1.30 [0.56; 3.00]	0.5368	0.00%	0.4262	0.0616
Watanabe,2022	Wound infection	3	166	OR, 3.00 [0.82; 10.96]	0.0971	0.00%	0.6510	0.4884
Watanabe,2022	Postoperative bleeding	5	250	OR, 0.64 [0.17; 2.33]	0.4938	0.00%	0.7271	0.5309

TableS9.GRADE and AMSTAR2 result of evidence.

Summary of findings					Certainty assessment(Degradation factor)					Certainty assessment(Escalation factors)			Importance	GRADE	AMSTAR2
First author, Year	Diseases and treatment	Study design (Number)	Outcome	Relative Effect (95% CI)	Risk of bias	Inconsistency	Indirectness	Imprecision	Publication bias	Large effect	Plausible confounding	Dose response gradient			
Xiang,2021	MBO--I^{125}seeds+stent vs. stent	RCT(7)	3-month stent occlusion	OR, 0.15 [0.05; 0.48]	serious[a]	serious[b]	not serious	not serious	strongly suspected	yes	no	no	8-Critical	⊕⊕⊕◯ Moderate	Critically low
Xiang,2021	MBO--I^{125}seeds+stent vs. stent	RCT(7)	6-month stent occlusion	OR, 0.18 [0.08; 0.44]	serious[a]	serious[b]	not serious	not serious	strongly suspected	yes	no	no	8-Critical	⊕⊕⊕◯ Moderate	Critically low
Xiang,2021	MBO--I^{125}seeds+stent vs. stent	RCT(5)	9-month stent occlusion	OR, 0.10 [0.05; 0.21]	serious[a]	not serious	not serious	not serious	undetected	yes	no	no	8-Critical	⊕⊕⊕⊕ High	Critically low
Xiang,2021	MBO--I^{125}seeds+stent vs. stent	RCT(10)	1-year stent occlusion	OR, 0.15 [0.08; 0.31]	serious[a]	serious[b]	not serious	not serious	strongly suspected	yes	no	no	8-Critical	⊕⊕⊕◯ Moderate	Critically low
Xiang,2021	MBO--I^{125}seeds+stent vs. stent	RCT(9)	Survival	SMD, 0.98 [0.66; 1.31]	serious[a]	serious[b]	not serious	not serious	strongly suspected	no	no	no	8-Critical	⊕◯◯◯ Very Low	Critically low
Yuan,2019	MBO--PECMS/MSCPM vs. CMS	RCT(3)	Stent patency duration	HR, 1.30 [0.93; 1.83]	serious[a]	not serious	not serious	not serious	strongly suspected	no	no	no	7-Critical	⊕⊕◯◯ Low	Critically low
Yuan,2019	MBO--PECMS/MSCPM vs. CMS	RCT(3)	Survival time	HR, 0.85 [0.59; 1.21]	serious[a]	not serious	not serious	not serious	strongly suspected	no	no	no	7-Critical	⊕⊕◯◯ Low	Critically low
Yuan,2019	MBO--PECMS/MSCPM vs. CMS	RCT(3)	Stent malfunction	RR, 1.09 [0.72; 1.66]	serious[a]	not serious	not serious	not serious	undetected	no	no	no	6-Important	⊕⊕⊕◯ Moderate	Critically low
Yuan,2019	MBO--PECMS/MSCPM vs. CMS	RCT(3)	Stent occlusion caused by tumor in growth	RR, 1.09 [0.59; 2.03]	serious[a]	not serious	not serious	serious[c]	undetected	no	no	no	6-Important	⊕⊕◯◯ Low	Critically low
Yuan,2019	MBO--PECMS/MSCPM vs. CMS	RCT(3)	Stent occlusion caused by distal stent	RR, 0.92 [0.38; 2.20]	serious[a]	not serious	not serious	not serious	undetected	no	no	no	6-Important	⊕⊕⊕◯ Moderate	Critically low
Yuan,2019	MBO--PECMS/MSCPM vs. CMS	RCT(3)	Complications	RR, 1.17 [0.75; 1.83]	serious[a]	not serious	not serious	not serious	undetected	no	no	no	6-Important	⊕⊕⊕◯ Moderate	Critically low
Yuan,2019	MBO--PECMS/MSCPM vs. CMS	RCT(3)	Pancreatitis	RR, 0.58 [0.19; 1.79]	serious[a]	not serious	not serious	serious[c]	undetected	no	no	no	6-Important	⊕⊕◯◯ Low	Critically low
Yuan,2019	MBO--PECMS/MSCPM vs. CMS	RCT(3)	Cholangitis-like symptoms	RR, 1.80 [0.66; 4.94]	serious[a]	not serious	not serious	serious[c]	strongly suspected	no	no	no	6-Important	⊕◯◯◯ Very Low	Critically low
Song,2022	MBO--RFA+Stent vs. Stent alone	RCT(3)	Overall survival	HR, 0.41 [0.21; 0.78]	not serious	serious[b]	not serious	not serious	undetected	yes	no	no	8-Critical	⊕⊕⊕⊕ High	Low
Song,2022	MBO--RFA+Stent vs. Stent alone	RCT(3)	Mean survival time	SMD, 5.03 [0.94, 9.12]	not serious	serious[b]	not serious	serious[c]	strongly suspected	yes	no	no	8-Critical	⊕⊕⊕◯ Moderate	Low
Song,2022	MBO--RFA+Stent vs. Stent alone	RCT(3)	Mean stent patency time	SMD, 0.63 [-1.77; 3.04]	not serious	serious[b]	not serious	not serious	undetected	no	no	no	7-Critical	⊕⊕⊕◯ Moderate	Low
Song,2022	MBO--RFA+Stent vs. Stent alone	RCT(2)	Stent patency rate at 3 months	OR, 0.90 [0.36; 2.25]	not serious	not serious	not serious	not serious	strongly suspected	no	no	no	7-Critical	⊕⊕⊕◯ Moderate	Low
Song,2022	MBO--RFA+Stent vs. Stent alone	RCT(2)	Stent patency rate at 6 months	OR, 1.00 [0.44; 2.25]	not serious	not serious	not serious	not serious	strongly suspected	no	no	no	7-Critical	⊕⊕⊕◯ Moderate	Low
Song,2022	MBO--RFA+Stent vs. Stent alone	RCT(1)	Alleviation of total bilirubin	SMD, 0.50 [0.01; 1.00]	not serious	serious[b]	not serious	serious[c]	strongly suspected	no	no	no	7-Critical	⊕◯◯◯ Very Low	Low
Song,2022	MBO--RFA+Stent vs. Stent alone	RCT(1)	Alleviation of direct bilirubin	SMD, 0.16 [-0.33; 0.64]	not serious	serious[b]	not serious	not serious	strongly suspected	no	no	no	6-Important	⊕⊕◯◯ Low	Low
Song,2022	MBO--RFA+Stent vs. Stent alone	RCT(1)	Abdominal pain	OR, 2.07 [0.50; 8.57]	not serious	serious[b]	not serious	serious[c]	strongly suspected	no	no	no	6-Important	⊕◯◯◯ Very Low	Low
Song,2022	MBO--RFA+Stent vs. Stent alone	RCT(2)	Mild bleeding	OR, 0.33 [0.05; 2.12]	not serious	not serious	not serious	serious[c]	strongly suspected	no	no	no	6-Important	⊕⊕◯◯ Low	Low
Song,2022	MBO--RFA+Stent vs. Stent alone	RCT(3)	Cholangitis	OR, 0.82 [0.40; 1.66]	not serious	not serious	not serious	not serious	strongly suspected	no	no	no	6-Important	⊕⊕⊕◯ Moderate	Low
Song,2022	MBO--RFA+Stent vs. Stent alone	RCT(3)	Pancreatitis	OR, 0.54 [0.17; 1.71]	not serious	not serious	not serious	not serious	undetected	no	no	no	6-Important	⊕⊕⊕⊕ High	Low
Almadi,2017	MBO--Self-expandable metal stents vs. Plastic stents	RCT(4)	Duration of stent patency	SMD, 1.79 [-0.84; 4.42]	serious[a]	serious[b]	not serious	not serious	undetected	no	no	no	7-Critical	⊕⊕◯◯ Low	Critically low
Almadi,2017	MBO--Self-expandable metal stents vs. Plastic stents	RCT(5)	Duration of patient survival	SMD, 0.07 [-0.39; 0.54]	serious[a]	serious[b]	not serious	not serious	undetected	no	no	no	7-Critical	⊕⊕◯◯ Low	Critically low

Almadi,2017	MBO--Self-expandable metal stents vs. Plastic stents	RCT(8)	30day mortality	OR, 0.78 [0.49; 1.22]	serious[a]	not serious	not serious	not serious	undetected	no	no	no	7-Critical	⊕⊕⊕◯ Moderate	Critically low
Almadi,2017	MBO--Self-expandable metal stents vs. Plastic stents	RCT(12)	Successful stent insertion*(Fixed)	OR, 1.23 [0.66; 2.28]	serious[a]	not serious	not serious	not serious	undetected	no	no	no	7-Critical	⊕⊕⊕◯ Moderate	Critically low
Almadi,2017	MBO--Self-expandable metal stents vs. Plastic stents	RCT(8)	Successful biliary drainage	OR, 1.79 [0.64; 5.00]	serious[a]	serious[b]	not serious	serious[c]	undetected	no	no	no	7-Critical	⊕◯◯◯ Very Low	Critically low
Almadi,2017	MBO--Self-expandable metal stents vs. Plastic stents	RCT(14)	Early complications	OR, 0.65 [0.29; 1.42]	serious[a]	serious[b]	not serious	not serious	undetected	no	no	no	6-Important	⊕⊕◯◯ Low	Critically low
Almadi,2017	MBO--Self-expandable metal stents vs. Plastic stents	RCT(11)	Late complications	OR, 0.43 [0.26; 0.71]	serious[a]	serious[b]	not serious	not serious	undetected	yes	no	no	7-Critical	⊕⊕⊕◯ Moderate	Critically low
Almadi,2017	MBO--Self-expandable metal stents vs. Plastic stents	RCT(17)	Total complication	OR, 0.40 [0.22; 0.73]	serious[a]	serious[b]	not serious	not serious	undetected	yes	no	no	7-Critical	⊕⊕⊕◯ Moderate	Critically low
Almadi,2017	MBO--Self-expandable metal stents vs. Plastic stents	RCT(11)	Pancreatitis*(Fixed)	OR, 0.93 [0.48; 1.80]	serious[a]	not serious	not serious	not serious	undetected	no	no	no	6-Important	⊕⊕⊕◯ Moderate	Critically low
Almadi,2017	MBO--Self-expandable metal stents vs. Plastic stents	RCT(10)	Bleeding*(Fixed)	OR, 0.90 [0.39; 2.08]	serious[a]	not serious	not serious	not serious	undetected	no	no	no	6-Important	⊕⊕⊕◯ Moderate	Critically low
Almadi,2017	MBO--Self-expandable metal stents vs. Plastic stents	RCT(14)	Sepsis or cholangitis	OR, 0.53 [0.31; 0.90]	serious[a]	not serious	not serious	not serious	undetected	no	no	no	7-Critical	⊕⊕⊕◯ Moderate	Critically low
Almadi,2017	MBO--Self-expandable metal stents vs. Plastic stents	RCT(8)	Stent migration*(Fixed)	OR, 0.65 [0.31; 1.39]	serious[a]	not serious	not serious	not serious	undetected	no	no	no	6-Important	⊕⊕⊕◯ Moderate	Critically low
Almadi,2017	MBO--Self-expandable metal stents vs. Plastic stents	RCT(8)	Blockage from sludge*(Fixed)	OR, 0.11 [0.07; 0.17]	serious[a]	not serious	not serious	not serious	undetected	yes	no	no	7-Critical	⊕⊕⊕⊕ High	Critically low
Almadi,2017	MBO--Self-expandable metal stents vs. Plastic stents	RCT(7)	Tumor ingrowth*(Fixed)	OR, 11.66 [3.75; 36.26]	serious[a]	not serious	not serious	serious[c]	undetected	yes	no	no	7-Critical	⊕⊕⊕⊕ High	Critically low
Almadi,2017	MBO--Self-expandable metal stents vs. Plastic stents	RCT(7)	Tumor overgrowth*(Fixed)	OR, 1.90 [0.79; 4.58]	serious[a]	not serious	not serious	serious[c]	undetected	no	no	no	6-Important	⊕⊕◯◯ Low	Critically low
Almadi,2017	MBO--Self-expandable metal stents vs. Plastic stents	RCT(2)	Hospital re-admission*(Fixed)	OR, 1.10 [0.43; 2.85]	serious[a]	not serious	not serious	serious[c]	strongly suspected	no	no	no	6-Important	⊕◯◯◯ Very Low	Critically low
Almadi,2017	MBO--Self-expandable metal stents vs. Plastic stents	RCT(8)	Rate of re-interventions	OR, 0.37 [0.16; 0.81]	serious[a]	serious[b]	not serious	not serious	undetected	yes	no	no	7-Critical	⊕⊕⊕◯ Moderate	Critically low
Almadi,2017	MBO--Self-expandable metal stents vs. Plastic stents	RCT(5)	Mean number of re-interventions	WMD, −0.83 [−1.64; −0.02]	serious[a]	serious[b]	not serious	not serious	undetected	no	no	no	7-Critical	⊕⊕◯◯ Low	Critically low
Almadi,2017	MBO--Self-expandable metal stents vs. Plastic stents	RCT(4)	1-month symptom free	OR, 0.29 [0.02; 5.42]	serious[a]	serious[b]	not serious	serious[c]	undetected	yes	no	no	7-Critical	⊕◯◯◯ Very Low	Critically low
Almadi,2017	MBO--Self-expandable metal stents vs. Plastic stents	RCT(3)	3-month symptom free	OR, 2.16 [0.67; 6.98]	serious[a]	serious[b]	not serious	serious[c]	undetected	yes	no	no	7-Critical	⊕◯◯◯ Very Low	Critically low
Almadi,2017	MBO--Self-expandable metal stents vs. Plastic stents	RCT(4)	6-month symptom free	OR, 5.99 [1.67; 21.51]	serious[a]	serious[b]	not serious	serious[c]	undetected	yes	no	no	8-Critical	⊕⊕⊕◯ Moderate	Critically low
Almadi,2017	MBO--Self-expandable metal stents vs. Plastic stents	RCT(3)	12-month symptom free	OR, 2.90 [0.22; 37.65]	serious[a]	serious[b]	not serious	serious[c]	strongly suspected	no	no	no	7-Critical	⊕◯◯◯ Very Low	Critically low
Renno,2019	MBO--ARVMS vs. SEMS	RCT(3)	Technical success	OR, 0.13 [0.01; 1.06]	serious[a]	serious[b]	not serious	serious[c]	strongly suspected	no	no	no	6-Important	⊕◯◯◯ Very Low	Critically low
Renno,2019	MBO--ARVMS vs. SEMS	RCT(2)	Clinical success	OR, 1.30 [0.48; 3.51]	serious[a]	not serious	not serious	serious[c]	undetected	no	no	no	6-Important	⊕⊕◯◯ Low	Critically low
Renno,2019	MBO--ARVMS vs. SEMS	RCT(3)	All adverse events	OR, 0.62 [0.33; 1.17]	serious[a]	not serious	not serious	not serious	undetected	no	no	no	6-Important	⊕⊕⊕◯ Moderate	Critically low
Renno,2019	MBO--ARVMS vs. SEMS	RCT(2)	Early adverse events	OR, 0.70 [0.26; 1.85]	serious[a]	not serious	not serious	not serious	strongly suspected	no	no	no	6-Important	⊕⊕◯◯ Low	Critically low
Renno,2019	MBO--ARVMS vs. SEMS	RCT(2)	Late adverse events	OR, 0.45 [0.20; 1.03]	serious[a]	not serious	not serious	not serious	strongly suspected	no	no	no	6-Important	⊕⊕◯◯ Low	Critically low
Renno,2019	MBO--ARVMS vs. SEMS	RCT(3)	Overall stent dysfunction	OR, 0.77 [0.31; 1.93]	serious[a]	serious[b]	not serious	not serious	undetected	no	no	no	6-Important	⊕⊕◯◯ Low	Critically low
Renno,2019	MBO--ARVMS vs. SEMS	RCT(3)	Stent migration	OR, 2.69 [1.17; 6.15]	serious[a]	not serious	not serious	serious[c]	undetected	yes	no	no	7-Critical	⊕⊕⊕◯ Moderate	Critically low
Renno,2019	MBO--ARVMS vs. SEMS	RCT(3)	Stent occlusion	OR, 0.45 [0.25; 0.79]	serious[a]	not serious	not serious	not serious	strongly suspected	yes	no	no	7-Critical	⊕⊕⊕◯ Moderate	Critically low
Tringali,2018	MBO--Covered-SEMS vs.	RCT(8)	Stent failure	HR, 0.68 [0.40; 1.17]	serious[a]	serious[b]	not serious	serious[c]	strongly suspected	no	no	no	6-Important	⊕◯◯◯	Critically

	Uncovered-SEMS													Very Low	low
Tringali,2018	MBO--Covered-SEMS vs. Uncovered-SEMS	RCT(8)	Patient mortality	HR, 0.89 [0.76; 1.05]	serious[a]	not serious	not serious	not serious	strongly suspected	no	no	no	6-Important	⊕⊕◯◯ Low	Critically low
Tringali,2018	MBO--Covered-SEMS vs. Uncovered-SEMS	RCT(10)	Stent migration--C-SEMS/PC-SEMS vs.U-SEMS	OR, 7.70 [2.46; 24.12]	serious[a]	not serious	not serious	serious[c]	strongly suspected	yes	no	no	7-Critical	⊕⊕⊕◯ Moderate	Critically low
Tringali,2018	MBO--Covered-SEMS vs. Uncovered-SEMS	RCT(6)	Stent migration--C-SEMS vs.U-SEMS	OR, 10.60 [1.93; 58.30]	serious[a]	not serious	not serious	serious[c]	strongly suspected	yes	no	no	7-Critical	⊕⊕⊕◯ Moderate	Critically low
Tringali,2018	MBO--Covered-SEMS vs. Uncovered-SEMS	RCT(4)	Stent migration--PC-SEMS vs.U-SEMS	OR, 5.92 [1.27; 27.62]	serious[a]	not serious	not serious	serious[c]	strongly suspected	yes	no	no	7-Critical	⊕⊕⊕◯ Moderate	Critically low
Tringali,2018	MBO--Covered-SEMS vs. Uncovered-SEMS	RCT(9)	Tumor ingrowth--C-SEMS/PC-SEMS vs.U-SEMS	OR, 0.18 [0.07; 0.43]	serious[a]	not serious	not serious	not serious	strongly suspected	yes	no	no	7-Critical	⊕⊕⊕⊕ High	Critically low
Tringali,2018	MBO--Covered-SEMS vs. Uncovered-SEMS	RCT(5)	Tumor ingrowth--C-SEMS vs.U-SEMS	OR, 0.11 [0.03; 0.45]	serious[a]	not serious	not serious	not serious	strongly suspected	yes	no	no	7-Critical	⊕⊕⊕⊕ High	Critically low
Tringali,2018	MBO--Covered-SEMS vs. Uncovered-SEMS	RCT(4)	Tumor ingrowth--PC-SEMS vs.U-SEMS	OR, 0.26 [0.08; 0.84]	serious[a]	not serious	not serious	serious[c]	undetected	yes	no	no	7-Critical	⊕⊕⊕◯ Moderate	Critically low
Tringali,2018	MBO--Covered-SEMS vs. Uncovered-SEMS	RCT(9)	Tumor overgrowth--C-SEMS/PC-SEMS vs.U-SEMS	OR, 1.96 [1.13; 3.39]	serious[a]	serious[b]	not serious	not serious	undetected	no	no	no	7-Critical	⊕⊕◯◯ Low	Critically low
Tringali,2018	MBO--Covered-SEMS vs. Uncovered-SEMS	RCT(5)	Tumor overgrowth--C-SEMS vs.U-SEMS	OR, 1.75 [0.93; 3.28]	serious[a]	serious[b]	not serious	not serious	undetected	no	no	no	6-Important	⊕⊕◯◯ Low	Critically low
Tringali,2018	MBO--Covered-SEMS vs. Uncovered-SEMS	RCT(4)	Tumor overgrowth--PC-SEMSvs.U-SEMS	OR, 2.78 [0.92; 8.40]	serious[a]	serious[b]	not serious	serious[c]	undetected	no	no	no	6-Important	⊕◯◯◯ Very Low	Critically low
Tringali,2018	MBO--Covered-SEMS vs. Uncovered-SEMS	RCT(8)	Sludge formation--C-SEMS/PC-SEMSvs.U-SEMS	OR, 2.46 [1.37; 4.43]	serious[a]	serious[b]	not serious	not serious	undetected	yes	no	no	7-Critical	⊕⊕⊕◯ Moderate	Critically low
Tringali,2018	MBO--Covered-SEMS vs. Uncovered-SEMS	RCT(5)	Sludge formation--C-SEMS vs.U-SEMS	OR, 2.55 [1.29; 5.03]	serious[a]	serious[b]	not serious	serious[c]	undetected	yes	no	no	7-Critical	⊕⊕◯◯ Low	Critically low
Tringali,2018	MBO--Covered-SEMS vs. Uncovered-SEMS	RCT(3)	Sludge formation--PC-SEMS vs.U-SEMS	OR, 2.21 [0.69; 7.16]	serious[a]	serious[b]	not serious	serious[c]	undetected	no	no	no	6-Important	⊕◯◯◯ Very Low	Critically low
Tringali,2018	MBO--Covered-SEMS vs. Uncovered-SEMS	RCT(11)	Cholecystitis--C-SEMS/PC-SEMS vs.U-SEMS	OR, 1.50 [0.72; 3.14]	serious[a]	serious[b]	not serious	not serious	strongly suspected	no	no	no	6-Important	⊕◯◯◯ Very Low	Critically low
Tringali,2018	MBO--Covered-SEMS vs. Uncovered-SEMS	RCT(8)	Cholecystitis--C-SEMSvs.U-SEMS	OR, 1.32 [0.47; 3.76]	serious[a]	serious[b]	not serious	not serious	strongly suspected	no	no	no	6-Important	⊕◯◯◯ Very Low	Critically low
Tringali,2018	MBO--Covered-SEMS vs. Uncovered-SEMS	RCT(3)	Cholecystitis--PC-SEMSvs.U-SEMS	OR, 1.71 [0.60; 4.84]	serious[a]	serious[b]	not serious	serious[c]	strongly suspected	no	no	no	6-Important	⊕◯◯◯ Very Low	Critically low
Tringali,2018	MBO--Covered-SEMS vs. Uncovered-SEMS	RCT(4)	Cholangitis--C-SEMS/PC-SEMS vs. U-SEMS	OR, 0.95 [0.51; 1.79]	serious[a]	serious[b]	not serious	not serious	strongly suspected	no	no	no	6-Important	⊕◯◯◯ Very Low	Critically low
Tringali,2018	MBO--Covered-SEMS vs. Uncovered-SEMS	RCT(2)	Cholangitis--C-SEMS vs.U-SEMS	OR, 0.93 [0.45; 1.93]	serious[a]	serious[b]	not serious	not serious	strongly suspected	no	no	no	6-Important	⊕◯◯◯ Very Low	Critically low
Tringali,2018	MBO--Covered-SEMS vs. Uncovered-SEMS	RCT(2)	Cholangitis--PC-SEMS vs.U-SEMS	OR, 1.30 [0.14; 12.03]	serious[a]	serious[b]	not serious	serious[c]	strongly suspected	no	no	no	6-Important	⊕◯◯◯ Very Low	Critically low
Tringali,2018	MBO--Covered-SEMS vs. Uncovered-SEMS	RCT(10)	Pancreatitis--C-SEMS/PC-SEMS vs.U-SEMS	OR, 1.32 [0.59; 2.93]	serious[a]	not serious	not serious	not serious	undetected	no	no	no	6-Important	⊕⊕⊕◯ Moderate	Critically low
Tringali,2018	MBO--Covered-SEMS vs. Uncovered-SEMS	RCT(7)	Pancreatitis--C-SEMS vs.U-SEMS	OR, 0.93 [0.30; 2.84]	serious[a]	not serious	not serious	not serious	undetected	no	no	no	6-Important	⊕⊕◯◯ Low	Critically low
Tringali,2018	MBO--Covered-SEMS vs. Uncovered-SEMS	RCT(3)	Pancreatitis--PC-SEMS vs.U-SEMS	OR, 1.90 [0.60; 5.99]	serious[a]	not serious	not serious	serious[c]	undetected	no	no	no	6-Important	⊕⊕◯◯ Low	Critically low
Tringali,2018	MBO--Covered-SEMS vs. Uncovered-SEMS	RCT(4)	Perforation--C-SEMS/PC-SEMS vs.U-SEMS	OR, 1.76 [0.40; 7.68]	serious[a]	serious[b]	not serious	serious[c]	strongly suspected	no	no	no	6-Important	⊕◯◯◯ Very Low	Critically low
Tringali,2018	MBO--Covered-SEMS vs. Uncovered-SEMS	RCT(2)	Perforation--C-SEMSvs.U-SEMS	OR, 1.52 [0.18; 12.43]	serious[a]	serious[b]	not serious	serious[c]	strongly suspected	no	no	no	6-Important	⊕◯◯◯ Very Low	Critically low
Tringali,2018	MBO--Covered-SEMS vs. Uncovered-SEMS	RCT(2)	Perforation--PC-SEMSvs.U-SEMS	OR, 2.03 [0.26; 16.01]	serious[a]	serious[b]	not serious	serious[c]	strongly suspected	no	no	no	6-Important	⊕◯◯◯ Very Low	Critically low
Tringali,2018	MBO--Covered-SEMS vs. Uncovered-SEMS	RCT(6)	Bleeding--C-SEMS/PC-SEMS vs.U-SEMS	OR, 0.78 [0.24; 2.48]	serious[a]	serious[b]	not serious	not serious	strongly suspected	no	no	no	6-Important	⊕◯◯◯ Very Low	Critically low
Tringali,2018	MBO--Covered-SEMS vs. Uncovered-SEMS	RCT(3)	Bleeding--C-SEMSvs.U-SEMS	OR, 0.61 [0.14; 2.77]	serious[a]	serious[b]	not serious	not serious	strongly suspected	no	no	no	6-Important	⊕◯◯◯	Critically low

														Very Low	
Tringali,2018	MBO--Covered-SEMS vs. Uncovered-SEMS	RCT(3)	Bleeding--PC-SEMS vs.U-SEMS	OR, 1.09 [0.18; 6.81]	serious[a]	serious[b]	not serious	serious[c]	strongly suspected	no	no	no	6-Important	⊕○○○ Very Low	Critically low
Duan,2017	MBO--EUS-BD vs. PTBD	RCT(3)	Therapeutic success rate	OR, 2.33 [0.32; 17.12]	serious[a]	serious[b]	not serious	serious[c]	undetected	no	no	no	7-Critical	⊕○○○ Very Low	Critically low
Duan,2017	MBO--EUS-BD vs. PTBD	RCT(3)	Overall complication	OR, 1.56 [0.22; 11.12]	serious[a]	serious[b]	not serious	serious[c]	strongly suspected	no	no	no	6-Important	⊕○○○ Very Low	Critically low
Duan,2017	MBO--EUS-BD vs. PTBD	RCT(2)	30-day mortality rate	OR, 1.75 [0.28; 10.75]	serious[a]	not serious	not serious	serious[c]	strongly suspected	no	no	no	7-Critical	⊕○○○ Very Low	Critically low
Duan,2017	MBO--EUS-BD vs. PTBD	RCT(2)	Cholangitis	OR, 0.33 [0.06; 1.82]	serious[a]	serious[b]	not serious	serious[c]	strongly suspected	no	no	no	6-Important	⊕○○○ Very Low	Critically low
Sharaiha,2017	MBO--EUS-BD vs. PTBD	RCT(3)	Technical success	OR, 0.68 [0.14; 3.42]	serious[a]	not serious	not serious	serious[c]	undetected	no	no	no	6-Important	⊕⊕○○ Low	Critically low
Sharaiha,2017	MBO--EUS-BD vs. PTBD	RCT(2)	Clinical success	OR, 0.96 [0.24; 3.81]	serious[a]	not serious	not serious	serious[c]	strongly suspected	no	no	no	6-Important	⊕⊕○○ Low	Critically low
Sharaiha,2017	MBO--EUS-BD vs. PTBD	RCT(3)	Postprocedure adverse events	**OR, 0.25 [0.10; 0.61]**	serious[a]	not serious	not serious	not serious	strongly suspected	yes	no	no	7-Critical	⊕⊕⊕○ Moderate	Critically low
Sharaiha,2017	MBO--EUS-BD vs. PTBD	RCT(1)	Rate of re-intervention	**OR, 0.05[0.01; 0.25]**	serious[a]	serious[b]	not serious	serious[c]	strongly suspected	yes	no	no	7-Critical	⊕⊕○○ Low	Critically low
Sharaiha,2017	MBO--EUS-BD vs. PTBD	RCT(2)	Length of stay in hospital	**SMD, -1.04 [-1.50; -0.57]**	serious[a]	not serious	not serious	not serious	strongly suspected	no	no	no	7-Critical	⊕⊕○○ Low	Critically low
Logiudice,2019	MBO--EUS-BD vs. ERCP-BD	RCT(3)	Technical success	RR, 1.00 [0.93; 1.08]	not serious	not serious	not serious	not serious	undetected	no	no	no	6-Important	⊕⊕⊕⊕ High	Low
Logiudice,2019	MBO--EUS-BD vs. ERCP-BD	RCT(2)	Clinical success	RR, 0.99 [0.87; 1.13]	not serious	not serious	not serious	not serious	strongly suspected	no	no	no	6-Important	⊕⊕⊕○ Moderate	Low
Logiudice,2019	MBO--EUS-BD vs. ERCP-BD	RCT(3)	Duration of the procedure	SMD, -0.58 [-2.37; 1.21]	not serious	serious[b]	not serious	serious[c]	undetected	no	no	no	6-Important	⊕⊕○○ Low	Low
Logiudice,2019	MBO--EUS-BD vs. ERCP-BD	RCT(3)	Adverse events	RR, 0.67 [0.16; 2.79]	not serious	serious[b]	not serious	serious[c]	strongly suspected	no	no	no	6-Important	⊕○○○ Very Low	Low
Logiudice,2019	MBO--EUS-BD vs. ERCP-BD	RCT(2)	Stent patency	SMD, 0.08 [-0.62; 0.78]	not serious	not serious	not serious	not serious	strongly suspected	no	no	no	7-Critical	⊕⊕⊕○ Moderate	Low
Logiudice,2019	MBO--EUS-BD vs. ERCP-BD	RCT(3)	Stent dysfunction	**RR, 0.43 [0.24; 0.77]**	not serious	not serious	not serious	not serious	undetected	yes	no	no	7-Critical	⊕⊕⊕⊕ High	Low
Cui,2014	MBO--EST vs. no-EST	RCT(3)	Successful stent insertion	OR, 1.53 [0.42; 5.62]	serious[a]	serious[b]	not serious	serious[c]	strongly suspected	no	no	no	6-Important	⊕○○○ Very Low	Critically low
Cui,2014	MBO--EST vs. no-EST	RCT(3)	PEP	OR, 0.38 [0.10; 1.46]	serious[a]	not serious	not serious	serious[c]	strongly suspected	no	no	no	6-Important	⊕○○○ Very Low	Critically low
Cui,2014	MBO--EST vs. no-EST	RCT(2)	Post-ERCP bleeding	**OR, 9.52 [1.18; 76.93]**	serious[a]	not serious	not serious	serious[c]	strongly suspected	yes	no	no	7-Critical	⊕⊕⊕○ Moderate	Critically low
Cui,2014	MBO--EST vs. no-EST	RCT(2)	Stent migration	OR, 2.28 [0.34; 15.31]	serious[a]	not serious	not serious	serious[c]	strongly suspected	no	no	no	6-Important	⊕○○○ Very Low	Critically low
Cui,2014	MBO--EST vs. no-EST	RCT(3)	Stent occlusion	OR, 1.00 [0.38; 2.67]	serious[a]	not serious	not serious	serious[c]	strongly suspected	no	no	no	6-Important	⊕○○○ Very Low	Critically low
Fang,2013	Obstructive Jaundice-PBD--PBD vs. non-PBD	RCT(6)	Mortality	RR, 1.16 [0.75; 1.78]	not serious	not serious	not serious	not serious	undetected	no	no	no	7-Critical	⊕⊕⊕⊕ High	High
Fang,2013	Obstructive Jaundice-PBD--PBD vs. non-PBD	RCT(4)	Mortality--PTBD	RR, 1.22 [0.65; 2.29]	not serious	not serious	not serious	not serious	undetected	no	no	no	7-Critical	⊕⊕⊕⊕ High	High
Fang,2013	Obstructive Jaundice-PBD--PBD vs. non-PBD	RCT(2)	Mortality--ERCP	RR, 1.11 [0.62; 1.99]	not serious	not serious	not serious	not serious	strongly suspected	no	no	no	7-Critical	⊕⊕⊕○ Moderate	High
Fang,2013	Obstructive Jaundice-PBD--PBD vs. non-PBD	RCT(1)	Long-term mortality	HR, 0.90 [0.64; 1.24]	not serious	serious[b]	not serious	not serious	strongly suspected	no	no	no	7-Critical	⊕⊕○○ Low	High
Fang,2013	Obstructive	RCT(6)	Serious morbidity	**RR, 1.65 [1.21; 2.25]**	not	serious[b]	not serious	not serious	undetected	no	no	no	8-Critical	⊕⊕⊕○	High

	Jaundice-PBD--PBD vs. non-PBD				serious									Moderate	
Fang,2013	Obstructive Jaundice-PBD--PBD vs. non-PBD	RCT(4)	Serious morbidity--PTBD	RR, 1.79 [0.83; 3.87]	not serious	serious[b]	not serious	serious[c]	strongly suspected	no	no	no	7-Critical	⊕◯◯◯ Very Low	High
Fang,2013	Obstructive Jaundice-PBD--PBD vs. non-PBD	RCT(2)	Serious morbidity--ERCP	**RR, 1.75 [1.25; 2.44]**	not serious	serious[b]	not serious	not serious	strongly suspected	no	no	no	8-Critical	⊕⊕◯◯ Low	High
Fang,2013	Obstructive Jaundice-PBD--PBD vs. non-PBD	RCT(2)	Hospital stay	SMD, 0.37 [-0.15; 0.89]	not serious	serious[b]	not serious	not serious	strongly suspected	no	no	no	6-Important	⊕⊕◯◯ Low	High
Watanabe,2022	Periampullary Cancer-PBD--Metal stent vs. Plastic stent	RCT(6)	Re-intervention	**RR, 0.42 [0.25; 0.72]**	serious[a]	not serious	not serious	not serious	undetected	yes	no	no	7-Critical	⊕⊕⊕⊕ High	Critically low
Watanabe,2022	Periampullary Cancer-PBD--Metal stent vs. Plastic stent	RCT(5)	PBD-related complications	RR, 0.74 [0.32; 1.74]	serious[a]	serious[b]	not serious	not serious	undetected	no	no	no	6-Important	⊕⊕◯◯ Low	Critically low
Watanabe,2022	Periampullary Cancer-PBD--Metal stent vs. Plastic stent	RCT(6)	Postoperative complications	RR, 0.73 [0.45; 1.17]	serious[a]	serious[b]	not serious	not serious	strongly suspected	no	no	no	6-Important	⊕◯◯◯ Very Low	Critically low
Watanabe,2022	Periampullary Cancer-PBD--Metal stent vs. Plastic stent	RCT(2)	Direct costs	SMD, -0.64 [-2.27; 1.00]	serious[a]	serious[b]	not serious	serious[c]	strongly suspected	no	no	no	6-Important	⊕◯◯◯ Very Low	Critically low
Watanabe,2022	Periampullary Cancer-PBD--Metal stent vs. Plastic stent	RCT(6)	Stent occlusion	**OR, 0.29 [0.15; 0.57]**	serious[a]	not serious	not serious	not serious	undetected	yes	no	no	7-Critical	⊕⊕⊕⊕ High	Critically low
Watanabe,2022	Periampullary Cancer-PBD--Metal stent vs. Plastic stent	RCT(5)	Preoperative cholangitis	OR, 0.38 [0.08; 1.71]	serious[a]	not serious	not serious	not serious	undetected	no	no	no	6-Important	⊕⊕⊕◯ Moderate	Critically low
Watanabe,2022	Periampullary Cancer-PBD--Metal stent vs. Plastic stent	RCT(4)	Preoperative pancreatitis	OR, 2.40 [0.93; 6.20]	serious[a]	not serious	not serious	serious[c]	undetected	no	no	no	6-Important	⊕⊕◯◯ Low	Critically low
Watanabe,2022	Periampullary Cancer-PBD--Metal stent vs. Plastic stent	RCT(3)	Operative times	SMD, -0.33 [-0.81; 0.15]	serious[a]	not serious	not serious	not serious	undetected	no	no	no	6-Important	⊕⊕⊕◯ Moderate	Critically low
Watanabe,2022	Periampullary Cancer-PBD--Metal stent vs. Plastic stent	RCT(3)	Blood loss volumes	SMD, 0.62 [-1.52; 2.76]	serious[a]	serious[b]	not serious	serious[c]	undetected	no	no	no	6-Important	⊕◯◯◯ Very Low	Critically low
Watanabe,2022	Periampullary Cancer-PBD--Metal stent vs. Plastic stent	RCT(4)	Postoperative pancreatic fistulas	OR, 0.78 [0.28; 2.18]	serious[a]	not serious	not serious	serious[c]	undetected	no	no	no	6-Important	⊕⊕◯◯ Low	Critically low
Watanabe,2022	Periampullary Cancer-PBD--Metal stent vs. Plastic stent	RCT(5)	Delayed gastric emptying	OR, 1.30 [0.56; 3.00]	serious[a]	not serious	not serious	serious[c]	strongly suspected	no	no	no	6-Important	⊕◯◯◯ Very Low	Critically low
Watanabe,2022	Periampullary Cancer-PBD--Metal stent vs. Plastic stent	RCT(3)	Wound infection	OR, 3.00 [0.82; 10.96]	serious[a]	not serious	not serious	serious[c]	undetected	no	no	no	6-Important	⊕⊕◯◯ Low	Critically low
Watanabe,2022	Periampullary Cancer-PBD--Metal stent vs. Plastic stent	RCT(5)	Postoperative bleeding	OR, 0.64 [0.17; 2.33]	serious[a]	not serious	not serious	serious[c]	undetected	no	no	no	6-Important	⊕⊕◯◯ Low	Critically low

a: Failure to adequately control for confounding.
b: Conclusions significant heterogeneity was reported.
c: The credible interval contains invalid values and the credible interval does not exclude significant benefits or harms.

TableS10 Rcode: Random-efects model--Relative risk and 95% CIs、P-value、Heterogeneity and Small-study effects.

#RR/OR

```
>library(meta)
>setwd("F:\\~")
>data <- read.table("R.txt")
>names(data)[1:5]=c("Study","Ee","Ne","Ec","Nc")
>data
>summary(data$Ee/data$Ne)
>summary(data$Ec/data$Nc)
>mb3 <- metabin(Ee, Ne, Ec, Nc, sm="OR", method="I", data=data, studlab=Study)
>print(summary(mb3), digits=2)
>mb3 <- metabin(Ee, Ne, Ec, Nc, data=data, sm="OR")
>metabias(mb3, method="linreg",k.min = 2)
```

#logRR/logHR/logOR

```
>library(meta)
>setwd("F:\\~")
>data <- read.table("R.txt")
>names(data)[1:3]=c("Study","b","SE")
>data
>mg3 <- metagen(b, SE, studlab=paste(Study),data=data, sm="OR", backtransf=TRUE)
>summary(mg3)
>metabias(mg3, method="linreg",k.min = 2)
```

#MD/SMD

```
>library(meta)
>setwd("F:\\~")
>data <- read.table("R.txt")
>names(data)[1:7]=c("Study","Ne","Me","Se","Nc", "Mc", "Sc")
>data
>N <- with(data[1,], Ne + Nc)
>SMD <- with(data[1,], (1 - 3/(4 * N - 9)) * (data$Me - data$Mc) /sqrt(((data$Ne - 1)
        * data$Se*data$Se + (data$Nc - 1) * data$Sc*data$Se)/(N - 2)))
>seSMD <- with(data[1,], sqrt(N/(data$Ne * data$Nc) + SMD*SMD/(2 * (N - 3.94))))
>print(metacont(Ne, Me, Se, Nc, Mc, Sc, sm="SMD", data=data, subset=1), digits=2)
>weight <- 1/seSMD
>round(weighted.mean(SMD, weight), 4)
>round(1/sum(weight), 4)
>mc2 <- metacont(Ne, Me, Se, Nc, Mc, Sc, sm="SMD", data=data)
>print(summary(mc2), digits=2)
>metabias(mc2, method="linreg",k.min = 2)
```
